# Neural EGFL-like 1, a craniosynostosis-related osteochondrogenic molecule, strikingly associates with neurodevelopmental pathologies

**DOI:** 10.1186/s13578-023-01174-5

**Published:** 2023-12-15

**Authors:** Chenshuang Li, Zhong Zheng, Pin Ha, Wenlu Jiang, Chia Soo, Kang Ting

**Affiliations:** 1 Department of Orthodontics, School of Dental Medicine, University of Pennsylvania, Philadelphia, PA 19104 USA; 2 David Geffen School of Medicine, University of California, Los Angeles, Los Angeles, CA 90095 USA; 3 School of Dentistry, University of California, Los Angeles, Los Angeles, CA 90095 USA; 4 Orthopedic Hospital Research Center and David Geffen School of Medicine, University of California, Los Angeles, Los Angeles, CA 90095 USA; 5 American Dental Association Forsyth Institute, 245 First Street, Cambridge, MA 02142 USA; 6 MacDonald Research Laboratories (MRL), 675 Charles E. Young Dr. South Room 2641A, Box 951759, Los Angeles, CA 90095-1759 USA

**Keywords:** Neural EGFL-like 1, Neurodevelopmental, Autism spectrum disorder, Bone-brain-crosstalk

## Abstract

Various craniofacial syndromes cause skeletal malformations and are accompanied by neurological abnormalities at different levels, leading to tremendous biomedical, financial, social, and psychological burdens. Accumulating evidence highlights the importance of identifying and characterizing the genetic basis that synchronously modulates musculoskeletal and neurobehavioral development and function. Particularly, previous studies from different groups have suggested that neural EGFL-like-1 (Nell-1), a well-established osteochondrogenic inducer whose biopotency was initially identified in the craniofacial tissues, may also play a vital role in the central nervous system, particularly regarding neurological disorder pathologies. To provide first-hand behavior evidence if Nell-1 also has a role in central nervous system abnormalities, we compared the Nell-1-haploinsufficient (*Nell-1*^*+/6R*^) mice with their wild-type counterparts regarding their repetitive, social communication, anxiety-related, locomotor, sensory processing-related, motor coordination, and Pavlovian learning and memory behaviors, as well as their hippocampus transcriptional profile. Interestingly, *Nell-1*^*+/6R*^ mice demonstrated core autism spectrum disorder-like deficits, which could be corrected by Risperidone, an FDA-approved anti-autism, anti-bipolar medicine. Besides, transcriptomic analyses identified 269 differential expressed genes, as well as significantly shifted alternative splicing of ubiquitin B pseudogene *Gm1821*, in the *Nell-1*^*+/6R*^ mouse hippocampus, which confirmed that Nell-1 plays a role in neurodevelopment. Therefore, the current study verifies that Nell-1 regulates neurological development and function for the first time. Moreover, this study opens new avenues for understanding and treating craniofacial patients suffering from skeletal deformities and behavior, memory, and cognition difficulties by uncovering a novel bone-brain-crosstalk network. Furthermore, the transcriptomic analysis provides the first insight into deciphering the mechanism of Nell-1 in neurodevelopment.

**Supplementary Information:**

The online version contains supplementary material available at 10.1186/s13578-023-01174-5.

**Dear editor**

Craniofacial disorders manifest over 700 musculoskeletal malformations, such as craniosynostosis, with significant morbidity and mortality. Besides, survivors suffer from physical disfigurement, speech and hearing difficulties, developmental delays, and intellectual disabilities that reduce the overall quality of life. Taking isolated single suture craniosynostosis (SSC) as an example, 45% of patients display one or more speech, cognitive, and behavioral abnormal outcomes or a documented learning disability, special education placement, or identified behavioral problem. Unfortunately, existing treatments are often suboptimal because many craniofacial disorders have no clear pathoetiology to base treatments on. Since numerous craniofacial disorders co-display skeletal and neurodevelopment abnormalities in a “syndromic” fashion, a causal bone-brain relationship whereby skull deformations (e.g., craniosynostosis) that alter intracranial pressures lead to developmental central nervous system (CNS) abnormalities (e.g., neurocognitive impairment) was hypothesized. However, this paradigm is seriously challenged. First, the morphological correlations between the brain and skull are different, and the brain’s developmental trajectory is not in sync with the skull’s. In addition, the presence and significance of anatomical abnormalities in neurocognitive impairment [e.g., autism spectrum disorder (ASD)] is substantial controversy. Moreover, for craniosynostosis associated with neurodevelopmental aberration, such as isolated SSC, no consistent association between neurodevelopmental status and intracranial pressure was observed. Furthermore, abnormal CNS neurodevelopment can occur even after cranial vault reconstruction and restoration of normal intracranial pressures. Nevertheless, until identifying the genes capable of regulating both bone and brain development and understanding their function, the critical knowledge of the craniofacial disorder disease process and how to best treat these patients are largely lacking.

The neural EGFL-like 1 (Nell-1) gene was initially cloned from a human fetal brain cDNA library. Since the in vivo overexpression of *Nell-1* was associated with human craniosynostosis and cleidocranial dysostosis (CCD)-like skeletal abnormalities were noticed in neonatal mice with *Nell-1*-homodeficiency, Nell-1’s osteoinductive and chondrogenic potency has been endorsed in multiple animal models in the last two decades [1]. Some CCD patients exhibit cognitive disorders in adulthood, which suggests Nell-1 may also have neurodevelopmental roles; however, its function in the neural system has yet to be well investigated [1]. Beyond genome-wide association studies (GWAS) that revealed single-nucleotide polymorphism (SNP) of *Nell-1* frequently associated with a diversity of neural disorders (Additional file 1: Table S1), the only clue correlated Nell-1 with neurodevelopment is that extremely overexpressing Nell-1 causes massive neural cell apoptosis in developing mouse brain accompanied by acrania-like cranioskeletal deformities [1]. Noticeably, in our recent search for Nell-1’s receptor for osteogenesis, we identified contactin-associated protein-like 4 (Cntnap4) as a specific cell surface receptor for Nell-1 to execute its osteoinductive activity [2]. Cntnap4 is a transmembrane neurexin superfamily member essential for neurodevelopment, neurocognition, and neuropsychiatric disorders [3]. Global *Cntnap4*-knockout (KO) mice exhibit repetitive, ASD-like behaviors, which could be partially corrected by pharmacological dampening of dopaminergic signaling or augmentation of GABAergic signaling [3]. Zhang et al. also showed that *Cntnap4*-KO mice suffer from movement deficits [4]. Strikingly, our previous studies demonstrated direct Nell-1 and Cntnap4 binding in the human hippocampus [2], confirming the colocalization of these two molecules in the CNS.

To determine if Nell-1 also has a vigorous CNS role, we used *Nell-1*-haploinsufficient (*Nell-1*^*+/6R*^) mice, a well-established loss-of-function model [1], in the current study, as homozygous *Nell-1*-deficient mice die at birth. Restricted and persistent repetitive behaviors and impaired social communication are the two core behavioral characteristics of ASD [5]. For instance, over-grooming and excessive marble-burying are the two typical repetitive phenotypes in mice [5]. Echoing the previous studies that have revealed no significant bone malformations in *Nell-1*^*+/6R*^ mice during the development period from newborn to 6-month-old [6], microcomputed tomography (micro-CT) analyses revealed no significant deformity in the calvarial bone of 3-month-old *Nell-1*^*+/6R*^ mice (Additional file 2: Fig. S1**).** Surprisingly, 64% of the tested 3-month-old *Nell-1*^*+/6R*^ mice experienced hair loss due to overgrooming repetitive behavior (Fig. 1A). In contrast, none of the wildtype (WT) littermates experienced hair loss (Fig. 1A). Mild hair loss cases mostly occurred in whiskers, perinasal, and periorbital areas of *Nell-1*^*+/6R*^ mice (Fig. 1B), moderate cases exhibited large areas of facial hair loss (Fig. 1C), and in severe cases, full-body hair loss (Fig. 1D). The marble-burying test also confirmed repetitive behavior of *Nell-1*^*+/6R*^ mice, who buried significantly more marbles than WT littermates (Fig. 1E, and Additional file 3: Video S1). Meanwhile, *Nell-1*^*+/6R*^ mice did not display any significant differences from the WT animals in the open field arena test (Additional file 4, Fig. S2) and elevated plus-maze test (Additional file 5: Fig. S3)—the two routinely used methodologies for studying anxiety-related behaviors in mice. At the same time, the former also measures the animal’s locomotor activities [3, 4], suggesting that neither anxiety changes nor deficits in locomotion likely cause the observed repetitive behaviors.

**Fig. 1:**
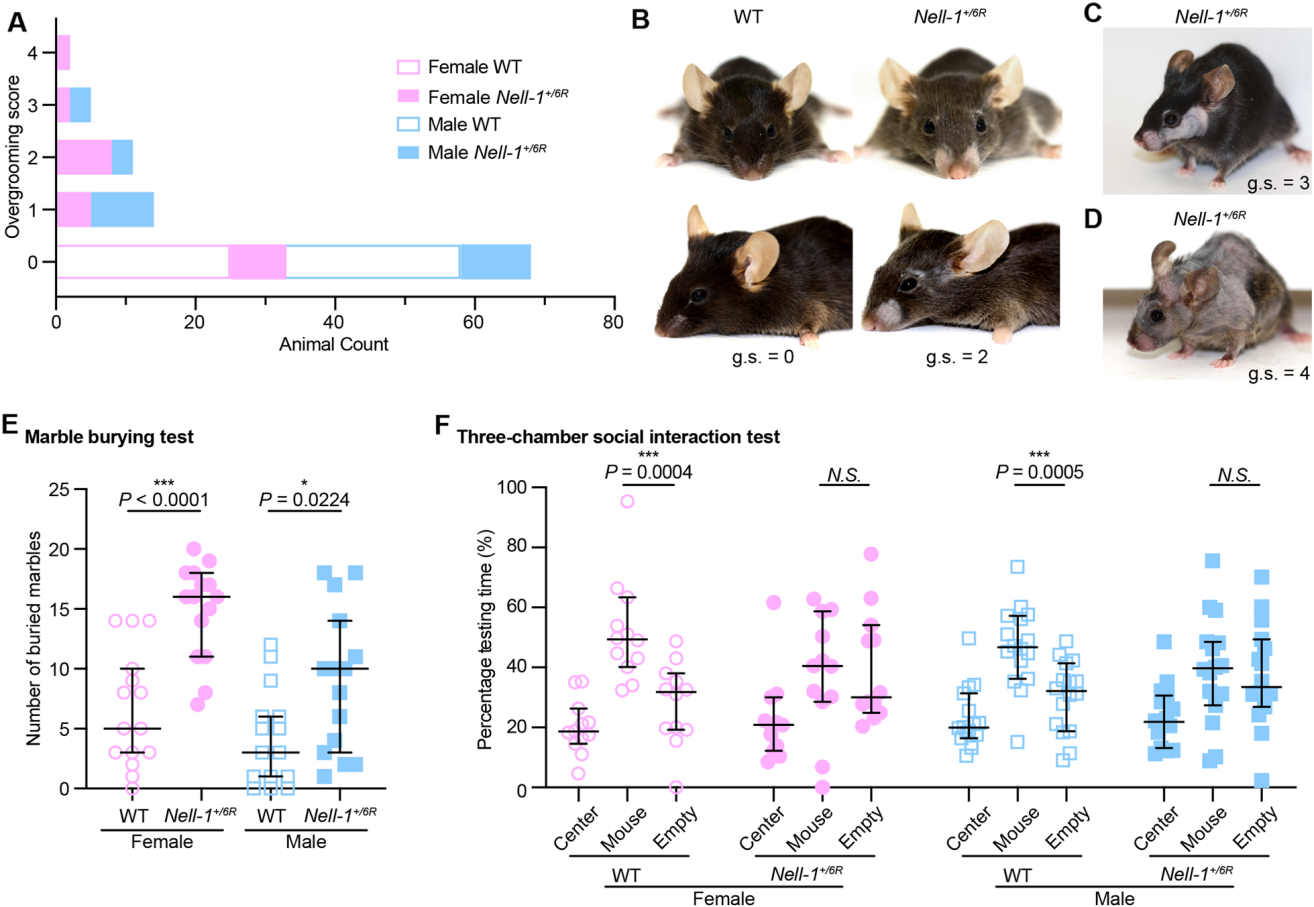
Three-month-old *Nell-1*^*+/6R*^ mice presented abnormal behavioral patterns. **A** The grooming scores were assigned to 50 three-month-old *Nell-1*^*+/6R*^ mice (25 females and 25 males) with their fifty WT littermates (25 females and 25 males). No statistical significance was detected between genders of the *Nell-1*^*+/6R*^ mice. **B** Typical appearance of facial hair and whisker loss in the areas around the nose and eyes (for which a score “2” was assigned in panel A ) due to repetitive overgrooming behavior seen in the majority of *Nell-1*^*+/6R*^ mice. WT mouse without overgrooming issue (for which a score “0” was assigned in panel A ) was also shown for comparison. g.s. = grooming score. **C** A three-month-old *Nell-1*^*+/6R*^ mouse represented a large area of facial hair loss due to overgrooming (for which a score “3” was assigned in panel A ). **D** A three-month-old *Nell-1*^*+/6R*^ mouse represented full body hair loss due to overgrooming (for which a score “4” was assigned in panel **A**). **E** Both male and female *Nell-1*^*+/6R*^  mice represented an increased number of buried marbles comparing to their WT littermates during a 10-minute marble-burying test. Data are presented as a median ± 95% confidence interval, n  = 15 for each group. **F** *Nell-1*^*+/6R*^  mice represented social behavior abnormalities in the three-chamber social interaction test. The time interacting with either an unfamiliar WT mouse (mouse cup) or an inanimate object (empty cup) in 10 min was shown in the figure in the format of the percentage of total testing time. Data are presented as median ± 95% confidence interval; n  = 12 (female) or 16 (male) for each genotype, respectively. Mann-Whitney *U* test was used for statistical analysis. *N.S*. none statistically significant. *: *P * < 0.05; ***: *P*  < 0.005

Noticeably, although *Nell-1*^*+/6R*^ mice have normal performance in a non-social setting (open field arena test [5]) (Additional file 4: Fig. S2), in a social setting (three-chamber social interaction test [5]), *Nell-1*^*+/6R*^ mice did not prefer a companion over the empty space when WT mice engaged more with other mice (Fig. 1F). As sensory processing is particularly important for social interactions [3], we performed pre-pulse inhibition (PPI) of the auditory startle reflex and found *Nell-1*^*+/6R*^ mice had normal startle response and PPI indexes (Additional file 6: Fig. S4). In addition, the rotarod test was used to examine the animal’s motor coordination [4] and detected no significantly different performance between *Nell-1*^*+/6R*^ and WT mice (Additional file 7: Fig. S5). Thus, the social-communicative impairment of *Nell-1*^*+/6R*^ mice does not appear to be stemmed from the defective ability to process sensory information or motor dysfunctions.

To the best of our knowledge, this is the first demonstration that *Nell-1*^*+/6R*^ mice, which exhibit a high risk of a broad spectrum of abnormal skeletal development and disease such as osteoporosis and arthritis (particularly at their senescent stage, e.g., 18-months old) [1, 6, 7], also start displaying neuropsychiatric abnormalities that strikingly represent ASD in humans at their young adult stage (e.g., 3-months old). Besides, as *Nell-1*^*+/6R*^ mice acted similarly to their WT counterparts in the fear conditioning test (Additional file 8: Fig. S6), *Nell-1*^*+/6R*^ mice may model an ASD subpopulation without Pavlovian learning and memory disability.

Previous studies have associated the dopaminergic pathway with ASD; thus, we next tested whether Risperidone, an FDA-approved anti-autism, anti-bipolar dopamine antagonist medicine, can reduce *Nell-1*^*+/6R*^ mice’s ASD-like behaviors. Since female mice exhibited more obvious behavioral irregularities than their male counterparts (Fig. 1A, E, and F), this initial proof-of-concept study focused on female *Nell-1*^*+/6R*^ mice. After 7-days of Risperidone treatment, the female *Nell-1*^*+/6R*^ mice reduced their numbers of marbles buried to the same level as their WT littermates (Fig. 2A). Risperidone also normalized the social interaction behavior of *Nell-1*^*+/6R*^ mice (Fig. 2B). The ability of Risperidone to significantly “cure” anomalous repetitive behaviors and impacted social interactions of female *Nell-1*^*+/6R*^ mice provide strong initial evidence for Nell-1’s vital role in neurological disorders and normal neurological function.

**Fig. 2:**
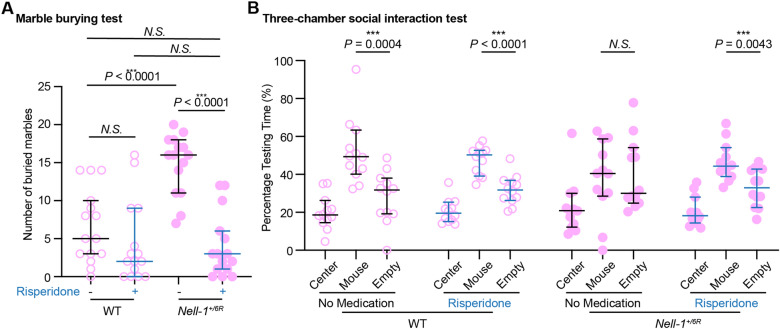
Anti-autism medication rescued *Nell-1*^*+/6R*^ mice from autism spectrum disorder-like behaviors. **A** Marble burying test: three-month-old *Nell-1*^*+/6R*^  mice and their WT littermates with or without intraperitoneal injections were tested. Data are presented as median 95% ± confidence interval; n  = 15 for each group. **B** Three-chamber social interaction test: percentage of total test time interaction with either an unfamiliar WT mouse (mouse cup) or an inanimate object (empty cup). Three-month-old *Nell-1*^*+/6R*^  mice and their WT littermates were tested with or without intraperitoneal injections. Data are presented as median ± 95% confidence interval, n  = 12 for each group. Mann-Whitney *U * test was used for statistical analysis. *N.S* .: none statistically significant. ***: *P * < 0.005

Since hippocampal pyramidal cells and interneurons may be critical for regulating excitatory (e.g., dopaminergic) and inhibitory (e.g., GABAergic) brain neurotransmitters to prevent neuropathology, hippocampus tissues of both *Nell-1*^*+/6R*^ and WT mice were then collected for transcriptomic analyses to gain more insight into Nell-1’s function in the CNS. The initial global transcriptomic analyses identified 269 differential expressed genes (DEGs) in the *Nell-1*^*+/6R*^ mouse hippocampus compared to their WT counterparts (Fig. 3A, and Additional file 9: Table S2). Noticeably, among the top 10 downregulated DEGs (Additional file 9: Table S2), several encode functional proteins in the nervous system. For example, acetylserotonin O-methyltransferase (encoded by *Asmt*) is the key rate-limiting enzyme of melatonin synthesis that has been reportedly associated with ASD [8], while *Asmt*-KO induced depression-like behaviors in mice [9]. In addition, reduced serum level of tryptase beta 2 (encoded by *Tpsb2*) is associated with worse cognitive performance in Alzheimer’s disease [10]. Meanwhile, lysyl oxidase (encoded by *Lox*) is an enzyme involved in the remodeling of the extracellular matrix whose expression is increased in Alzheimer’s disease [11] and is negatively associated with the prognosis of gliomas [12]. Besides, wingless-type MMTV integration site family, member 6 (encoded by *Wnt6*) is a protein expressed in the ectoderm which induces neural crest production during craniofacial development [13] while restoring Wnt6 signaling ameliorates the locomotor and social behavioral deficits in a mouse model of Rett syndrome [14]. Moreover, NK6 homeobox 1 (encoded by Nkx6-1) is exclusively expressed in astrocytes in the brainstem, regulates the astrocyte progenitor specification, migration, and maturation [15], and associates with the promoters of several brainstem-specific target genes [16]. On the other hand, among the top 10 upregulated DEGs (Additional file 9: Table S2), *Gm15577* specifically expresses in mice cerebellum in a developmentally regulated manner and modulates the expression of *Negr1*, a gene that has a distinct expression pattern between normal and medulloblastoma patients [17]. Additionally, *Slamf1* (encoding the signaling lymphocytic activation molecule family member 1) has been identified as a hub gene associated with molecular subtypes and immune regulation of ischaemic stroke [18]. Meanwhile, small nuclear ribonucleoprotein N (encoded by *Snrpn*) is highly expressed in all regions of the brain [19, 20], and its mutant has been demonstrated to be associated with Prader-Willi-Like Syndrome [19, 21, 22]. Remarkably, overexpression of *Snrpn* in mice shortened the length of neurites, resulted in a significant increase in spine dentistry at the distal ends of dendrites, and delayed radial migration in the cerebral cortex, which were assumed to contribute to defects in the potentiation of excitatory synaptic transmission [23], which is associated with intellectual disabilities and ASDs [24]. In addition, D-amino acid oxidase (enclosed by *Dao*) has been identified as a biomarker for mild cognitive impairment [25], whose inhibition has the potential to be a new therapeutic approach for the treatment of schizophrenia [26]. Furthermore, functional enrichment of the downregulated DEGs against a human gene database recognized *‘SLC-mediated transmembrane transport* (the main path for neural transmitter transmission)*’* (Fig. 3B, and Additional file 10: Table S3, Additional file 11: Table S4). At the same time, *‘Prader-Willi and Angelman syndrome,’* a genetic disorder with both musculoskeletal and neuropsychiatric abnormalities, is the top event enriched from the upregulated DEGs (Fig. 3C, and Additional file 12: Table S5, Additional file 13: Table S6).

**Fig. 3:**
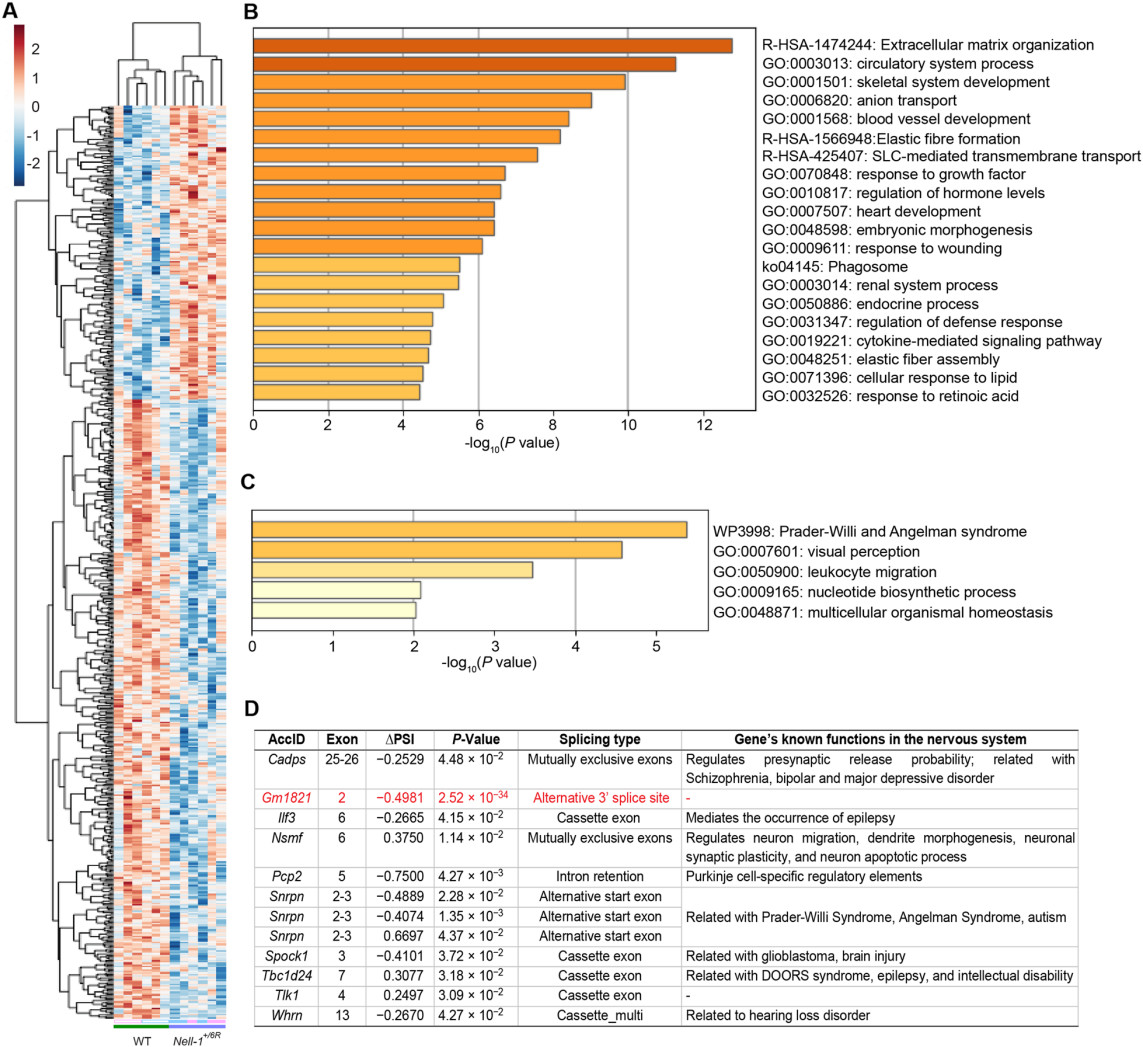
Global transcriptomic analyses revealed the differences between *Nell-1*^*+/6R*^ mouse hippocampus and their WT counterparts. **A** The heatmap visualizes the Nell-1-responsive differential expressed genes (DEGs) in the mouse hippocampus. N  = 6 (3 males [labeled as blue at the bottom] and 3 females [labeled as pink at the bottom]) for each genotype. **B** The top 20 of the downregulated DEGs enriched pathways with a *P*-value less than 0.01. **C** The upregulated DEGs enriched pathways with a *P*-value less than 0.01. **D** Alternative splicing (AS) analysis results with a *P*-value less than 0.05. The alternative splicing event with a false discovery rate of less than 0.05 is highlighted in red

It is worth noting that the majority of the genes have been found to exhibit alternatively spliced isoforms, which significantly increase the diversity of the transcriptome and markedly modulate their functions [27, 28]. Thus, we also explored the influence of *Nell-1*-haploinsufficient on mouse hippocampus gene alternative splicing (Additional file 14, Fig. S7) and identified ten genes with significant (*P* < 0.05, |ΔPSI| > 0.2) alternative splicing events, most of which have known functions in bipolar, epilepsy, autism, and craniofacial syndromes (Additional file 15: Table S7). The transcriptomic analyses support Nell-1 as an essential regulator of both skeletal development and neurodevelopment. Interestingly, we only detected *Gm1821*, a ubiquitin B (UBB) pseudogene with yet unknown functions, displaying alternative splicing events with a false detection rate (FDR) less than 0.05 (Fig. 3D).

Collectively, by demonstrating the ASD-like behaviors, the capability of anti-autism medication for ‘cure,’ and differential transcriptional profile in the hippocampus of *Nell-1*-haploinsufficient mice when compared to the WT littermates, we provided first-hand evidence that Nell-1 has vital neurodevelopment roles, particularly in neuropsychiatric disorders in the current study. Furthermore, given our accumulative studies validating the provoke role of Nell-1 in bone and cartilage development and regeneration [1] and the current research demonstrating the functional neuropsychiatric role of Nell-1, we believe that a single molecule, Nell-1, exerts significant roles in both musculoskeletal and neural system development and function. Therefore, our studies may qualify Nell-1 as one essential component of the entire bone-brain crosstalk network [29], while the underlying mechanisms remain to be systematically investigated. Recently, we identified Nell-1 as a novel ligand for Cntnap4 and demonstrated the importance of Cntnap4 for Nell-1-medicated osteogenesis [2]. Particularly, targeted inactivation of either Nell-1 or Cntnap4 in cranial neural crest cells led to remarkably similar defects in the calvarial bones [2]. As a presynaptic molecule, Cntnap4 also modulates neural progenitor cells’ proliferation and neuronal differentiation [30] and regulates dopaminergic and GABAergic synaptic transmission [3]. Thus, global *Cntnap4*-KO led to core ASD-like deficits in mice [3]. Interestingly, *Nell-1*^*+/6R*^ mice also exhibited repetitive, ASD-like behaviors, which can be corrected by pharmacological dampening of dopaminergic signaling. Meanwhile, both *Nell-1*^*+/6R*^ mice and *Cntnap4*-KO mice acted normally in the anxiety tests. Considering the colocalization of Nell-1 and Cntnap4 in hippocampal cells [2], the Nell-1/Cntnap4 functional axis may provide a novel signaling framework for both neural tissue and craniofacial neuro-skeletal interface investigation during development and growth in both health and pathogenic scenarios [29]. No doubt, the molecular events after Nell-1/Cntnap4 binding in the hippocampus [2] remain to be elucidated.

On the other hand, despite many similarities *Nell-1*^*+/6R*^ and *Cntnap4*-KO mice share, their behaviors are not identical. For instance, unlike *Cntnap4*-KO mice [3, 4], *Nell-1*^*+/6R*^ mice had normal startle response and PPI activity. Moreover, no significant difference was found in the rotarod performance (assessing dopaminergic synapse-responsive motor learning function) or fear learning in trace and delay conditioning (representing the structural and functional plasticity of GABAergic synapses) between *Nell-1*^*+/6R*^ and WT mice. One possibility is *Nell-1*^*+/6R*^ mice are only a *Nell-1*-haploinsufficient model, and one copy of the *Nell-1* gene is sufficient enough to maintain the normal sensory processing and motor coordination function, as well as the learning and memory performance, while the function of dopaminergic and GABAergic synapses are both substantially dampened in *Cntnap4*-KO mice [3, 4]. However, female *Nell-1*^*+/6R*^ mice spent slightly less time in the closed arms relative to the open arms compared to WT controls in the elevated plus maze test (Additional file 5: Fig. S3C), which was not noticed in *Cntnap4*-KO mice [3]. Moreover, agreed with the gene profiling that associated Nell-1 with Alzheimer’s disease, we noticed that older *Nell-1*^*+/6R*^ mice exhibited severe seizure episodes spontaneously and frequently (Additional file 16: Video S2, Additional file 17: Video S3, Additional file 18: Video S4), which were not reported among *Cntnap4*-KO animals either. These distinctly different behavioral phenotypes between *Nell-1*^*+/6R*^ and *Cntnap4*-KO mice strongly suggest an alternative hypothesis that Nell-1’s neural function is neither fully replicated nor entirely reliant on Cntnap4-responsive synaptic transmission. Particularly, the second theory is more aligned with previous observations that, to manifest its diverse biopotencies, Nell-1 may employ additional receptors except for Cntnap4 [2], which warrants further investigation as well.

Besides, as an 810 amino-acid protein with a postulated homopentamer structure [1], Nell-1 is not expected to penetrate the blood-brain barrier. Thus, Nell-1 may simultaneously but independently orchestrate CNS and craniofacial skeletal growth and development. However, *‘Skeletal system development*’ was recognized in the functional enrichment of the identified DEGs (Fig. 3B and Additional file 11: Table S4), although only hippocampus tissue was used for transcriptomic profiling. It is possible that some of Nell-1’s downstream effector(s), which need to be identified in the future, can pass the blood-brain barrier and exert the skeletal modulating effects. Therefore, unlike osteocalcin, which can directly cross the blood-brain barrier [31], Nell-1 may represent another subset of proteins involved in the bone-brain-crosstalk network.

Although they often lack known functions, growing numbers of pseudogenes are being found to play important biological roles [32]. Importantly, knowledge concerning pseudogenes has currently substantially increased due to the availability of high-throughput sequencing techniques, which confirms that pseudogenes have a variety of functions at the DNA, RNA, and protein levels for broadly participating in gene regulation to influence the development and progression of certain diseases [33]. Meanwhile, an aberrant form of UBB was associated with multiple neurodevelopmental disorders (e.g., Alzheimer’s disease, Parkinson’s disease, Huntington’s disease, Pick’s disease, Down’s syndrome, other tauopathies, and polyglutamine diseases). Importantly, having a suitable concentration of the correct form of UBB is essential for maintaining normal ATP synthesis, reactive oxygen species (ROS) generation, and mitochondria organization and function in neurons and astrocytes [34]. Considering pseudogenes generally regulate their protein-coding cousins [33], recognizing *Gm1821* alternative splicing events in *Nell-1*^*+/6R*^ mouse hippocampus may be more than coincidental. Rather, it is possible that Nell-1 modulates UBB signaling by controlling *Gm1821* splicing in the neurons and astrocytes to regulate neurological development and function [34], which provides important insight into the previously overlooked influence of pseudogene alternative splicing [32, 33].

It is worth noting that, besides neural or skeletal-related biological processes, immune-related biological processes have also been enriched, including ‘*leukocyte migration*,’ ‘*phagosome*,’ ‘*regulation of defense response*,’ and ‘*cytokine-mediated signaling*’ (Fig. 3B-C, Additional file 11: Table S4, and Additional file 13: Tables S6). Recently, the term ‘neuro-immuno-skeletal system’ has raised more attention as patients with various syndromes display deficiencies in all these three systems [35, 36]. Thus, the involvement of Nell-1 in the crosstalk among all three systems is also an area to be further explored.

In summary, the current preliminary study on Nell-1’s dual roles in musculoskeletal and neural systems may open new avenues for understanding the pathoetiology of craniofacial disorders and developing more effective, targeted therapeutics. No doubt, much more studies are needed to fully understand and confirm Nell-1’s role in neuropsychiatry, as well as compare the biopotency of Nell-1 and Cntnap4, including but not limited to the use of conditional-KO animal models, viral or other vector-medicated Nell-1 and/or Cntnap4 restoration. However, we hope the current investigation initiates a worldwide collaboration on potential neuropharmaceutical applications of Nell-1—a considerable safe agent currently under a clinical trial for degenerative disc disease and spondylolisthesis (ClinicalTrials.gov Identifier: NCT03810573).

### Supplementary Information

**Additional file 1: Table S1.** Known Nell-1 SNP correlated with neurodevelopmental disorders.

**Additional file 2: Fig. S1.** Micro-CT analyses revealed no significant calvarial bone malformations in 3-month-old *Nell-1*^+/6R^  mice.

**Additional file 3: Video S1.**The representative video of a pair of 3-month-old female *Nell-1*^*+/6R*^ mouse and the WT littermate in a 10-minute marble-burying test.

**Additional file 4: Fig. S2.**The *Nell-1*^*+/6R*^ mice did not represent major changes in anxiety levels as indicated by the open field arena (OFA) test. The total travel distance (A) and time spent in periphery versus center in two different central percentage calculations (66% in B, and 50% in C) are presented. No difference was found between *Nell-1*^*+/6R*^ mice and their WT littermates for both genders. Data are presented as median ± 95% confidence interval, N= 16 for each group. Mann-Whitney *U* test was used for statistical analysis. N.S.: none statistically significant.

**Additional file 5: Fig. S3.**Female *Nell-1*^*+/6R*^ mice but not the males presented impaired anxiety level in the elevated plus maze test. The total travel distance (A), the duration of stretched attend posture (SAP, B), and time spent in open versus closed arms (C) are presented. No difference was found between 3-month-old male *Nell-1*^*+/6R*^ mice and their WT littermates. On the other hand, female *Nell-1*^*+/6R*^ mice spent slightly less time on the closed arms than their WT counterparts, while no difference was found in other parameters. Data are presented as median ± 95% confidence interval, N= 16 for each group. Mann-Whitney *U* test was used for statistical analysis. N.S.: none statistically significant. *: *P* < 0.05.

**Additional file 6: Fig. S4.**The *Nell-1*^*+/6R*^ mice did not represent major changes in sensorimotor integration as indicated by the pre-pulse inhibition (PPI) test. The mean of the first 6, middle 10, and last 6 startle at 120 dB (A) and the percentage of PPI at 74, 82, 90 dB (B) are presented. No difference was found between *Nell-1*^*+/6R*^ mice and their WT littermates for both genders. Data are presented as median ± 95% confidence interval, N = 14 (female) or 16 (male) mice per genotype, respectively. Mann-Whitney *U* test was used for statistical analysis. N.S.: none statistically significant.

**Additional file 7: Fig. S5.**The *Nell-1*^*+/6R*^ mice did not represent major changes in motor coordination as indicated by the Rotarod performance test. (A) The length of latency to fall for 3-month-old *Nell-1*^*+/6R*^ mice and their WT littermates, as well as (B) the revolutions per minute (rpm) for 3-month-old *Nell-1*^*+/6R*^ mice and their WT counterparts are presented. No difference was found between *Nell-1*^*+/6R*^ mice and their WT littermates for both genders. Data are presented as median ± interquartile range, N = 16 for each group. Mann-Whitney *U* test was used for statistical analysis.

**Additional file 8: Fig. S6.**The *Nell-1*^*+/6R*^ mice did not represent major changes in learning and memory as indicated by the fear conditioning test. The baseline (BL, A), percentage time of freezing behavior during the total testing time of context fear (B), tone (C), and trace (D) for both trace and delay fear conditioning tests of 3-month-old *Nell-1*^*+/6R*^ mice and their WT littermates are presented. No difference was found between *Nell-1*^*+/6R*^ mice and their WT counterparts for both genders. Data are presented as median ± 95% confidence interval. In the trace conditioning test, N= 16 (female) or 8 (male) for each genotype; in the delay conditioning test, N = 14 (female) or 8 (male) for each genotype, respectively. Mann-Whitney *U* test was used for statistical analysis. N.S.: none statistically significant.

**Additional file 9: Table S2.**The list of differentially expressed genes (DEGs) in the hippocampus from *Nell-1*^*+/6R*^ mice and their wild-type littermates.

**Additional file 10: Table S3.**The converting results of the input downregulated DEGs in the Metascape.

**Additional file 11: Table S4.**The result of pathway and process enrichment analysis with the downregulated DEGs.

**Additional file 12: Table S5.**The converting results of the input upregulated DEGs in the Metascape.

**Additional file 13: Table S6.**The result of pathway and process enrichment analysis with the upregulated DEGs.

**Additional file 14: Fig. S7. **The demography of alternative splice events detected by CASH.

**Additional file 15: Table S7.**The alternative splicing gene list with a *P*-value less than 0.05.

**Additional file 16: Video S2.**A 12-month-old female *Nell-1*^*+/6R*^ mouse experienced seizures during a daily mouse check.

**Additional file 17: Video S3.**A cage of four 18-month-old female *Nell-1*^*+/6R*^ mice. Two of the mice experienced seizures during a daily mouse check.

**Additional file 18: Video S4.**A cage of four 18-month-old male *Nell-1*^*+/6R*^ mice. Two of the mice experienced seizures during a daily mouse check.

## Data Availability

The datasets in this study are available from the corresponding authors upon reasonable request. The RNA-Seq data were submitted to the NIH Gene Expression Omnibus (GEO GSE180856).

## Abbreviations

ASD: Autism spectrum disorder
CCD: Cleidocranial dysostosis
CNS: Central nervous system
Cntnap4: Contactin-associated protein-like 4
DEGs: Differential expressed genes
FDR: False detection rate
GWAS: Genome-wide association studies
KO: Knockout
Micro-CT: Microcomputed tomography
Nell-1: Neural EGFL-like-1
PPI: Pre-pulse inhibition
ROS: Reactive oxygen species
SNP: Single-nucleotide polymorphism
SSC: Single suture craniosynostosis
UBB: Ubiquitin B
WT: Wild-type
