# Supplementary material for: Neural EGFL-like 1, a craniosynostosis-related osteochondrogenic molecule, strikingly associates with neurodevelopmental pathologies
Source: Cell Biosci. 2023 Dec 15;13:227. doi: 10.1186/s13578-023-01174-5 (PMC10725010; doi:10.1186/s13578-023-01174-5)
Supplement: Supplementary file 1 — Additional file 1: Table S1. Known Nell-1 SNP correlated with neurodevelopmental disorders. [file 13578_2023_1174_MOESM1_ESM.docx]

**Table S1. Known Nell-1 SNP correlated with neurodevelopmental disorders.**

| **Diagnosis** | **SNP** | **Loci** | **Reference** |
| --- | --- | --- | --- |
| Autism spectrum disorder | rs1429793 | Intron 5 | (36) |
| Bipolar disorder | rs10766743 | Intron 5 | (37) |
| Major depressive disorder | rs2139423 | Intron 2 | (38) |
| Multiple sclerosis | rs7130553 | Intron 15 | (39) |
