# Supplementary material for: Neural EGFL-like 1, a craniosynostosis-related osteochondrogenic molecule, strikingly associates with neurodevelopmental pathologies
Source: Cell Biosci. 2023 Dec 15;13:227. doi: 10.1186/s13578-023-01174-5 (PMC10725010; doi:10.1186/s13578-023-01174-5)
Supplement: Supplementary file 2 — Additional file 2: Fig. S1. Micro-CT analyses revealed no significant calvarial bone malformations in 3-month-old Nell-1+/6R mice. [file 13578_2023_1174_MOESM2_ESM.docx]

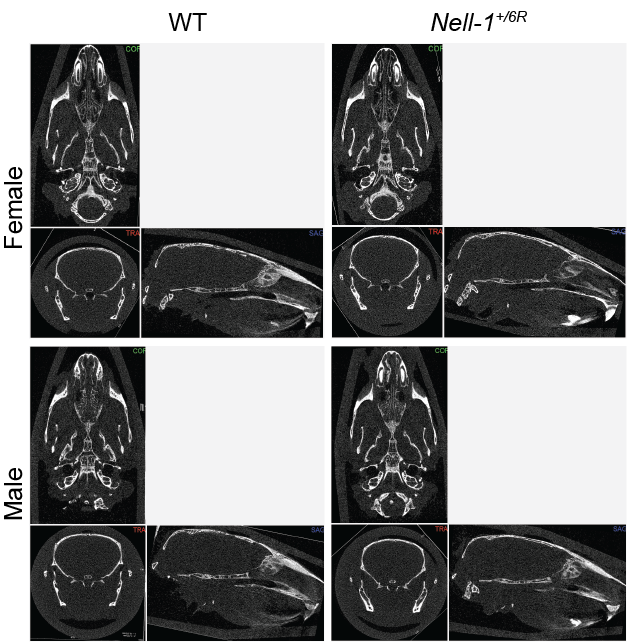


**Fig. S1**: Micro-CT analyses revealed no significant calvarial bone malformations in 3-month-old *Nell-1^+/6R^* mice.
