# Supplementary material for: Neural EGFL-like 1, a craniosynostosis-related osteochondrogenic molecule, strikingly associates with neurodevelopmental pathologies
Source: Cell Biosci. 2023 Dec 15;13:227. doi: 10.1186/s13578-023-01174-5 (PMC10725010; doi:10.1186/s13578-023-01174-5)
Supplement: Supplementary file 4 — Additional file 4: Fig. S2.The Nell-1+/6R mice did not represent major changes in anxiety levels as indicated by the open field arena (OFA) test. The total travel distance (A) and time spent in periphery versus center in two different central percentage calculations (66% in B, and 50% in C) are presented. No difference was found between Nell-1+/6R mice and their WT littermates for both genders. Data are presented as median ± 95% confidence interval, N= 16 for each group. Mann-Whitney U test was used for statistical analysis. N.S.: none statistically significant. [file 13578_2023_1174_MOESM4_ESM.docx]

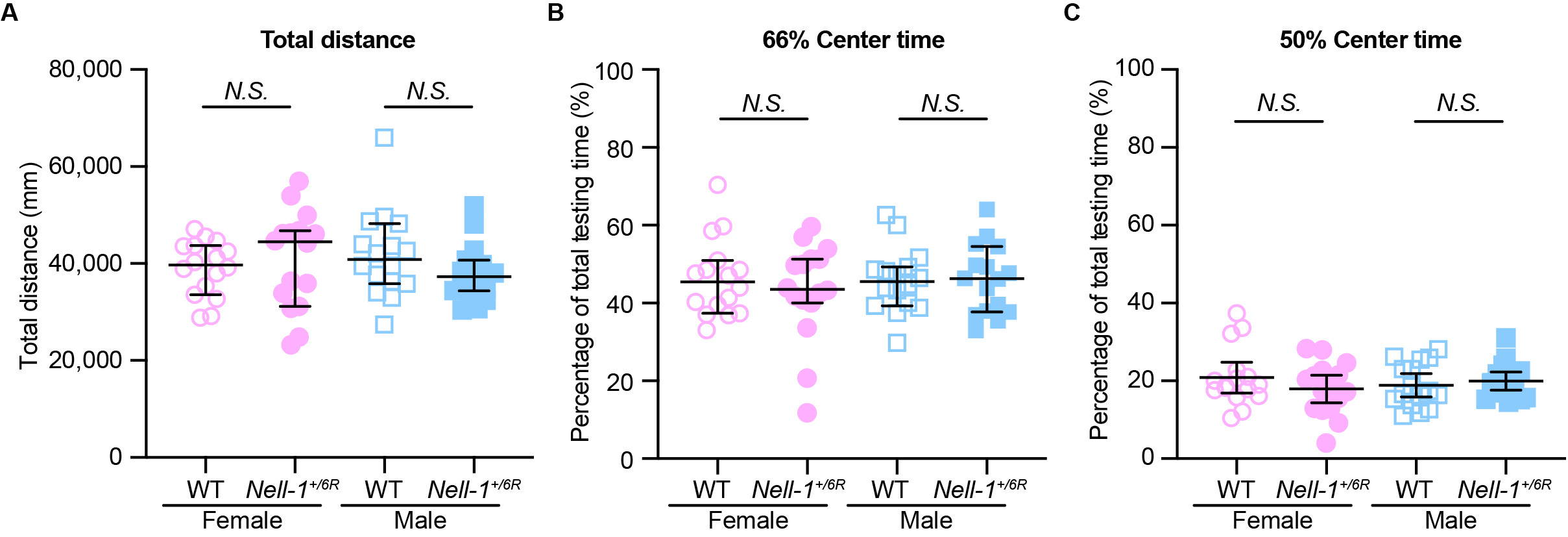


Fig. S2. The Nell-1^+/6R^ mice did not represent major changes in anxiety levels as indicated by the open field arena (OFA) test.

The total travel distance (A) and time spent in periphery versus center in two different central percentage calculations (66% in B, and 50% in C) are presented. No difference was found between Nell-1^+/6R^ mice and their WT littermates for both genders. Data are presented as median ± 95% confidence interval, N = 16 for each group. Mann-Whitney U test was used for statistical analysis. N.S.: none statistically significant.
