# Supplementary material for: Neural EGFL-like 1, a craniosynostosis-related osteochondrogenic molecule, strikingly associates with neurodevelopmental pathologies
Source: Cell Biosci. 2023 Dec 15;13:227. doi: 10.1186/s13578-023-01174-5 (PMC10725010; doi:10.1186/s13578-023-01174-5)
Supplement: Supplementary file 5 — Additional file 5: Fig. S3.Female Nell-1+/6R mice but not the males presented impaired anxiety level in the elevated plus maze test. The total travel distance (A), the duration of stretched attend posture (SAP, B), and time spent in open versus closed arms (C) are presented. No difference was found between 3-month-old male Nell-1+/6R mice and their WT littermates. On the other hand, female Nell-1+/6R mice spent slightly less time on the closed arms than their WT counterparts, while no difference was found in other parameters. Data are presented as median ± 95% confidence interval, N= 16 for each group. Mann-Whitney U test was used for statistical analysis. N.S.: none statistically significant. *: P < 0.05. [file 13578_2023_1174_MOESM5_ESM.docx]

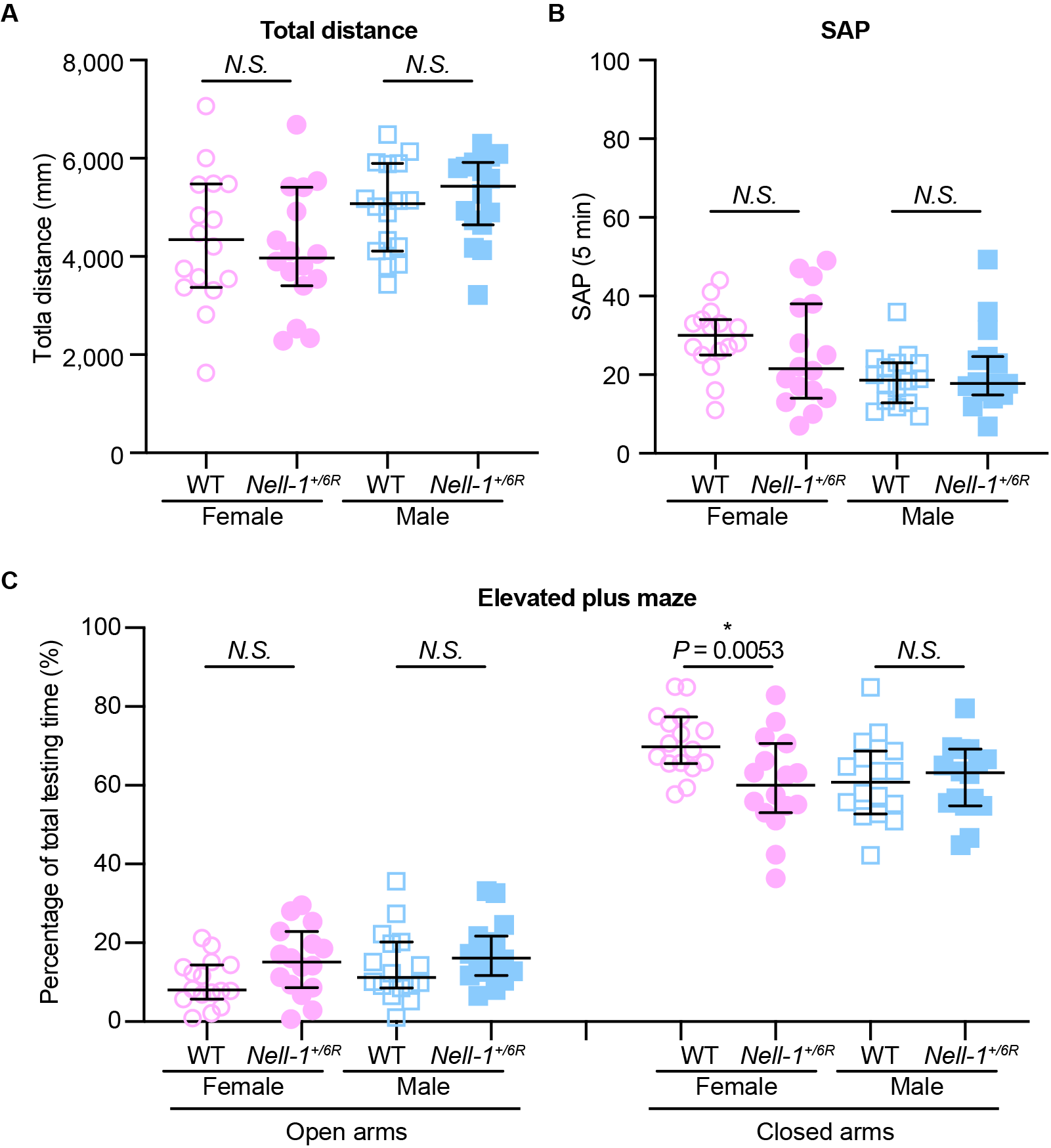


Fig. S3. Female Nell-1^+/6R^ mice but not the males presented impaired anxiety level in the elevated plus maze test.

The total travel distance (A), the duration of stretched attend posture (SAP, B), and time spent in open versus closed arms (C) are presented. No difference was found between 3-month-old male Nell-1^+/6R^ mice and their WT littermates. On the other hand, female Nell-1^+/6R^ mice spent slightly less time on the closed arms than their WT counterparts, while no difference was found in other parameters. Data are presented as median ± 95% confidence interval, N = 16 for each group. Mann-Whitney U test was used for statistical analysis. N.S.: none statistically significant. *: P < 0.05.
