# Supplementary material for: Neural EGFL-like 1, a craniosynostosis-related osteochondrogenic molecule, strikingly associates with neurodevelopmental pathologies
Source: Cell Biosci. 2023 Dec 15;13:227. doi: 10.1186/s13578-023-01174-5 (PMC10725010; doi:10.1186/s13578-023-01174-5)
Supplement: Supplementary file 6 — Additional file 6: Fig. S4.The Nell-1+/6R mice did not represent major changes in sensorimotor integration as indicated by the pre-pulse inhibition (PPI) test. The mean of the first 6, middle 10, and last 6 startle at 120 dB (A) and the percentage of PPI at 74, 82, 90 dB (B) are presented. No difference was found between Nell-1+/6R mice and their WT littermates for both genders. Data are presented as median ± 95% confidence interval, N = 14 (female) or 16 (male) mice per genotype, respectively. Mann-Whitney U test was used for statistical analysis. N.S.: none statistically significant. [file 13578_2023_1174_MOESM6_ESM.docx]

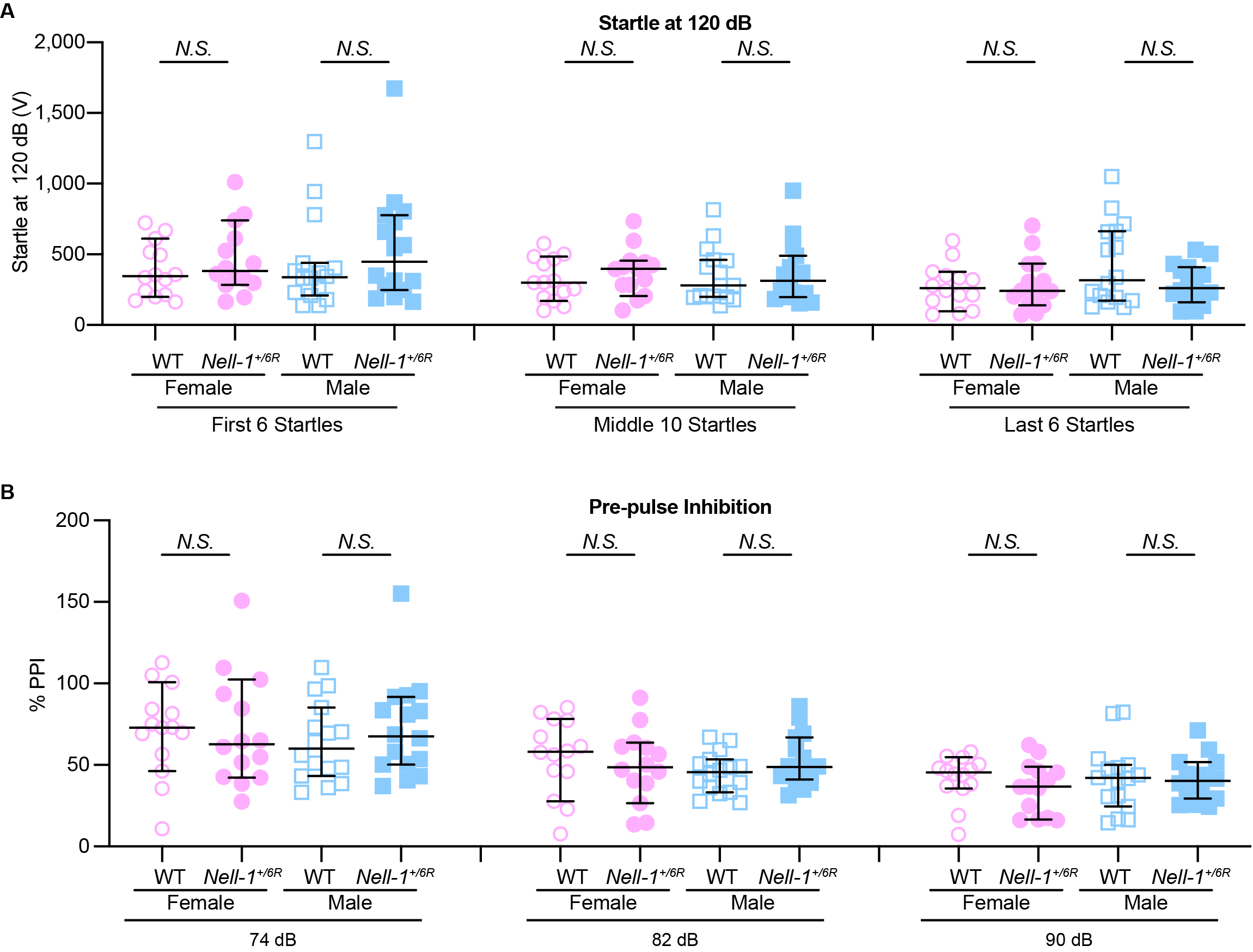


Fig. S4. The Nell-1^+/6R^ mice did not represent major changes in sensorimotor integration as indicated by the pre-pulse inhibition (PPI) test.

The mean of the first 6, middle 10, and last 6 startle at 120 dB (A) and the percentage of PPI at 74, 82, 90 dB (B) are presented. No difference was found between Nell-1^+/6R^ mice and their WT littermates for both genders. Data are presented as median ± 95% confidence interval, N = 14 (female) or 16 (male) mice per genotype, respectively. Mann-Whitney U test was used for statistical analysis. N.S.: none statistically significant.
