# Supplementary material for: Neural EGFL-like 1, a craniosynostosis-related osteochondrogenic molecule, strikingly associates with neurodevelopmental pathologies
Source: Cell Biosci. 2023 Dec 15;13:227. doi: 10.1186/s13578-023-01174-5 (PMC10725010; doi:10.1186/s13578-023-01174-5)
Supplement: Supplementary file 7 — Additional file 7: Fig. S5.The Nell-1+/6R mice did not represent major changes in motor coordination as indicated by the Rotarod performance test. (A) The length of latency to fall for 3-month-old Nell-1+/6R mice and their WT littermates, as well as (B) the revolutions per minute (rpm) for 3-month-old Nell-1+/6R mice and their WT counterparts are presented. No difference was found between Nell-1+/6R mice and their WT littermates for both genders. Data are presented as median ± interquartile range, N = 16 for each group. Mann-Whitney U test was used for statistical analysis. [file 13578_2023_1174_MOESM7_ESM.docx]

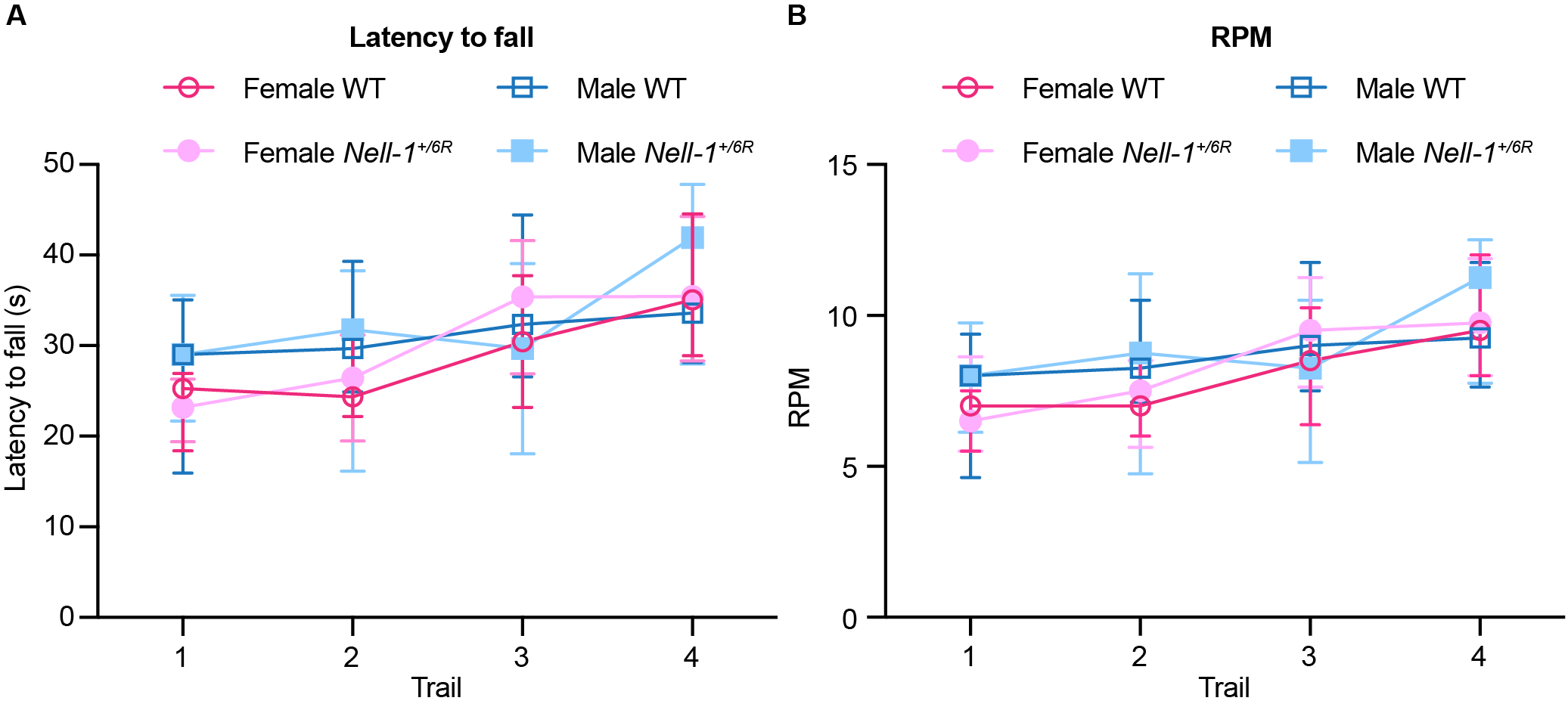


Fig. S5. The Nell-1^+/6R^ mice did not represent major changes in motor coordination as indicated by the Rotarod performance test.

(A) The length of latency to fall for 3-month-old Nell-1^+/6R^ mice and their WT littermates, as well as (B) the revolutions per minute (rpm) for 3-month-old Nell-1^+/6R^ mice and their WT counterparts are presented. No difference was found between Nell-1^+/6R^ mice and their WT littermates for both genders. Data are presented as median ± interquartile range, N = 16 for each group. Mann-Whitney U test was used for statistical analysis.
