# Supplementary material for: Neural EGFL-like 1, a craniosynostosis-related osteochondrogenic molecule, strikingly associates with neurodevelopmental pathologies
Source: Cell Biosci. 2023 Dec 15;13:227. doi: 10.1186/s13578-023-01174-5 (PMC10725010; doi:10.1186/s13578-023-01174-5)
Supplement: Supplementary file 8 — Additional file 8: Fig. S6.The Nell-1+/6R mice did not represent major changes in learning and memory as indicated by the fear conditioning test. The baseline (BL, A), percentage time of freezing behavior during the total testing time of context fear (B), tone (C), and trace (D) for both trace and delay fear conditioning tests of 3-month-old Nell-1+/6R mice and their WT littermates are presented. No difference was found between Nell-1+/6R mice and their WT counterparts for both genders. Data are presented as median ± 95% confidence interval. In the trace conditioning test, N= 16 (female) or 8 (male) for each genotype; in the delay conditioning test, N = 14 (female) or 8 (male) for each genotype, respectively. Mann-Whitney U test was used for statistical analysis. N.S.: none statistically significant. [file 13578_2023_1174_MOESM8_ESM.docx]

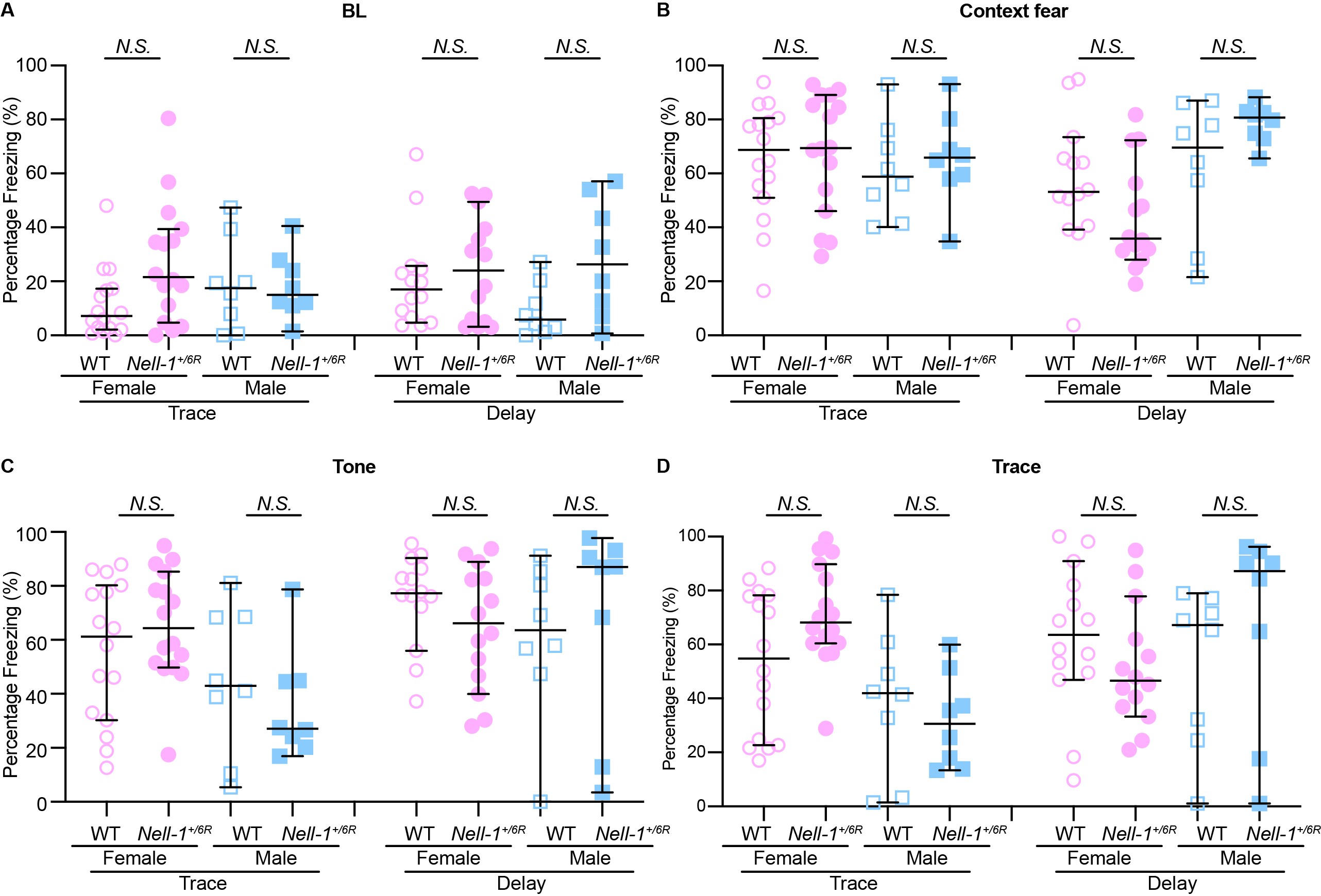


Fig. S6. The Nell-1^+/6R^ mice did not represent major changes in learning and memory as indicated by the fear conditioning test.

The baseline (BL, A), percentage time of freezing behavior during the total testing time of context fear (B), tone (C), and trace (D) for both trace and delay fear conditioning tests of 3-month-old Nell-1^+/6R^ mice and their WT littermates are presented. No difference was found between Nell-1^+/6R^ mice and their WT counterparts for both genders. Data are presented as median ± 95% confidence interval. In the trace conditioning test, N = 16 (female) or 8 (male) for each genotype; in the delay conditioning test, N = 14 (female) or 8 (male) for each genotype, respectively. Mann-Whitney U test was used for statistical analysis. N.S.: none statistically significant.
