# Supplementary material for: Neural EGFL-like 1, a craniosynostosis-related osteochondrogenic molecule, strikingly associates with neurodevelopmental pathologies
Source: Cell Biosci. 2023 Dec 15;13:227. doi: 10.1186/s13578-023-01174-5 (PMC10725010; doi:10.1186/s13578-023-01174-5)
Supplement: Supplementary file 9 — Additional file 9: Table S2.The list of differentially expressed genes (DEGs) in the hippocampus from Nell-1+/6R mice and their wild-type littermates. [file 13578_2023_1174_MOESM9_ESM.docx]

Table S2. The list of differentially expressed genes (DEGs) in the hippocampus from Nell-1^+/6R^ mice and their wild-type littermates. The DEGs were defined with a P-value less than 0.05 and log_2_FC larger than 0.58695 (aka. differential expression fold larger than 1.5) and were listed based on the log_2_FC in a descending sequence.

| **ENTREZID** | **SYMBOL** | **GENENAME** | **log_2_FC** | ***P*-Value** |
| --- | --- | --- | --- | --- |
| 75338 | *Ccdc83* | *coiled-coil domain containing 83* | 3.8551290 | 3.684E-03 |
| 15139 | *Hc* | *hemolytic complement* | 1.8030657 | 4.899E-03 |
| 108637 | *Snord14c* | *small nucleolar RNA, C/D box 14C* | 1.7887672 | 8.482E-04 |
| 100502679 | *Gm15577* | *predicted gene 15577* | 1.6774122 | 5.972E-03 |
| 72219 | *Spata31d1a* | *spermatogenesis associated 31 subfamily D, member 1A* | 1.6750267 | 3.701E-03 |
| 27218 | *Slamf1* | *signaling lymphocytic activation molecule family member 1* | 1.6103838 | 7.346E-03 |
| 20646 | *Snrpn* | *small nuclear ribonucleoprotein N* | 1.5868092 | 8.625E-06 |
| 100504496 | *Gm20255* | *predicted gene, 20255* | 1.5749315 | 2.211E-02 |
| 243407 | *Qrfprl* | *pyroglutamylated RFamide peptide receptor like* | 1.5539399 | 3.283E-02 |
| 13142 | *Dao* | *D-amino acid oxidase* | 1.4981375 | 4.434E-02 |
| 69885 | *Aunip* | *aurora kinase A and ninein interacting protein* | 1.4552902 | 2.435E-02 |
| 74288 | *Spem1* | *sperm maturation 1* | 1.4413695 | 9.106E-03 |
| 100504608 | *Eef1akmt3* | *EEF1A lysine methyltransferase 3* | 1.4349760 | 2.822E-02 |
| 723880 | *Mir540* | *microRNA 540* | 1.4015769 | 1.706E-02 |
| 16873 | *Lhx5* | *LIM homeobox protein 5* | 1.3704755 | 1.767E-02 |
| 15404 | *Hoxa7* | *homeobox A7* | 1.3399527 | 3.168E-02 |
| 16189 | *Il4* | *interleukin 4* | 1.3209521 | 1.756E-02 |
| 24115 | *Best1* | *bestrophin 1* | 1.3187539 | 3.603E-03 |
| 100502869 | *Gm19422* | *predicted gene, 19422* | 1.3090495 | 5.871E-03 |
| 666038 | *Gm7902* | *predicted gene 7902* | 1.2392662 | 8.343E-03 |
| 54352 | *Irx5* | *Iroquois homeobox 5* | 1.2150701 | 3.032E-02 |
| 100526519 | *Mir3070b* | *microRNA 3070b* | 1.2099699 | 2.971E-02 |
| 328235 | *Gm5083* | *predicted gene 5083* | 1.1714098 | 2.065E-02 |
| 100038424 | *Gm10604* | *predicted gene 10604* | 1.1518887 | 4.726E-02 |
| 12647 | *Chat* | *choline acetyltransferase* | 1.1392628 | 3.785E-02 |
| 75870 | *Tcam1* | *testicular cell adhesion molecule 1* | 1.1390258 | 1.157E-02 |
| 668090 | *Gm8971* | *predicted gene 8971* | 1.1247708 | 2.331E-03 |
| 432879 | *Kbtbd6* | *kelch repeat and BTB (POZ) domain containing 6* | 1.1189610 | 6.746E-03 |
| 21897 | *Tlr1* | *toll-like receptor 1* | 1.1101095 | 4.654E-02 |
| 19023 | *Ppef2* | *protein phosphatase, EF hand calcium-binding domain 2* | 1.0992688 | 3.996E-03 |
| 17341 | *Bhlha15* | *basic helix-loop-helix family, member a15* | 1.0749697 | 1.448E-02 |
| 73246 | *Rassf6* | *Ras association (RalGDS/AF-6) domain family member 6* | 1.0493779 | 1.881E-02 |
| 328795 | *Ubash3a* | *ubiquitin associated and SH3 domain containing, A* | 1.0400357 | 2.300E-02 |
| 98365 | *Slamf9* | *SLAM family member 9* | 1.0395402 | 8.323E-03 |
| 78052 | *Tmem190* | *transmembrane protein 190* | 1.0340593 | 3.332E-02 |
| 73906 | *4833417C18Rik* | *RIKEN cDNA 4833417C18 gene* | 1.0128326 | 4.923E-02 |
| 382686 | *3110053B16Rik* | *RIKEN cDNA 3110053B16 gene* | 1.0029576 | 4.087E-02 |
| 70040 | *2610037D02Rik* | *RIKEN cDNA 2610037D02 gene* | 0.9889003 | 4.310E-02 |
| 545062 | *Gm5802* | *predicted gene 5802* | 0.9773730 | 4.341E-11 |
| 12057 | *Opn1sw* | *opsin 1 (cone pigments), short-wave-sensitive (color blindness, tritan)* | 0.9696615 | 4.662E-02 |
| 320054 | *9230116N13Rik* | *RIKEN cDNA 9230116N13 gene* | 0.9656973 | 6.831E-03 |
| 27210 | *Snord34* | *small nucleolar RNA, C/D box 34* | 0.9439432 | 2.259E-02 |
| 20277 | *Scnn1b* | *sodium channel, nonvoltage-gated 1 beta* | 0.9358639 | 3.021E-02 |
| 634650 | *Gbp11* | *guanylate binding protein 11* | 0.9222937 | 2.789E-02 |
| 75604 | *Tm4sf5* | *transmembrane 4 superfamily member 5* | 0.8914883 | 4.366E-02 |
| 100503120 | *A930006K02Rik* | *RIKEN cDNA A930006K02 gene* | 0.8834181 | 1.008E-02 |
| 627872 | *Dnah7a* | *dynein, axonemal, heavy chain 7A* | 0.8689444 | 3.231E-02 |
| 237749 | *Gm4926* | *T-cell immunoglobulin and mucin domain containing 2 pseudogene* | 0.8621031 | 3.465E-02 |
| 19944 | *Rpl29* | *ribosomal protein L29* | 0.8564394 | 4.436E-02 |
| 619293 | *9230009I02Rik* | *RIKEN cDNA 9230009I02 gene* | 0.8379776 | 1.386E-02 |
| 74574 | *Lvrn* | *laeverin* | 0.8327765 | 1.906E-02 |
| 17984 | *Ndn* | *necdin, MAGE family member* | 0.7916275 | 9.285E-08 |
| 67531 | *5730408K05Rik* | *RIKEN cDNA 5730408K05 gene* | 0.7908405 | 4.111E-02 |
| 70258 | *1500035N22Rik* | *RIKEN cDNA 1500035N22 gene* | 0.7870456 | 3.998E-02 |
| 23834 | *Cdc6* | *cell division cycle 6* | 0.7813636 | 4.280E-02 |
| 215384 | *Fcgbp* | *Fc fragment of IgG binding protein* | 0.7708757 | 4.313E-02 |
| 16371 | *Irx1* | *Iroquois homeobox 1* | 0.7621869 | 1.125E-02 |
| 64833 | *Acot10* | *acyl-CoA thioesterase 10* | 0.7476703 | 1.359E-02 |
| 73673 | *Rec114* | *REC114 meiotic recombination protein* | 0.7354381 | 4.262E-02 |
| 76652 | *Actrt3* | *actin related protein T3* | 0.7247986 | 1.311E-02 |
| 22293 | *Slc45a2* | *solute carrier family 45, member 2* | 0.7035513 | 3.929E-02 |
| 78625 | *Acsbg3* | *acyl-CoA synthetase bubblegum family member 3* | 0.6918986 | 1.383E-02 |
| 50540 | *Igbp1b* | *immunoglobulin (CD79A) binding protein 1b* | 0.6747519 | 4.580E-02 |
| 100048926 | *Gm12945* | *predicted gene 12945* | 0.6720459 | 3.474E-02 |
| 20135 | *Rrm2* | *ribonucleotide reductase M2* | 0.6441455 | 2.189E-02 |
| 100038688 | *Gm10724* | *predicted gene 10724* | 0.6399071 | 8.341E-04 |
| 329702 | *Dcst2* | *DC-STAMP domain containing 2* | 0.6175406 | 3.515E-02 |
| 269513 | *Nkain3* | *Na+/K+ transporting ATPase interacting 3* | 0.6060493 | 2.230E-02 |
| 268816 | *Mroh5* | *maestro heat-like repeat family member 5* | 0.6012332 | 4.127E-02 |
| 16797 | *Lat* | *linker for activation of T cells* | -0.5870149 | 3.263E-02 |
| 26570 | *Slc7a11* | *solute carrier family 7 (cationic amino acid transporter, y+ system), member 11* | -0.5922999 | 4.084E-02 |
| 12162 | *Bmp7* | *bone morphogenetic protein 7* | -0.5952600 | 4.174E-02 |
| 140792 | *Colec12* | *collectin sub-family member 12* | -0.5970642 | 2.012E-02 |
| 50905 | *Il17rb* | *interleukin 17 receptor B* | -0.5975671 | 1.222E-02 |
| 18605 | *Enpp1* | *ectonucleotide pyrophosphatase/phosphodiesterase 1* | -0.6028829 | 4.725E-02 |
| 75516 | *Ttc32* | *tetratricopeptide repeat domain 32* | -0.6067214 | 4.114E-02 |
| 383619 | *Aim2* | *absent in melanoma 2* | -0.6084505 | 3.360E-02 |
| 227157 | *Mpp4* | *membrane protein, palmitoylated 4 (MAGUK p55 subfamily member 4)* | -0.6131866 | 4.730E-02 |
| 17022 | *Lum* | *lumican* | -0.6140781 | 2.881E-02 |
| 13717 | *Eln* | *elastin* | -0.6153204 | 5.152E-04 |
| 17130 | *Smad6* | *SMAD family member 6* | -0.6171082 | 3.747E-02 |
| 20305 | *Ccl6* | *chemokine (C-C motif) ligand 6* | -0.6185628 | 2.397E-02 |
| 50908 | *C1s1* | *complement component 1, s subcomponent 1* | -0.6207155 | 2.572E-02 |
| 319752 | *B230209E15Rik* | *RIKEN cDNA B230209E15 gene* | -0.6442846 | 9.242E-04 |
| 12870 | *Cp* | *ceruloplasmin* | -0.6531650 | 1.828E-02 |
| 12840 | *Col9a2* | *collagen, type IX, alpha 2* | -0.6566594 | 2.425E-02 |
| 320026 | *A330076H08Rik* | *RIKEN cDNA A330076H08 gene* | -0.6682786 | 1.026E-02 |
| 22371 | *Vwf* | *Von Willebrand factor* | -0.6784132 | 1.746E-03 |
| 56050 | *Cyp39a1* | *cytochrome P450, family 39, subfamily a, polypeptide 1* | -0.6793807 | 1.068E-02 |
| 76365 | *Tbx18* | *T-box18* | -0.6819091 | 2.180E-02 |
| 93694 | *Clec2d* | *C-type lectin domain family 2, member d* | -0.6825173 | 3.161E-02 |
| 68713 | *Ifitm1* | *interferon induced transmembrane protein 1* | -0.6872124 | 3.491E-02 |
| 12161 | *Bmp6* | *bone morphogenetic protein 6* | -0.6876947 | 2.575E-02 |
| 13614 | *Edn1* | *endothelin 1* | -0.6974583 | 6.784E-03 |
| 434215 | *Lrrc32* | *leucine rich repeat containing 32* | -0.6990946 | 8.222E-04 |
| 19016 | *Pparg* | *peroxisome proliferator activated receptor gamma* | -0.7013788 | 2.593E-03 |
| 546336 | *Prrg1* | *proline rich Gla (G-carboxyglutamic acid) 1* | -0.7084232 | 2.569E-02 |
| 233328 | *Lrrk1* | *leucine-rich repeat kinase 1* | -0.7106452 | 1.023E-03 |
| 17534 | *Mrc2* | *mannose receptor, C type 2* | -0.7112152 | 1.245E-02 |
| 19265 | *Ptprcap* | *protein tyrosine phosphatase, receptor type, C polypeptide-associated protein* | -0.7120700 | 4.970E-02 |
| 23956 | *Neu2* | *neuraminidase 2* | -0.7144198 | 4.093E-02 |
| 170720 | *Card14* | *caspase recruitment domain family, member 14* | -0.7160784 | 3.047E-02 |
| 71130 | *Sh2d6* | *SH2 domain containing 6* | -0.7180910 | 4.362E-02 |
| 107885 | *Mthfs* | *5, 10-methenyltetrahydrofolate synthetase* | -0.7185018 | 2.533E-02 |
| 110935 | *Atp6v1b1* | *ATPase, H+ transporting, lysosomal V1 subunit B1* | -0.7195448 | 4.177E-02 |
| 17921 | *Myo7a* | *myosin VIIA* | -0.7207700 | 4.737E-04 |
| 12159 | *Bmp4* | *bone morphogenetic protein 4* | -0.7234978 | 1.886E-02 |
| 17313 | *Mgp* | *matrix Gla protein* | -0.7434796 | 4.307E-02 |
| 27493 | *A230006K03Rik* | *RIKEN cDNA A230006K03 gene* | -0.7560579 | 2.062E-02 |
| 268527 | *Greb1* | *gene regulated by estrogen in breast cancer protein* | -0.7679003 | 3.612E-02 |
| 13346 | *Des* | *desmin* | -0.7713963 | 8.030E-03 |
| 233246 | *Ano5* | *anoctamin 5* | -0.7766972 | 9.260E-03 |
| 214642 | *Cped1* | *cadherin-like and PC-esterase domain containing 1* | -0.7780973 | 4.360E-03 |
| 100503166 | *1700048M11Rik* | *RIKEN cDNA 1700048M11 gene* | -0.7840861 | 3.816E-02 |
| 207818 | *Smagp* | *small cell adhesion glycoprotein* | -0.7845182 | 1.289E-02 |
| 387204 | *Mir208a* | *microRNA 208a* | -0.7865052 | 4.336E-02 |
| 12258 | *Serping1* | *serine (or cysteine) peptidase inhibitor, clade G, member 1* | -0.7981352 | 6.115E-03 |
| 15229 | *Foxd1* | *forkhead box D1* | -0.7997652 | 4.061E-02 |
| 21345 | *Tagln* | *transgelin* | -0.8019400 | 1.484E-02 |
| 11536 | *Gpr182* | *G protein-coupled receptor 182* | -0.8059026 | 2.876E-02 |
| 71934 | *Car13* | *carbonic anhydrase 13* | -0.8169121 | 1.849E-02 |
| 77036 | *1700109H08Rik* | *RIKEN cDNA 1700109H08 gene* | -0.8185658 | 4.102E-02 |
| 100862268 | *Gm16958* | *predicted gene, 16958* | -0.8208368 | 2.660E-02 |
| 629147 | *Ctxn3* | *cortexin 3* | -0.8328177 | 2.369E-02 |
| 23876 | *Fbln5* | *fibulin 5* | -0.8462602 | 6.480E-03 |
| 102680 | *Slc6a20a* | *solute carrier family 6 (neurotransmitter transporter), member 20A* | -0.8481846 | 3.183E-02 |
| 16402 | *Itga5* | *integrin alpha 5 (fibronectin receptor alpha)* | -0.8521690 | 2.114E-03 |
| 14181 | *Fgfbp1* | *fibroblast growth factor binding protein 1* | -0.8720994 | 4.253E-02 |
| 78906 | *Misp* | *mitotic spindle positioning* | -0.8745668 | 2.545E-02 |
| 170786 | *Cd209a* | *CD209a antigen* | -0.8798783 | 2.585E-02 |
| 27384 | *Akr1c13* | *aldo-keto reductase family 1, member C13* | -0.8800378 | 3.162E-02 |
| 13078 | *Cyp1b1* | *cytochrome P450, family 1, subfamily b, polypeptide 1* | -0.8801496 | 4.227E-03 |
| 215798 | *Adgrg6* | *adhesion G protein-coupled receptor G6* | -0.8804066 | 1.623E-02 |
| 18821 | *Pln* | *phospholamban* | -0.8818011 | 1.202E-02 |
| 791281 | *Gm10253* | *predicted gene 10253* | -0.8900747 | 3.359E-02 |
| 12842 | *Col1a1* | *collagen, type I, alpha 1* | -0.8941301 | 1.015E-02 |
| 17880 | *Myh11* | *myosin, heavy polypeptide 11, smooth muscle* | -0.8964312 | 1.957E-02 |
| 93671 | *Cd163* | *CD163 antigen* | -0.9026488 | 4.723E-03 |
| 71724 | *Aox3* | *aldehyde oxidase 3* | -0.9056018 | 6.745E-03 |
| 114332 | *Lyve1* | *lymphatic vessel endothelial hyaluronan receptor 1* | -0.9121963 | 1.688E-02 |
| 56847 | *Aldh1a3* | *aldehyde dehydrogenase family 1, subfamily A3* | -0.9136753 | 1.139E-02 |
| 320110 | *B230369F24Rik* | *RIKEN cDNA B230369F24 gene* | -0.9140707 | 2.651E-02 |
| 241327 | *Olfml2a* | *olfactomedin-like 2A* | -0.9155444 | 1.419E-02 |
| 74959 | *Platr14* | *pluripotency associated transcript 14* | -0.9201024 | 3.431E-02 |
| 100216343 | *Gm17501* | *predicted gene, 17501* | -0.9330451 | 3.400E-02 |
| 20519 | *Slc22a3* | *solute carrier family 22 (organic cation transporter), member 3* | -0.9364944 | 1.558E-03 |
| 338352 | *Nell1* | *NEL-like 1* | -0.9366979 | 2.400E-08 |
| 353025 | *Caps2* | *calcyphosphine 2* | -0.9404326 | 2.260E-02 |
| 100503922 | *Gm19967* | *predicted gene, 19967* | -0.9415536 | 3.064E-02 |
| 242653 | *Cldn19* | *claudin 19* | -0.9434310 | 3.036E-02 |
| 11754 | *Aoc3* | *amine oxidase, copper containing 3* | -0.9460987 | 1.881E-02 |
| 12810 | *Coch* | *cochlin* | -0.9550197 | 3.120E-02 |
| 226040 | *Tmem252* | *transmembrane protein 252* | -0.9619538 | 1.564E-02 |
| 17533 | *Mrc1* | *mannose receptor, C type 1* | -0.9724292 | 2.446E-03 |
| 100038470 | *Gm10808* | *predicted gene 10808* | -0.9767281 | 3.465E-02 |
| 12409 | *Cbr2* | *carbonyl reductase 2* | -0.9881865 | 1.636E-02 |
| 12904 | *Crabp2* | *cellular retinoic acid binding protein II* | -0.9900160 | 3.527E-02 |
| 100038541 | *Gm10554* | *predicted gene 10554* | -0.9933264 | 1.377E-02 |
| 74364 | *4931431C16Rik* | *RIKEN cDNA 4931431C16 gene* | -0.9941288 | 2.117E-02 |
| 16425 | *Itih2* | *inter-alpha trypsin inhibitor, heavy chain 2* | -1.0093898 | 2.390E-02 |
| 14619 | *Gjb2* | *gap junction protein, beta 2* | -1.0311473 | 1.797E-02 |
| 381319 | *Batf3* | *basic leucine zipper transcription factor, ATF-like 3* | -1.0339468 | 2.874E-02 |
| 209387 | *Trim30d* | *tripartite motif-containing 30D* | -1.0481942 | 3.641E-02 |
| 668929 | *Rad21l* | *RAD21-like (S. pombe)* | -1.0489415 | 1.913E-02 |
| 75558 | *Spata45* | *spermatogenesis associated 45* | -1.0499913 | 4.268E-02 |
| 434198 | *B130024G19Rik* | *RIKEN cDNA B130024G19 gene* | -1.0522545 | 2.543E-02 |
| 11475 | *Acta2* | *actin, alpha 2, smooth muscle, aorta* | -1.0626510 | 1.381E-02 |
| 414089 | *Gja6* | *gap junction protein, alpha 6* | -1.0652114 | 4.043E-02 |
| 73677 | *Psma8* | *proteasome subunit alpha 8* | -1.0760516 | 4.408E-02 |
| 72104 | *2010106C02Rik* | *RIKEN cDNA 2010106C02 gene* | -1.0772048 | 3.724E-02 |
| 69797 | *1600029I14Rik* | *RIKEN cDNA 1600029I14 gene* | -1.0772377 | 1.712E-02 |
| 22599 | *Slc6a20b* | *solute carrier family 6 (neurotransmitter transporter), member 20B* | -1.0895430 | 2.034E-02 |
| 637008 | *Gm11793* | *predicted gene 11793* | -1.1031452 | 2.999E-02 |
| 225004 | *Pcare* | *photoreceptor cilium actin regulator* | -1.1191511 | 1.665E-02 |
| 20533 | *Slc4a1* | *solute carrier family 4 (anion exchanger), member 1* | -1.1208571 | 1.267E-02 |
| 76681 | *Trim12a* | *tripartite motif-containing 12A* | -1.1212474 | 2.028E-03 |
| 100217455 | *Snord49a* | *small nucleolar RNA, C/D box 49A* | -1.1229557 | 3.336E-02 |
| 100039139 | *Ccdc152* | *coiled-coil domain containing 152* | -1.1259756 | 4.487E-02 |
| 12843 | *Col1a2* | *collagen, type I, alpha 2* | -1.1276031 | 8.574E-03 |
| 208164 | *Fam180a* | *family with sequence similarity 180, member A* | -1.1279392 | 3.805E-02 |
| 387133 | *Mir9-1* | *microRNA 9-1* | -1.1280331 | 2.883E-03 |
| 74568 | *Mlkl* | *mixed lineage kinase domain-like* | -1.1333394 | 3.851E-02 |
| 319893 | *A230057D06Rik* | *RIKEN cDNA A230057D06 gene* | -1.1404874 | 8.225E-06 |
| 11694 | *Alx3* | *aristaless-like homeobox 3* | -1.1431174 | 1.704E-02 |
| 11695 | *Alx4* | *aristaless-like homeobox 4* | -1.1443919 | 1.222E-02 |
| 280635 | *Emilin3* | *elastin microfibril interfacer 3* | -1.1548987 | 3.619E-02 |
| 333669 | *Gm5134* | *predicted gene 5134* | -1.1808951 | 4.941E-02 |
| 12825 | *Col3a1* | *collagen, type III, alpha 1* | -1.1887206 | 2.245E-03 |
| 22445 | *Xlr3a* | *X-linked lymphocyte-regulated 3A* | -1.1906591 | 3.093E-02 |
| 20342 | *Selenbp2* | *selenium binding protein 2* | -1.1909903 | 2.590E-02 |
| 67342 | *Kcnmb4os1* | *potassium large conductance calcium-activated channel, subfamily M, beta member 4, opposite strand 1* | -1.2015926 | 1.503E-02 |
| 13011 | *Cst7* | *cystatin F (leukocystatin)* | -1.2028717 | 3.154E-02 |
| 22403 | *Ccn5* | *cellular communication network factor 5* | -1.2056859 | 4.679E-02 |
| 18779 | *Pla2r1* | *phospholipase A2 receptor 1* | -1.2191899 | 4.607E-03 |
| 64082 | *Popdc2* | *popeye domain containing 2* | -1.2342965 | 4.853E-02 |
| 56744 | *Pf4* | *platelet factor 4* | -1.2424535 | 7.424E-03 |
| 278725 | *E130310I04Rik* | *RIKEN cDNA E130310I04 gene* | -1.2430799 | 3.698E-02 |
| 17240 | *Mdfi* | *MyoD family inhibitor* | -1.2448098 | 2.239E-02 |
| 140919 | *Slc17a6* | *solute carrier family 17 (sodium-dependent inorganic phosphate cotransporter), member 6* | -1.2538141 | 2.265E-07 |
| 268973 | *Nlrc4* | *NLR family, CARD domain containing 4* | -1.2548013 | 4.886E-02 |
| 98870 | *AI182371* | *expressed sequence AI182371* | -1.2558491 | 4.676E-02 |
| 14412 | *Slc6a13* | *solute carrier family 6 (neurotransmitter transporter, GABA), member 13* | -1.2592623 | 1.334E-02 |
| 278507 | *Wfikkn2* | *WAP, follistatin/kazal, immunoglobulin, kunitz and netrin domain containing 2* | -1.2679029 | 3.036E-02 |
| 20612 | *Siglec1* | *sialic acid binding Ig-like lectin 1, sialoadhesin* | -1.2764397 | 1.249E-02 |
| 100503873 | *Gm19937* | *predicted gene, 19937* | -1.2820770 | 1.848E-02 |
| 193286 | *BC049762* | *cDNA sequence BC049762* | -1.2931910 | 3.009E-02 |
| 15129 | *Hbb-b1* | *hemoglobin, beta adult major chain* | -1.2952211 | 1.481E-02 |
| 12363 | *Casp4* | *caspase 4, apoptosis-related cysteine peptidase* | -1.2959947 | 3.786E-02 |
| 73921 | *Scpep1os* | *serine carboxypeptidase 1, opposite strand* | -1.2962452 | 3.418E-02 |
| 270624 | *Spin4* | *spindlin family, member 4* | -1.3018229 | 4.509E-02 |
| 14012 | *Mpzl2* | *myelin protein zero-like 2* | -1.3123001 | 2.101E-02 |
| 19214 | *Ptgdr* | *prostaglandin D receptor* | -1.3163872 | 2.232E-02 |
| 109163 | *3010003L21Rik* | *RIKEN cDNA 3010003L21 gene* | -1.3235796 | 1.871E-02 |
| 668415 | *LOC668415* | *uncharacterized LOC668415* | -1.3322762 | 3.112E-02 |
| 14961 | *H2-Ab1* | *histocompatibility 2, class II antigen A, beta 1* | -1.3327730 | 9.331E-03 |
| 170484 | *Nphs2* | *nephrosis 2, podocin* | -1.3367826 | 3.854E-02 |
| 269902 | *Vmn2r57* | *vomeronasal 2, receptor 57* | -1.3519311 | 4.436E-02 |
| 213945 | *Col28a1* | *collagen, type XXVIII, alpha 1* | -1.3651746 | 2.887E-03 |
| 19118 | *Prm1* | *protamine 1* | -1.3672623 | 1.976E-02 |
| 319154 | *H3c13* | *H3 clustered histone 13* | -1.3725275 | 2.430E-02 |
| 76615 | *Got1l1* | *glutamic-oxaloacetic transaminase 1-like 1* | -1.3790211 | 3.641E-02 |
| 14411 | *Slc6a12* | *solute carrier family 6 (neurotransmitter transporter, betaine/GABA), member 12* | -1.3912834 | 3.059E-02 |
| 20495 | *Slc12a1* | *solute carrier family 12, member 1* | -1.4288368 | 1.953E-02 |
| 27052 | *Aoah* | *acyloxyacyl hydrolase* | -1.4327305 | 1.724E-02 |
| 100502750 | *Gm19351* | *predicted gene, 19351* | -1.4416950 | 1.065E-02 |
| 16196 | *Il7* | *interleukin 7* | -1.4490633 | 1.851E-02 |
| 14264 | *Fmod* | *fibromodulin* | -1.4587741 | 4.277E-02 |
| 219132 | *Phf11d* | *PHD finger protein 11D* | -1.4667556 | 1.794E-02 |
| 100038708 | *Gm10855* | *predicted gene 10855* | -1.4676792 | 1.568E-02 |
| 14234 | *Foxc2* | *forkhead box C2* | -1.4690191 | 4.630E-02 |
| 18399 | *Slc22a6* | *solute carrier family 22 (organic anion transporter), member 6* | -1.4832766 | 1.666E-02 |
| 18985 | *Pou2af1* | *POU domain, class 2, associating factor 1* | -1.4917920 | 4.403E-02 |
| 628693 | *Phf11* | *PHD finger protein 11* | -1.4999063 | 2.687E-02 |
| 71690 | *Esm1* | *endothelial cell-specific molecule 1* | -1.5036128 | 2.393E-02 |
| 271639 | *Adcy10* | *adenylate cyclase 10* | -1.5255185 | 1.388E-03 |
| 243755 | *Slc13a4* | *solute carrier family 13 (sodium/sulfate symporters), member 4* | -1.5351991 | 4.812E-02 |
| 667947 | *Gm12247* | *predicted gene 12247* | -1.5469800 | 8.691E-03 |
| 217593 | *Slc25a21* | *solute carrier family 25 (mitochondrial oxodicarboxylate carrier), member 21* | -1.5476460 | 7.024E-03 |
| 20508 | *Slc18a3* | *solute carrier family 18 (vesicular monoamine), member 3* | -1.5625500 | 2.412E-02 |
| 29818 | *Hspb7* | *heat shock protein family, member 7 (cardiovascular)* | -1.5656055 | 4.194E-02 |
| 218624 | *Il31ra* | *interleukin 31 receptor A* | -1.5757494 | 2.971E-02 |
| 15109 | *Hal* | *histidine ammonia lyase* | -1.5781291 | 1.082E-02 |
| 100043600 | *Gm4544* | *predicted gene 4544* | -1.5811527 | 8.932E-03 |
| 52024 | *Ankrd22* | *ankyrin repeat domain 22* | -1.5994816 | 3.769E-02 |
| 12550 | *Cdh1* | *cadherin 1* | -1.6138063 | 4.324E-02 |
| 72121 | *Dennd2d* | *DENN/MADD domain containing 2D* | -1.6140949 | 8.791E-03 |
| 13393 | *Dlx3* | *distal-less homeobox 3* | -1.6386856 | 2.959E-02 |
| 19378 | *Aldh1a2* | *aldehyde dehydrogenase family 1, subfamily A2* | -1.6537069 | 6.930E-03 |
| 100038693 | *Tmem51os1* | *Tmem51 opposite strand 1* | -1.6565599 | 6.111E-03 |
| 12772 | *Ccr2* | *chemokine (C-C motif) receptor 2* | -1.6987672 | 8.444E-03 |
| 16353 | *Ipw* | *imprinted gene in the Prader-Willi syndrome region* | -1.7188525 | 1.018E-07 |
| 105590 | *Zfp957* | *zinc finger protein 957* | -1.7247306 | 7.207E-04 |
| 103149 | *Upb1* | *ureidopropionase, beta* | -1.7316287 | 2.648E-03 |
| 17301 | *Foxd2* | *forkhead box D2* | -1.7499337 | 8.884E-03 |
| 232413 | *Clec12a* | *C-type lectin domain family 12, member a* | -1.7529876 | 4.195E-03 |
| 403395 | *Clec3a* | *C-type lectin domain family 3, member a* | -1.8379861 | 7.454E-03 |
| 269053 | *Gpr152* | *G protein-coupled receptor 152* | -1.8465457 | 2.231E-02 |
| 319192 | *H2ac19* | *H2A clustered histone 19* | -1.8757007 | 2.100E-03 |
| 17228 | *Cma1* | *chymase 1, mast cell* | -1.8861344 | 1.839E-02 |
| 18096 | *Nkx6-1* | *NK6 homeobox 1* | -1.9560441 | 4.101E-02 |
| 12873 | *Cpa3* | *carboxypeptidase A3, mast cell* | -1.9568549 | 2.243E-02 |
| 19331 | *Rab19* | *RAB19, member RAS oncogene family* | -1.9640467 | 4.297E-03 |
| 22420 | *Wnt6* | *wingless-type MMTV integration site family, member 6* | -1.9655968 | 1.633E-02 |
| 16948 | *Lox* | *lysyl oxidase* | -2.0291262 | 9.124E-03 |
| 11811 | *Apobec2* | *apolipoprotein B mRNA editing enzyme, catalytic polypeptide 2* | -2.1352367 | 3.880E-04 |
| 76432 | *2310001H17Rik* | *RIKEN cDNA 2310001H17 gene* | -2.2214104 | 7.256E-04 |
| 17227 | *Mcpt4* | *mast cell protease 4* | -2.4325893 | 3.827E-03 |
| 17229 | *Tpsb2* | *tryptase beta 2* | -2.4590065 | 1.224E-03 |
| 107626 | *Asmt* | *acetylserotonin O-methyltransferase* | -2.7272617 | 2.384E-04 |
