# Supplementary material for: Neural EGFL-like 1, a craniosynostosis-related osteochondrogenic molecule, strikingly associates with neurodevelopmental pathologies
Source: Cell Biosci. 2023 Dec 15;13:227. doi: 10.1186/s13578-023-01174-5 (PMC10725010; doi:10.1186/s13578-023-01174-5)
Supplement: Supplementary file 10 — Additional file 10: Table S3.The converting results of the input downregulated DEGs in the Metascape. [file 13578_2023_1174_MOESM10_ESM.docx]

Table S3. The converting results of the input downregulated DEGs in the Metascape. The DEGs were converted into their corresponding H. sapiens Entrez gene IDs using the latest version of the database (last updated on 2021-08-01). If multiple identifiers correspond to the same Entrez gene ID, they were considered as a single Entrez gene ID in downstream analyses.

| **MyList** | **Gene ID** | **Type** | **Tax ID** | **Homologene Gene ID** | **Homologene Gene Tax ID** | **Gene Symbol** | **Description** |
| --- | --- | --- | --- | --- | --- | --- | --- |
| *Lat* | 16797 | symbol | *M. musculus* | 27040 | *H. sapiens* | *LAT* | *linker for activation of T cells* |
| *Slc7a11* | 26570 | symbol | *M. musculus* | 23657 | *H. sapiens* | *SLC7A11* | *solute carrier family 7 member 11* |
| *Bmp7* | 12162 | symbol | *M. musculus* | 655 | *H. sapiens* | *BMP7* | *bone morphogenetic protein 7* |
| *Colec12* | 140792 | symbol | *M. musculus* | 81035 | *H. sapiens* | *COLEC12* | *collectin subfamily member 12* |
| *Il17rb* | 50905 | symbol | *M. musculus* | 55540 | *H. sapiens* | *IL17RB* | *interleukin 17 receptor B* |
| *Enpp1* | 18605 | symbol | *M. musculus* | 5167 | *H. sapiens* | *ENPP1* | *ectonucleotide pyrophosphatase/phosphodiesterase 1* |
| *Ttc32* | 75516 | symbol | *M. musculus* | 130502 | *H. sapiens* | *TTC32* | *tetratricopeptide repeat domain 32* |
| *Aim2* | 383619 | symbol | *M. musculus* | 9447 | *H. sapiens* | *AIM2* | *absent in melanoma 2* |
| *Mpp4* | 227157 | symbol | *M. musculus* | 58538 | *H. sapiens* | *MPP4* | *membrane palmitoylated protein 4* |
| *Lum* | 17022 | symbol | *M. musculus* | 4060 | *H. sapiens* | *LUM* | *lumican* |
| *Eln* | 13717 | symbol | *M. musculus* | 2006 | *H. sapiens* | *ELN* | *elastin* |
| *Smad6* | 17130 | symbol | *M. musculus* | 4091 | *H. sapiens* | *SMAD6* | *SMAD family member 6* |
| *Ccl6* | 20305 | symbol | *M. musculus* | 6359 | *H. sapiens* | *CCL15* | *C-C motif chemokine ligand 15* |
| *C1s1* | 50908 | symbol | *M. musculus* | 716 | *H. sapiens* | *C1S* | *complement C1s* |
| *B230209E15Rik* | 319752 | symbol | *M. musculus* |  |  | None | None |
| *Cp* | 12870 | symbol | *M. musculus* | 1356 | *H. sapiens* | *CP* | *ceruloplasmin* |
| *Col9a2* | 12840 | symbol | *M. musculus* | 1298 | *H. sapiens* | *COL9A2* | *collagen type IX alpha 2 chain* |
| *A330076H08Rik* | 320026 | symbol | *M. musculus* |  |  | None | None |
| *Vwf* | 22371 | symbol | *M. musculus* | 7450 | *H. sapiens* | *VWF* | *von Willebrand factor* |
| *Cyp39a1* | 56050 | symbol | *M. musculus* | 51302 | *H. sapiens* | *CYP39A1* | *cytochrome P450 family 39 subfamily A member 1* |
| *Tbx18* | 76365 | symbol | *M. musculus* | 9096 | *H. sapiens* | *TBX18* | *T-box transcription factor 18* |
| *Clec2d* | 93694 | symbol | *M. musculus* | 29121 | *H. sapiens* | *CLEC2D* | *C-type lectin domain family 2 member D* |
| *Ifitm1* | 68713 | symbol | *M. musculus* | 10410 | *H. sapiens* | *IFITM3* | *interferon induced transmembrane protein 3* |
| *Bmp6* | 12161 | symbol | *M. musculus* | 654 | *H. sapiens* | *BMP6* | *bone morphogenetic protein 6* |
| *Edn1* | 13614 | symbol | *M. musculus* | 1906 | *H. sapiens* | *EDN1* | *endothelin 1* |
| *Lrrc32* | 434215 | symbol | *M. musculus* | 2615 | *H. sapiens* | *LRRC32* | *leucine rich repeat containing 32* |
| *Pparg* | 19016 | symbol | *M. musculus* | 5468 | *H. sapiens* | *PPARG* | *peroxisome proliferator activated receptor gamma* |
| *Prrg1* | 546336 | symbol | *M. musculus* | 5638 | *H. sapiens* | *PRRG1* | *proline rich and Gla domain 1* |
| *Lrrk1* | 233328 | symbol | *M. musculus* | 79705 | *H. sapiens* | *LRRK1* | *leucine rich repeat kinase 1* |
| *Mrc2* | 17534 | symbol | *M. musculus* | 9902 | *H. sapiens* | *MRC2* | *mannose receptor C type 2* |
| *Ptprcap* | 19265 | symbol | *M. musculus* | 5790 | *H. sapiens* | *PTPRCAP* | *protein tyrosine phosphatase receptor type C associated protein* |
| *Neu2* | 23956 | symbol | *M. musculus* | 4759 | *H. sapiens* | *NEU2* | *neuraminidase 2* |
| *Card14* | 170720 | symbol | *M. musculus* | 79092 | *H. sapiens* | *CARD14* | *caspase recruitment domain family member 14* |
| *Sh2d6* | 71130 | symbol | *M. musculus* | 284948 | *H. sapiens* | *SH2D6* | *SH2 domain containing 6* |
| *Mthfs* | 107885 | symbol | *M. musculus* | 10588 | *H. sapiens* | *MTHFS* | *methenyltetrahydrofolate synthetase* |
| *Atp6v1b1* | 110935 | symbol | *M. musculus* | 525 | *H. sapiens* | *ATP6V1B1* | *ATPase H+ transporting V1 subunit B1* |
| *Myo7a* | 17921 | symbol | *M. musculus* | 4647 | *H. sapiens* | *MYO7A* | *myosin VIIA* |
| *Bmp4* | 12159 | symbol | *M. musculus* | 652 | *H. sapiens* | *BMP4* | *bone morphogenetic protein 4* |
| *Mgp* | 17313 | symbol | *M. musculus* | 4256 | *H. sapiens* | *MGP* | *matrix Gla protein* |
| *A230006K03Rik* | 27493 | symbol | *M. musculus* |  |  | None | None |
| *Greb1* | 268527 | symbol | *M. musculus* | 9687 | *H. sapiens* | *GREB1* | *growth regulating estrogen receptor binding 1* |
| *Des* | 13346 | symbol | *M. musculus* | 1674 | *H. sapiens* | *DES* | *desmin* |
| *Ano5* | 233246 | symbol | *M. musculus* | 203859 | *H. sapiens* | *ANO5* | *anoctamin 5* |
| *Cped1* | 214642 | symbol | *M. musculus* | 79974 | *H. sapiens* | *CPED1* | *cadherin like and PC-esterase domain containing 1* |
| *1700048M11Rik* | 100503166 | symbol | *M. musculus* |  |  | None | None |
| *Smagp* | 207818 | symbol | *M. musculus* | 57228 | *H. sapiens* | *SMAGP* | *small cell adhesion glycoprotein* |
| *Mir208a* | 387204 | symbol | *M. musculus* |  |  | None | None |
| *Serping1* | 12258 | symbol | *M. musculus* | 710 | *H. sapiens* | *SERPING1* | *serpin family G member 1* |
| *Foxd1* | 15229 | symbol | *M. musculus* | 2297 | *H. sapiens* | *FOXD1* | *forkhead box D1* |
| *Tagln* | 21345 | symbol | *M. musculus* | 6876 | *H. sapiens* | *TAGLN* | *transgelin* |
| *Gpr182* | 11536 | symbol | *M. musculus* | 11318 | *H. sapiens* | *GPR182* | *G protein-coupled receptor 182* |
| *Car13* | 71934 | symbol | *M. musculus* | 377677 | *H. sapiens* | *CA13* | *carbonic anhydrase 13* |
| *1700109H08Rik* | 77036 | symbol | *M. musculus* | 79645 | *H. sapiens* | *EFCAB1* | *EF-hand calcium binding domain 1* |
| *Gm16958* | 100862268 | symbol | *M. musculus* |  |  | None | None |
| *Ctxn3* | 629147 | symbol | *M. musculus* | 613212 | *H. sapiens* | *CTXN3* | *cortexin 3* |
| *Fbln5* | 23876 | symbol | *M. musculus* | 10516 | *H. sapiens* | *FBLN5* | *fibulin 5* |
| *Slc6a20a* | 102680 | symbol | *M. musculus* | 54716 | *H. sapiens* | *SLC6A20* | *solute carrier family 6 member 20* |
| *Itga5* | 16402 | symbol | *M. musculus* | 3678 | *H. sapiens* | *ITGA5* | *integrin subunit alpha 5* |
| *Fgfbp1* | 14181 | symbol | *M. musculus* | 9982 | *H. sapiens* | *FGFBP1* | *fibroblast growth factor binding protein 1* |
| *Misp* | 78906 | symbol | *M. musculus* | 126353 | *H. sapiens* | *MISP* | *mitotic spindle positioning* |
| *Cd209a* | 170786 | symbol | *M. musculus* | 10332 | *H. sapiens* | *CLEC4M* | *C-type lectin domain family 4 member M* |
| *Akr1c13* | 27384 | symbol | *M. musculus* | 8644 | *H. sapiens* | *AKR1C3* | *aldo-keto reductase family 1 member C3* |
| *Cyp1b1* | 13078 | symbol | *M. musculus* | 1545 | *H. sapiens* | *CYP1B1* | *cytochrome P450 family 1 subfamily B member 1* |
| *Adgrg6* | 215798 | symbol | *M. musculus* | 57211 | *H. sapiens* | *ADGRG6* | *adhesion G protein-coupled receptor G6* |
| *Pln* | 18821 | symbol | *M. musculus* | 5350 | *H. sapiens* | *PLN* | *phospholamban* |
| *Gm10253* | 791281 | symbol | *M. musculus* |  |  | None | None |
| *Col1a1* | 12842 | symbol | *M. musculus* | 1277 | *H. sapiens* | *COL1A1* | *collagen type I alpha 1 chain* |
| *Myh11* | 17880 | symbol | *M. musculus* | 4629 | *H. sapiens* | *MYH11* | *myosin heavy chain 11* |
| *Cd163* | 93671 | symbol | *M. musculus* | 9332 | *H. sapiens* | *CD163* | *CD163 molecule* |
| *Aox3* | 71724 | symbol | *M. musculus* | 316 | *H. sapiens* | *AOX1* | *aldehyde oxidase 1* |
| *Lyve1* | 114332 | symbol | *M. musculus* | 10894 | *H. sapiens* | *LYVE1* | *lymphatic vessel endothelial hyaluronan receptor 1* |
| *Aldh1a3* | 56847 | symbol | *M. musculus* | 220 | *H. sapiens* | *ALDH1A3* | *aldehyde dehydrogenase 1 family member A3* |
| *B230369F24Rik* | 320110 | symbol | *M. musculus* |  |  | None | None |
| *Olfml2a* | 241327 | symbol | *M. musculus* | 169611 | *H. sapiens* | *OLFML2A* | *olfactomedin like 2A* |
| *Platr14* | 74959 | symbol | *M. musculus* |  |  | None | None |
| *Gm17501* | 100216343 | symbol | *M. musculus* |  |  | None | None |
| *Slc22a3* | 20519 | symbol | *M. musculus* | 6581 | *H. sapiens* | *SLC22A3* | *solute carrier family 22 member 3* |
| *Nell1* | 338352 | symbol | *M. musculus* | 4745 | *H. sapiens* | *NELL1* | *neural EGFL like 1* |
| *Caps2* | 353025 | symbol | *M. musculus* | 84698 | *H. sapiens* | *CAPS2* | *calcyphosine 2* |
| *Gm19967* | 100503922 | symbol | *M. musculus* |  |  | None | None |
| *Cldn19* | 242653 | symbol | *M. musculus* | 149461 | *H. sapiens* | *CLDN19* | *claudin 19* |
| *Aoc3* | 11754 | symbol | *M. musculus* | 8639 | *H. sapiens* | *AOC3* | *amine oxidase copper containing 3* |
| *Coch* | 12810 | symbol | *M. musculus* | 1690 | *H. sapiens* | *COCH* | *cochlin* |
| *Tmem252* | 226040 | symbol | *M. musculus* | 169693 | *H. sapiens* | *TMEM252* | *transmembrane protein 252* |
| *Mrc1* | 17533 | symbol | *M. musculus* | 4360 | *H. sapiens* | *MRC1* | *mannose receptor C-type 1* |
| *Gm10808* | 100038470 | symbol | *M. musculus* |  |  | None | None |
| *Cbr2* | 12409 | symbol | *M. musculus* | 51181 | *H. sapiens* | *DCXR* | *dicarbonyl and L-xylulose reductase* |
| *Crabp2* | 12904 | symbol | *M. musculus* | 1382 | *H. sapiens* | *CRABP2* | *cellular retinoic acid binding protein 2* |
| *Gm10554* | 100038541 | symbol | *M. musculus* |  |  | None | None |
| *4931431C16Rik* | 74364 | symbol | *M. musculus* |  |  | None | None |
| *Itih2* | 16425 | symbol | *M. musculus* | 3698 | *H. sapiens* | *ITIH2* | *inter-alpha-trypsin inhibitor heavy chain 2* |
| *Gjb2* | 14619 | symbol | *M. musculus* | 2706 | *H. sapiens* | *GJB2* | *gap junction protein beta 2* |
| *Batf3* | 381319 | symbol | *M. musculus* | 55509 | *H. sapiens* | *BATF3* | *basic leucine zipper ATF-like transcription factor 3* |
| *Trim30d* | 209387 | symbol | *M. musculus* | 85363 | *H. sapiens* | *TRIM5* | *tripartite motif containing 5* |
| *Rad21l* | 668929 | symbol | *M. musculus* | 642636 | *H. sapiens* | *RAD21L1* | *RAD21 cohesin complex component like 1* |
| *Spata45* | 75558 | symbol | *M. musculus* | 149643 | *H. sapiens* | *SPATA45* | *spermatogenesis associated 45* |
| *B130024G19Rik* | 434198 | symbol | *M. musculus* | 644192 | *H. sapiens* | *NR2F2-AS1* | *NR2F2 antisense RNA 1* |
| *Acta2* | 11475 | symbol | *M. musculus* | 59 | *H. sapiens* | *ACTA2* | *actin alpha 2, smooth muscle* |
| *Gja6* | 414089 | symbol | *M. musculus* | 2697 | *H. sapiens* | *GJA1* | *gap junction protein alpha 1* |
| *Psma8* | 73677 | symbol | *M. musculus* | 143471 | *H. sapiens* | *PSMA8* | *proteasome 20S subunit alpha 8* |
| *2010106C02Rik* | 72104 | symbol | *M. musculus* |  |  | None | None |
| *1600029I14Rik* | 69797 | symbol | *M. musculus* |  |  | None | None |
| *Slc6a20b* | 22599 | symbol | *M. musculus* | 54716 | *H. sapiens* | *SLC6A20* | *solute carrier family 6 member 20* |
| *Gm11793* | 637008 | symbol | *M. musculus* |  |  | None | None |
| *Pcare* | 225004 | symbol | *M. musculus* | 388939 | *H. sapiens* | *PCARE* | *photoreceptor cilium actin regulator* |
| *Slc4a1* | 20533 | symbol | *M. musculus* | 6521 | *H. sapiens* | *SLC4A1* | *solute carrier family 4 member 1 (Diego blood group)* |
| *Trim12a* | 76681 | symbol | *M. musculus* | 4210 | *H. sapiens* | *MEFV* | *MEFV innate immuity regulator, pyrin* |
| *Snord49a* | 100217455 | symbol | *M. musculus* |  |  | None | None |
| *Ccdc152* | 100039139 | symbol | *M. musculus* | 100129792 | *H. sapiens* | *CCDC152* | *coiled-coil domain containing 152* |
| *Col1a2* | 12843 | symbol | *M. musculus* | 1278 | *H. sapiens* | *COL1A2* | *collagen type I alpha 2 chain* |
| *Fam180a* | 208164 | symbol | *M. musculus* | 389558 | *H. sapiens* | *FAM180A* | *family with sequence similarity 180 member A* |
| *Mir9-1* | 387133 | symbol | *M. musculus* |  |  | None | None |
| *Mlkl* | 74568 | symbol | *M. musculus* | 197259 | *H. sapiens* | *MLKL* | *mixed lineage kinase domain like pseudokinase* |
| *A230057D06Rik* | 319893 | symbol | *M. musculus* |  |  | None | None |
| *Alx3* | 11694 | symbol | *M. musculus* | 257 | *H. sapiens* | *ALX3* | *ALX homeobox 3* |
| *Alx4* | 11695 | symbol | *M. musculus* | 60529 | *H. sapiens* | *ALX4* | *ALX homeobox 4* |
| *Emilin3* | 280635 | symbol | *M. musculus* | 90187 | *H. sapiens* | *EMILIN3* | *elastin microfibril interfacer 3* |
| *Gm5134* | 333669 | symbol | *M. musculus* | 6528 | *H. sapiens* | *SLC5A5* | *solute carrier family 5 member 5* |
| *Col3a1* | 12825 | symbol | *M. musculus* | 1281 | *H. sapiens* | *COL3A1* | *collagen type III alpha 1 chain* |
| *Xlr3a* | 22445 | symbol | *M. musculus* |  |  | None | None |
| *Selenbp2* | 20342 | symbol | *M. musculus* | 8991 | *H. sapiens* | *SELENBP1* | *selenium binding protein 1* |
| *Kcnmb4os1* | 67342 | symbol | *M. musculus* |  |  | None | None |
| *Cst7* | 13011 | symbol | *M. musculus* | 8530 | *H. sapiens* | *CST7* | *cystatin F* |
| *Ccn5* | 22403 | symbol | *M. musculus* | 8839 | *H. sapiens* | *CCN5* | *cellular communication network factor 5* |
| *Pla2r1* | 18779 | symbol | *M. musculus* | 22925 | *H. sapiens* | *PLA2R1* | *phospholipase A2 receptor 1* |
| *Popdc2* | 64082 | symbol | *M. musculus* | 64091 | *H. sapiens* | *POPDC2* | *popeye domain containing 2* |
| *Pf4* | 56744 | symbol | *M. musculus* | 5196 | *H. sapiens* | *PF4* | *platelet factor 4* |
| *E130310I04Rik* | 278725 | symbol | *M. musculus* |  |  | None | None |
| *Mdfi* | 17240 | symbol | *M. musculus* | 4188 | *H. sapiens* | *MDFI* | *MyoD family inhibitor* |
| *Slc17a6* | 140919 | symbol | *M. musculus* | 57084 | *H. sapiens* | *SLC17A6* | *solute carrier family 17 member 6* |
| *Nlrc4* | 268973 | symbol | *M. musculus* | 58484 | *H. sapiens* | *NLRC4* | *NLR family CARD domain containing 4* |
| *AI182371* | 98870 | symbol | *M. musculus* | 727 | *H. sapiens* | *C5* | *complement C5* |
| *Slc6a13* | 14412 | symbol | *M. musculus* | 6540 | *H. sapiens* | *SLC6A13* | *solute carrier family 6 member 13* |
| *Wfikkn2* | 278507 | symbol | *M. musculus* | 124857 | *H. sapiens* | *WFIKKN2* | *WAP, follistatin/kazal, immunoglobulin, kunitz and netrin domain containing 2* |
| *Siglec1* | 20612 | symbol | *M. musculus* | 6614 | *H. sapiens* | *SIGLEC1* | *sialic acid binding Ig like lectin 1* |
| *Gm19937* | 100503873 | symbol | *M. musculus* |  |  | None | None |
| *BC049762* | 193286 | symbol | *M. musculus* |  |  | None | None |
| *Hbb-b1* | 15129 | symbol | *M. musculus* |  |  | None | None |
| *Casp4* | 12363 | symbol | *M. musculus* | 837 | *H. sapiens* | *CASP4* | *caspase 4* |
| *Scpep1os* | 73921 | symbol | *M. musculus* |  |  | None | None |
| *Spin4* | 270624 | symbol | *M. musculus* | 139886 | *H. sapiens* | *SPIN4* | *spindlin family member 4* |
| *Mpzl2* | 14012 | symbol | *M. musculus* | 10205 | *H. sapiens* | *MPZL2* | *myelin protein zero like 2* |
| *Ptgdr* | 19214 | symbol | *M. musculus* | 5729 | *H. sapiens* | *PTGDR* | *prostaglandin D2 receptor* |
| *3010003L21Rik* | 109163 | symbol | *M. musculus* |  |  | None | None |
| *LOC668415* | 668415 | Gene_ID | *M. musculus* |  |  | None | None |
| *H2-Ab1* | 14961 | symbol | *M. musculus* | 3119 | *H. sapiens* | *HLA-DQB1* | *major histocompatibility complex, class II, DQ beta 1* |
| *Nphs2* | 170484 | symbol | *M. musculus* | 7827 | *H. sapiens* | *NPHS2* | *NPHS2 stomatin family member, podocin* |
| *Vmn2r57* | 269902 | symbol | *M. musculus* | 846 | *H. sapiens* | *CASR* | *calcium sensing receptor* |
| *Col28a1* | 213945 | symbol | *M. musculus* | 340267 | *H. sapiens* | *COL28A1* | *collagen type XXVIII alpha 1 chain* |
| *Prm1* | 19118 | symbol | *M. musculus* | 5619 | *H. sapiens* | *PRM1* | *protamine 1* |
| *H3c13* | 319154 | symbol | *M. musculus* | 653604 | *H. sapiens* | *H3C13* | *H3 clustered histone 13* |
| *Got1l1* | 76615 | symbol | *M. musculus* | 137362 | *H. sapiens* | *GOT1L1* | *glutamic-oxaloacetic transaminase 1 like 1* |
| *Slc6a12* | 14411 | symbol | *M. musculus* | 6539 | *H. sapiens* | *SLC6A12* | *solute carrier family 6 member 12* |
| *Slc12a1* | 20495 | symbol | *M. musculus* | 6557 | *H. sapiens* | *SLC12A1* | *solute carrier family 12 member 1* |
| *Aoah* | 27052 | symbol | *M. musculus* | 313 | *H. sapiens* | *AOAH* | *acyloxyacyl hydrolase* |
| *Gm19351* | 100502750 | symbol | *M. musculus* |  |  | None | None |
| *Il7* | 16196 | symbol | *M. musculus* | 3574 | *H. sapiens* | *IL7* | *interleukin 7* |
| *Fmod* | 14264 | symbol | *M. musculus* | 2331 | *H. sapiens* | *FMOD* | *fibromodulin* |
| *Phf11d* | 219132 | symbol | *M. musculus* | 51131 | *H. sapiens* | *PHF11* | *PHD finger protein 11* |
| *Gm10855* | 100038708 | symbol | *M. musculus* |  |  | None | None |
| *Foxc2* | 14234 | symbol | *M. musculus* | 2303 | *H. sapiens* | *FOXC2* | *forkhead box C2* |
| *Slc22a6* | 18399 | symbol | *M. musculus* | 9356 | *H. sapiens* | *SLC22A6* | *solute carrier family 22 member 6* |
| *Pou2af1* | 18985 | symbol | *M. musculus* | 5450 | *H. sapiens* | *POU2AF1* | *POU class 2 homeobox associating factor 1* |
| *Phf11* | 628693 | symbol | *M. musculus* |  |  | None | None |
| *Esm1* | 71690 | symbol | *M. musculus* | 11082 | *H. sapiens* | *ESM1* | *endothelial cell specific molecule 1* |
| *Adcy10* | 271639 | symbol | *M. musculus* | 55811 | *H. sapiens* | *ADCY10* | *adenylate cyclase 10* |
| *Slc13a4* | 243755 | symbol | *M. musculus* | 26266 | *H. sapiens* | *SLC13A4* | *solute carrier family 13 member 4* |
| *Gm12247* | 667947 | symbol | *M. musculus* |  |  | None | None |
| *Slc25a21* | 217593 | symbol | *M. musculus* | 89874 | *H. sapiens* | *SLC25A21* | *solute carrier family 25 member 21* |
| *Slc18a3* | 20508 | symbol | *M. musculus* | 6572 | *H. sapiens* | *SLC18A3* | *solute carrier family 18 member A3* |
| *Hspb7* | 29818 | symbol | *M. musculus* | 27129 | *H. sapiens* | *HSPB7* | *heat shock protein family B (small) member 7* |
| *Il31ra* | 218624 | symbol | *M. musculus* | 133396 | *H. sapiens* | *IL31RA* | *interleukin 31 receptor A* |
| *Hal* | 15109 | symbol | *M. musculus* | 3034 | *H. sapiens* | *HAL* | *histidine ammonia-lyase* |
| *Gm4544* | 100043600 | symbol | *M. musculus* |  |  | None | None |
| *Ankrd22* | 52024 | symbol | *M. musculus* | 118932 | *H. sapiens* | *ANKRD22* | *ankyrin repeat domain 22* |
| *Cdh1* | 12550 | symbol | *M. musculus* | 999 | *H. sapiens* | *CDH1* | *cadherin 1* |
| *Dennd2d* | 72121 | symbol | *M. musculus* | 79961 | *H. sapiens* | *DENND2D* | *DENN domain containing 2D* |
| *Dlx3* | 13393 | symbol | *M. musculus* | 1747 | *H. sapiens* | *DLX3* | *distal-less homeobox 3* |
| *Aldh1a2* | 19378 | symbol | *M. musculus* | 8854 | *H. sapiens* | *ALDH1A2* | *aldehyde dehydrogenase 1 family member A2* |
| *Tmem51os1* | 100038693 | symbol | *M. musculus* |  |  | None | None |
| *Ccr2* | 12772 | symbol | *M. musculus* | 729230 | *H. sapiens* | *CCR2* | *C-C motif chemokine receptor 2* |
| *Ipw* | 16353 | symbol | *M. musculus* |  |  | None | None |
| *Zfp957* | 105590 | symbol | *M. musculus* | 6942 | *H. sapiens* | *TCF20* | *transcription factor 20* |
| *Upb1* | 103149 | symbol | *M. musculus* | 51733 | *H. sapiens* | *UPB1* | *beta-ureidopropionase 1* |
| *Foxd2* | 17301 | symbol | *M. musculus* | 2306 | *H. sapiens* | *FOXD2* | *forkhead box D2* |
| *Clec12a* | 232413 | symbol | *M. musculus* | 160364 | *H. sapiens* | *CLEC12A* | *C-type lectin domain family 12 member A* |
| *Clec3a* | 403395 | symbol | *M. musculus* | 10143 | *H. sapiens* | *CLEC3A* | *C-type lectin domain family 3 member A* |
| *Gpr152* | 269053 | symbol | *M. musculus* | 390212 | *H. sapiens* | *GPR152* | *G protein-coupled receptor 152* |
| *H2ac19* | 319192 | symbol | *M. musculus* | 723790 | *H. sapiens* | *H2AC19* | *H2A clustered histone 19* |
| *Cma1* | 17228 | symbol | *M. musculus* | 1215 | *H. sapiens* | *CMA1* | *chymase 1* |
| *Nkx6-1* | 18096 | symbol | *M. musculus* | 4825 | *H. sapiens* | *NKX6-1* | *NK6 homeobox 1* |
| *Cpa3* | 12873 | symbol | *M. musculus* | 1359 | *H. sapiens* | *CPA3* | *carboxypeptidase A3* |
| *Rab19* | 19331 | symbol | *M. musculus* | 401409 | *H. sapiens* | *RAB19* | *RAB19, member RAS oncogene family* |
| *Wnt6* | 22420 | symbol | *M. musculus* | 7475 | *H. sapiens* | *WNT6* | *Wnt family member 6* |
| *Lox* | 16948 | symbol | *M. musculus* | 4015 | *H. sapiens* | *LOX* | *lysyl oxidase* |
| *Apobec2* | 11811 | symbol | *M. musculus* | 10930 | *H. sapiens* | *APOBEC2* | *apolipoprotein B mRNA editing enzyme catalytic subunit 2* |
| *2310001H17Rik* | 76432 | symbol | *M. musculus* |  |  | None | None |
| *Mcpt4* | 17227 | symbol | *M. musculus* | 354 | *H. sapiens* | *KLK3* | *kallikrein related peptidase 3* |
| *Tpsb2* | 17229 | symbol | *M. musculus* | 7177 | *H. sapiens* | *TPSAB1* | *tryptase alpha/beta 1* |
| *Asmt* | 107626 | symbol | *M. musculus* | 438 | *H. sapiens* | *ASMT* | *acetylserotonin O-methyltransferase* |
