# Supplementary material for: Neural EGFL-like 1, a craniosynostosis-related osteochondrogenic molecule, strikingly associates with neurodevelopmental pathologies
Source: Cell Biosci. 2023 Dec 15;13:227. doi: 10.1186/s13578-023-01174-5 (PMC10725010; doi:10.1186/s13578-023-01174-5)
Supplement: Supplementary file 11 — Additional file 11: Table S4.The result of pathway and process enrichment analysis with the downregulated DEGs. [file 13578_2023_1174_MOESM11_ESM.docx]

Table S4. The result of pathway and process enrichment analysis with the downregulated DEGs.

| **GroupID** | **Category** | **Term** | **Description** | **Log_10_P** | **Log_10_(q-value)** | **InTerm_InList** | **Symbols** |
| --- | --- | --- | --- | --- | --- | --- | --- |
| 1_Summary | Reactome Gene Sets | R-HSA-1474244 | Extracellular matrix organization | -12.76174986 | -8.405 | 18/301 | BMP4, BMP7, CDH1, CMA1, COL1A1, COL1A2, COL3A1, COL9A2, ELN, FMOD, ITGA5, LOX, LUM, TPSAB1, VWF, FBLN5, EMILIN3, COL28A1, CYP1B1, FOXC2, MYH11, OLFML2A, COLEC12, CD163, SIGLEC1, CLEC2D, CPA3, DES, EDN1, MYO7A, LAT, PF4, SLC7A11, HLA-DQB1, MRC1, MRC2, PSMA8, SERPING1, ACTA2, PRM1 |
| 1_Member | Reactome Gene Sets | R-HSA-1474244 | Extracellular matrix organization | -12.76174986 | -8.405 | 18/301 | BMP4, BMP7, CDH1, CMA1, COL1A1, COL1A2, COL3A1, COL9A2, ELN, FMOD, ITGA5, LOX, LUM, TPSAB1, VWF, FBLN5, EMILIN3, COL28A1 |
| 1_Member | GO Biological Processes | GO:0030198 | extracellular matrix organization | -10.59789612 | -6.876 | 16/302 | CMA1, COL1A1, COL1A2, COL3A1, COL9A2, CYP1B1, ELN, FOXC2, FMOD, LOX, LUM, MYH11, TPSAB1, FBLN5, OLFML2A, COL28A1 |
| 1_Member | GO Biological Processes | GO:0043062 | extracellular structure organization | -10.57647019 | -6.876 | 16/303 | CMA1, COL1A1, COL1A2, COL3A1, COL9A2, CYP1B1, ELN, FOXC2, FMOD, LOX, LUM, MYH11, TPSAB1, FBLN5, OLFML2A, COL28A1 |
| 1_Member | GO Biological Processes | GO:0045229 | external encapsulating structure organization | -10.53385678 | -6.876 | 16/305 | CMA1, COL1A1, COL1A2, COL3A1, COL9A2, CYP1B1, ELN, FOXC2, FMOD, LOX, LUM, MYH11, TPSAB1, FBLN5, OLFML2A, COL28A1 |
| 1_Member | GO Biological Processes | GO:0030199 | collagen fibril organization | -8.63980635 | -5.324 | 8/61 | COL1A1, COL1A2, COL3A1, CYP1B1, FOXC2, FMOD, LOX, LUM |
| 1_Member | Reactome Gene Sets | R-HSA-216083 | Integrin cell surface interactions | -7.477203895 | -4.324 | 8/85 | CDH1, COL1A1, COL1A2, COL3A1, COL9A2, ITGA5, LUM, VWF |
| 1_Member | Reactome Gene Sets | R-HSA-1474228 | Degradation of the extracellular matrix | -5.800558118 | -3.023 | 8/140 | CDH1, CMA1, COL1A1, COL1A2, COL3A1, COL9A2, ELN, TPSAB1 |
| 1_Member | Reactome Gene Sets | R-HSA-3000480 | Scavenging by Class A Receptors | -5.417474399 | -2.768 | 4/19 | COL1A1, COL1A2, COL3A1, COLEC12 |
| 1_Member | Reactome Gene Sets | R-HSA-2173782 | Binding and Uptake of Ligands by Scavenger Receptors | -5.372437015 | -2.740 | 5/42 | COL1A1, COL1A2, COL3A1, CD163, COLEC12 |
| 1_Member | Reactome Gene Sets | R-HSA-3000178 | ECM proteoglycans | -5.285836355 | -2.677 | 6/76 | COL1A1, COL1A2, COL3A1, COL9A2, FMOD, LUM |
| 1_Member | Reactome Gene Sets | R-HSA-8948216 | Collagen chain trimerization | -5.270393673 | -2.669 | 5/44 | COL1A1, COL1A2, COL3A1, COL9A2, COL28A1 |
| 1_Member | Reactome Gene Sets | R-HSA-198933 | Immunoregulatory interactions between a Lymphoid and a non-Lymphoid cell | -4.927598591 | -2.440 | 7/132 | CDH1, COL1A1, COL1A2, COL3A1, SIGLEC1, CLEC2D, COLEC12 |
| 1_Member | Reactome Gene Sets | R-HSA-1474290 | Collagen formation | -4.860089919 | -2.395 | 6/90 | COL1A1, COL1A2, COL3A1, COL9A2, LOX, COL28A1 |
| 1_Member | KEGG Pathway | ko04974 | Protein digestion and absorption | -4.860089919 | -2.395 | 6/90 | COL1A1, COL1A2, COL3A1, COL9A2, CPA3, ELN |
| 1_Member | KEGG Pathway | hsa04974 | Protein digestion and absorption | -4.75178778 | -2.349 | 6/94 | COL1A1, COL1A2, COL3A1, COL9A2, CPA3, ELN |
| 1_Member | Reactome Gene Sets | R-HSA-2022090 | Assembly of collagen fibrils and other multimeric structures | -4.565987702 | -2.209 | 5/61 | COL1A1, COL1A2, COL3A1, COL9A2, LOX |
| 1_Member | Reactome Gene Sets | R-HSA-430116 | GP1b-IX-V activation signalling | -4.40254011 | -2.083 | 3/12 | COL1A1, COL1A2, VWF |
| 1_Member | Reactome Gene Sets | R-HSA-1650814 | Collagen biosynthesis and modifying enzymes | -4.367631697 | -2.069 | 5/67 | COL1A1, COL1A2, COL3A1, COL9A2, COL28A1 |
| 1_Member | Reactome Gene Sets | R-HSA-75892 | Platelet Adhesion to exposed collagen | -4.092466842 | -1.853 | 3/15 | COL1A1, COL1A2, VWF |
| 1_Member | GO Biological Processes | GO:0097435 | supramolecular fiber organization | -3.95482668 | -1.757 | 14/746 | COL1A1, COL1A2, COL3A1, CYP1B1, DES, EDN1, ELN, FOXC2, FMOD, LOX, LUM, MYH11, MYO7A, FBLN5 |
| 1_Member | KEGG Pathway | hsa04512 | ECM-receptor interaction | -3.946689522 | -1.757 | 5/82 | COL1A1, COL1A2, COL9A2, ITGA5, VWF |
| 1_Member | KEGG Pathway | ko04512 | ECM-receptor interaction | -3.946689522 | -1.757 | 5/82 | COL1A1, COL1A2, COL9A2, ITGA5, VWF |
| 1_Member | Reactome Gene Sets | R-HSA-2243919 | Crosslinking of collagen fibrils | -3.84429923 | -1.694 | 3/18 | COL1A1, COL1A2, LOX |
| 1_Member | Reactome Gene Sets | R-HSA-3000170 | Syndecan interactions | -3.306371028 | -1.319 | 3/27 | COL1A1, COL1A2, COL3A1 |
| 1_Member | Reactome Gene Sets | R-HSA-1442490 | Collagen degradation | -3.2902297 | -1.307 | 4/64 | COL1A1, COL1A2, COL3A1, COL9A2 |
| 1_Member | Reactome Gene Sets | R-HSA-8874081 | MET activates PTK2 signaling | -3.16946467 | -1.221 | 3/30 | COL1A1, COL1A2, COL3A1 |
| 1_Member | WikiPathways | WP453 | Inflammatory response pathway | -3.046605772 | -1.145 | 3/33 | COL1A1, COL1A2, COL3A1 |
| 1_Member | WikiPathways | WP4786 | Type I collagen synthesis in the context of osteogenesis imperfecta | -3.046605772 | -1.145 | 3/33 | COL1A1, COL1A2, LOX |
| 1_Member | Reactome Gene Sets | R-HSA-114604 | GPVI-mediated activation cascade | -2.971227665 | -1.089 | 3/35 | COL1A1, COL1A2, LAT |
| 1_Member | Reactome Gene Sets | R-HSA-202733 | Cell surface interactions at the vascular wall | -2.92022064 | -1.056 | 5/137 | COL1A1, COL1A2, ITGA5, PF4, SLC7A11 |
| 1_Member | Reactome Gene Sets | R-HSA-76009 | Platelet Aggregation (Plug Formation) | -2.833547132 | -1.000 | 3/39 | COL1A1, COL1A2, VWF |
| 1_Member | Reactome Gene Sets | R-HSA-1280218 | Adaptive Immune System | -2.796106468 | -0.973 | 12/763 | CDH1, COL1A1, COL1A2, COL3A1, HLA-DQB1, MRC1, SIGLEC1, MRC2, LAT, CLEC2D, COLEC12, PSMA8 |
| 1_Member | Reactome Gene Sets | R-HSA-8875878 | MET promotes cell motility | -2.770339906 | -0.959 | 3/41 | COL1A1, COL1A2, COL3A1 |
| 1_Member | KEGG Pathway | ko04933 | AGE-RAGE signaling pathway in diabetic complications | -2.585080539 | -0.828 | 4/99 | COL1A1, COL1A2, COL3A1, EDN1 |
| 1_Member | GO Biological Processes | GO:0032963 | collagen metabolic process | -2.507802849 | -0.774 | 4/104 | BMP4, COL1A1, COL1A2, MRC2 |
| 1_Member | KEGG Pathway | hsa04933 | AGE-RAGE signaling pathway in diabetic complications | -2.463444569 | -0.741 | 4/107 | COL1A1, COL1A2, COL3A1, EDN1 |
| 1_Member | Reactome Gene Sets | R-HSA-76002 | Platelet activation, signaling and aggregation | -2.369758758 | -0.675 | 6/263 | SERPING1, COL1A1, COL1A2, PF4, VWF, LAT |
| 1_Member | Reactome Gene Sets | R-HSA-3000171 | Non-integrin membrane-ECM interactions | -2.318866047 | -0.640 | 3/59 | COL1A1, COL1A2, COL3A1 |
| 1_Member | WikiPathways | WP4754 | IL-18 signaling pathway | -2.246780093 | -0.589 | 6/279 | ACTA2, COL1A1, COL1A2, COL3A1, DES, PRM1 |
| 1_Member | KEGG Pathway | hsa04510 | Focal adhesion | -2.222554289 | -0.573 | 5/199 | COL1A1, COL1A2, COL9A2, ITGA5, VWF |
| 1_Member | KEGG Pathway | ko04510 | Focal adhesion | -2.222554289 | -0.573 | 5/199 | COL1A1, COL1A2, COL9A2, ITGA5, VWF |
| 1_Member | KEGG Pathway | hsa04611 | Platelet activation | -2.164878127 | -0.535 | 4/130 | COL1A1, COL1A2, COL3A1, VWF |
| 2_Summary | GO Biological Processes | GO:0003013 | circulatory system process | -11.26256271 | -7.207 | 22/598 | ACTA2, KLK3, BMP6, SERPING1, CASR, CMA1, COL1A2, CPA3, DES, EDN1, ELN, FOXC2, GJA1, PLN, PPARG, SLC5A5, SLC6A13, SLC22A3, TBX18, HSPB7, SLC6A20, POPDC2, CST7 |
| 2_Member | GO Biological Processes | GO:0003013 | circulatory system process | -11.26256271 | -7.207 | 22/598 | ACTA2, KLK3, BMP6, SERPING1, CASR, CMA1, COL1A2, CPA3, DES, EDN1, ELN, FOXC2, GJA1, PLN, PPARG, SLC5A5, SLC6A13, SLC22A3, TBX18, HSPB7, SLC6A20, POPDC2 |
| 2_Member | GO Biological Processes | GO:0008015 | blood circulation | -8.9503792 | -5.497 | 18/513 | ACTA2, KLK3, BMP6, SERPING1, CASR, CMA1, COL1A2, CPA3, DES, EDN1, ELN, FOXC2, GJA1, PLN, PPARG, TBX18, HSPB7, POPDC2 |
| 2_Member | GO Biological Processes | GO:1903522 | regulation of blood circulation | -3.895475281 | -1.720 | 8/257 | CASR, DES, EDN1, GJA1, PLN, TBX18, HSPB7, POPDC2 |
| 2_Member | GO Biological Processes | GO:0008016 | regulation of heart contraction | -3.690066267 | -1.600 | 7/207 | DES, EDN1, GJA1, PLN, TBX18, HSPB7, POPDC2 |
| 2_Member | GO Biological Processes | GO:0060047 | heart contraction | -3.281193352 | -1.301 | 7/242 | DES, EDN1, GJA1, PLN, TBX18, HSPB7, POPDC2 |
| 2_Member | GO Biological Processes | GO:0003015 | heart process | -3.177270424 | -1.225 | 7/252 | DES, EDN1, GJA1, PLN, TBX18, HSPB7, POPDC2 |
| 2_Member | GO Biological Processes | GO:0044057 | regulation of system process | -2.690115141 | -0.899 | 10/581 | BMP6, CASR, DES, EDN1, GJA1, PLN, CST7, TBX18, HSPB7, POPDC2 |
| 3_Summary | GO Biological Processes | GO:0001501 | skeletal system development | -9.891126306 | -6.312 | 19/508 | ALX3, BMP4, BMP6, BMP7, COL1A1, COL1A2, COL3A1, COL9A2, EDN1, FOXC2, GJA1, LOX, LUM, MDFI, MGP, CLEC3A, ALX4, LRRK1, WFIKKN2, ATP6V1B1, CASR, SMAD6, NELL1, ENPP1, PPARG, MRC2, ACTA2, FOXD1, WNT6, MPZL2, HSPB7, DLX3, NKX6-1, IL31RA, ALDH1A2, FGFBP1, CDH1, ITGA5, LAT, CARD14, CLDN19, SLC4A1, CYP1B1, LRRC32 |
| 3_Member | GO Biological Processes | GO:0001501 | skeletal system development | -9.891126306 | -6.312 | 19/508 | ALX3, BMP4, BMP6, BMP7, COL1A1, COL1A2, COL3A1, COL9A2, EDN1, FOXC2, GJA1, LOX, LUM, MDFI, MGP, CLEC3A, ALX4, LRRK1, WFIKKN2 |
| 3_Member | GO Biological Processes | GO:0001503 | ossification | -8.642223105 | -5.324 | 16/411 | ATP6V1B1, BMP4, BMP6, BMP7, CASR, COL1A1, COL1A2, FOXC2, LOX, SMAD6, MGP, NELL1, ENPP1, PPARG, MRC2, CLEC3A |
| 3_Member | GO Biological Processes | GO:0061448 | connective tissue development | -7.498176658 | -4.324 | 12/255 | ACTA2, BMP4, BMP6, BMP7, CASR, COL1A1, EDN1, FOXD1, FOXC2, LOX, LUM, MGP |
| 3_Member | GO Biological Processes | GO:0031214 | biomineral tissue development | -7.26183519 | -4.135 | 10/169 | BMP4, BMP6, BMP7, COL1A1, COL1A2, LOX, MGP, NELL1, ENPP1, WNT6 |
| 3_Member | GO Biological Processes | GO:0110148 | biomineralization | -7.213602462 | -4.112 | 10/171 | BMP4, BMP6, BMP7, COL1A1, COL1A2, LOX, MGP, NELL1, ENPP1, WNT6 |
| 3_Member | WikiPathways | WP2895 | Differentiation of white and brown adipocyte | -6.542570608 | -3.547 | 5/25 | BMP4, BMP7, PPARG, MPZL2, HSPB7 |
| 3_Member | GO Biological Processes | GO:0030282 | bone mineralization | -6.336878765 | -3.395 | 8/119 | BMP4, BMP6, BMP7, COL1A2, LOX, MGP, NELL1, ENPP1 |
| 3_Member | GO Biological Processes | GO:0070167 | regulation of biomineral tissue development | -5.817140438 | -3.023 | 7/97 | BMP4, BMP6, BMP7, MGP, NELL1, ENPP1, WNT6 |
| 3_Member | GO Biological Processes | GO:0110149 | regulation of biomineralization | -5.757284317 | -3.002 | 7/99 | BMP4, BMP6, BMP7, MGP, NELL1, ENPP1, WNT6 |
| 3_Member | GO Biological Processes | GO:0030278 | regulation of ossification | -5.296927613 | -2.680 | 7/116 | BMP4, BMP6, BMP7, SMAD6, MGP, NELL1, ENPP1 |
| 3_Member | GO Biological Processes | GO:0030500 | regulation of bone mineralization | -5.219955926 | -2.634 | 6/78 | BMP4, BMP6, BMP7, MGP, NELL1, ENPP1 |
| 3_Member | GO Biological Processes | GO:0001649 | osteoblast differentiation | -5.101347008 | -2.537 | 9/230 | BMP4, BMP6, BMP7, COL1A1, LOX, SMAD6, NELL1, PPARG, MRC2 |
| 3_Member | GO Biological Processes | GO:0070169 | positive regulation of biomineral tissue development | -5.035972534 | -2.499 | 5/49 | BMP4, BMP6, BMP7, NELL1, WNT6 |
| 3_Member | GO Biological Processes | GO:0110151 | positive regulation of biomineralization | -4.992228402 | -2.478 | 5/50 | BMP4, BMP6, BMP7, NELL1, WNT6 |
| 3_Member | GO Biological Processes | GO:0060393 | regulation of pathway-restricted SMAD protein phosphorylation | -4.531481544 | -2.187 | 5/62 | BMP4, BMP6, BMP7, SMAD6, PPARG |
| 3_Member | GO Biological Processes | GO:0060389 | pathway-restricted SMAD protein phosphorylation | -4.431509594 | -2.108 | 5/65 | BMP4, BMP6, BMP7, SMAD6, PPARG |
| 3_Member | GO Biological Processes | GO:0030501 | positive regulation of bone mineralization | -4.085829137 | -1.853 | 4/40 | BMP4, BMP6, BMP7, NELL1 |
| 3_Member | GO Biological Processes | GO:0045667 | regulation of osteoblast differentiation | -3.924414317 | -1.738 | 6/132 | BMP4, BMP6, BMP7, SMAD6, NELL1, PPARG |
| 3_Member | GO Biological Processes | GO:0060395 | SMAD protein signal transduction | -3.921711907 | -1.738 | 5/83 | BMP4, BMP6, BMP7, SMAD6, PPARG |
| 3_Member | GO Biological Processes | GO:0051216 | cartilage development | -3.905183385 | -1.724 | 7/191 | BMP4, BMP6, BMP7, COL1A1, EDN1, LUM, MGP |
| 3_Member | GO Biological Processes | GO:0090092 | regulation of transmembrane receptor protein serine/threonine kinase signaling pathway | -3.895475281 | -1.720 | 8/257 | BMP4, BMP6, BMP7, FOXD1, LOX, SMAD6, PPARG, WFIKKN2 |
| 3_Member | GO Biological Processes | GO:0060391 | positive regulation of SMAD protein signal transduction | -3.84429923 | -1.694 | 3/18 | BMP4, BMP6, PPARG |
| 3_Member | GO Biological Processes | GO:0010862 | positive regulation of pathway-restricted SMAD protein phosphorylation | -3.738118026 | -1.624 | 4/49 | BMP4, BMP6, BMP7, PPARG |
| 3_Member | GO Biological Processes | GO:0045778 | positive regulation of ossification | -3.738118026 | -1.624 | 4/49 | BMP4, BMP6, BMP7, NELL1 |
| 3_Member | GO Biological Processes | GO:0002065 | columnar/cuboidal epithelial cell differentiation | -3.733168737 | -1.622 | 5/91 | BMP4, BMP6, DLX3, NKX6-1, IL31RA |
| 3_Member | GO Biological Processes | GO:0030509 | BMP signaling pathway | -3.606501722 | -1.548 | 6/151 | BMP4, BMP6, BMP7, FOXD1, SMAD6, PPARG |
| 3_Member | GO Biological Processes | GO:0002067 | glandular epithelial cell differentiation | -3.5430086 | -1.508 | 4/55 | BMP4, BMP6, NKX6-1, IL31RA |
| 3_Member | GO Biological Processes | GO:0003323 | type B pancreatic cell development | -3.516946486 | -1.486 | 3/23 | BMP4, BMP6, NKX6-1 |
| 3_Member | GO Biological Processes | GO:0071772 | response to BMP | -3.414365801 | -1.404 | 6/164 | BMP4, BMP6, BMP7, FOXD1, SMAD6, PPARG |
| 3_Member | GO Biological Processes | GO:0071773 | cellular response to BMP stimulus | -3.414365801 | -1.404 | 6/164 | BMP4, BMP6, BMP7, FOXD1, SMAD6, PPARG |
| 3_Member | GO Biological Processes | GO:0001958 | endochondral ossification | -3.306371028 | -1.319 | 3/27 | BMP4, BMP6, COL1A1 |
| 3_Member | GO Biological Processes | GO:0036075 | replacement ossification | -3.306371028 | -1.319 | 3/27 | BMP4, BMP6, COL1A1 |
| 3_Member | GO Biological Processes | GO:0090100 | positive regulation of transmembrane receptor protein serine/threonine kinase signaling pathway | -3.279954441 | -1.301 | 5/114 | BMP4, BMP6, BMP7, FOXD1, PPARG |
| 3_Member | WikiPathways | WP474 | Endochondral ossification | -3.264580898 | -1.290 | 4/65 | BMP6, BMP7, MGP, ENPP1 |
| 3_Member | WikiPathways | WP4808 | Endochondral ossification with skeletal dysplasias | -3.264580898 | -1.290 | 4/65 | BMP6, BMP7, MGP, ENPP1 |
| 3_Member | GO Biological Processes | GO:0002068 | glandular epithelial cell development | -3.213391378 | -1.254 | 3/29 | BMP4, BMP6, NKX6-1 |
| 3_Member | GO Biological Processes | GO:0003309 | type B pancreatic cell differentiation | -3.213391378 | -1.254 | 3/29 | BMP4, BMP6, NKX6-1 |
| 3_Member | GO Biological Processes | GO:0045669 | positive regulation of osteoblast differentiation | -3.16616525 | -1.220 | 4/69 | BMP4, BMP6, BMP7, NELL1 |
| 3_Member | GO Biological Processes | GO:0001936 | regulation of endothelial cell proliferation | -3.138644638 | -1.198 | 6/185 | BMP4, BMP6, GJA1, PPARG, ALDH1A2, FGFBP1 |
| 3_Member | GO Biological Processes | GO:0060390 | regulation of SMAD protein signal transduction | -3.127091305 | -1.195 | 3/31 | BMP4, BMP6, PPARG |
| 3_Member | GO Biological Processes | GO:0035883 | enteroendocrine cell differentiation | -3.086169134 | -1.165 | 3/32 | BMP4, BMP6, NKX6-1 |
| 3_Member | GO Biological Processes | GO:0035270 | endocrine system development | -3.067419177 | -1.153 | 5/127 | BMP4, BMP6, CDH1, NKX6-1, ALDH1A2 |
| 3_Member | GO Biological Processes | GO:0031016 | pancreas development | -2.986999546 | -1.094 | 4/77 | BMP4, BMP6, NKX6-1, ALDH1A2 |
| 3_Member | GO Biological Processes | GO:0001935 | endothelial cell proliferation | -2.96336225 | -1.085 | 6/200 | BMP4, BMP6, GJA1, PPARG, ALDH1A2, FGFBP1 |
| 3_Member | GO Biological Processes | GO:0030224 | monocyte differentiation | -2.935266963 | -1.067 | 3/36 | BMP4, PPARG, IL31RA |
| 3_Member | GO Biological Processes | GO:0060348 | bone development | -2.897584863 | -1.044 | 6/206 | BMP4, BMP6, COL1A1, GJA1, LOX, LRRK1 |
| 3_Member | KEGG Pathway | hsa04350 | TGF-beta signaling pathway | -2.846445744 | -1.009 | 4/84 | BMP4, BMP6, BMP7, SMAD6 |
| 3_Member | KEGG Pathway | ko04350 | TGF-beta signaling pathway | -2.846445744 | -1.009 | 4/84 | BMP4, BMP6, BMP7, SMAD6 |
| 3_Member | KEGG Pathway | ko04390 | Hippo signaling pathway | -2.696487103 | -0.904 | 5/154 | BMP4, BMP6, BMP7, CDH1, WNT6 |
| 3_Member | GO Biological Processes | GO:0002066 | columnar/cuboidal epithelial cell development | -2.681551197 | -0.896 | 3/44 | BMP4, BMP6, NKX6-1 |
| 3_Member | GO Biological Processes | GO:0045666 | positive regulation of neuron differentiation | -2.66692583 | -0.886 | 4/94 | BMP4, BMP6, BMP7, NKX6-1 |
| 3_Member | GO Biological Processes | GO:0030510 | regulation of BMP signaling pathway | -2.650168098 | -0.878 | 4/95 | BMP4, FOXD1, SMAD6, PPARG |
| 3_Member | GO Biological Processes | GO:0031018 | endocrine pancreas development | -2.625944967 | -0.859 | 3/46 | BMP4, BMP6, NKX6-1 |
| 3_Member | KEGG Pathway | hsa04390 | Hippo signaling pathway | -2.467760392 | -0.744 | 5/174 | BMP4, BMP6, BMP7, CDH1, WNT6 |
| 3_Member | GO Biological Processes | GO:0001934 | positive regulation of protein phosphorylation | -2.453811346 | -0.734 | 11/732 | BMP4, BMP6, BMP7, EDN1, ITGA5, PPARG, LAT, CARD14, LRRK1, IL31RA, CLDN19 |
| 3_Member | GO Biological Processes | GO:0060350 | endochondral bone morphogenesis | -2.427260642 | -0.713 | 3/54 | BMP4, BMP6, COL1A1 |
| 3_Member | GO Biological Processes | GO:0061614 | pri-miRNA transcription by RNA polymerase II | -2.404719756 | -0.699 | 3/55 | BMP4, SMAD6, PPARG |
| 3_Member | GO Biological Processes | GO:1902893 | regulation of pri-miRNA transcription by RNA polymerase II | -2.404719756 | -0.699 | 3/55 | BMP4, SMAD6, PPARG |
| 3_Member | GO Biological Processes | GO:0043407 | negative regulation of MAP kinase activity | -2.318866047 | -0.640 | 3/59 | BMP4, BMP7, PPARG |
| 3_Member | GO Biological Processes | GO:0030858 | positive regulation of epithelial cell differentiation | -2.220183422 | -0.573 | 3/64 | BMP4, BMP6, NKX6-1 |
| 3_Member | GO Biological Processes | GO:0042326 | negative regulation of phosphorylation | -2.150017353 | -0.523 | 7/385 | BMP4, BMP7, SMAD6, ENPP1, PPARG, SLC4A1, LRRK1 |
| 3_Member | GO Biological Processes | GO:0060411 | cardiac septum morphogenesis | -2.147218672 | -0.522 | 3/68 | BMP4, BMP7, SMAD6 |
| 3_Member | GO Biological Processes | GO:0007162 | negative regulation of cell adhesion | -2.078304552 | -0.467 | 6/303 | BMP4, BMP6, CDH1, COL1A1, CYP1B1, LRRC32 |
| 4_Summary | GO Biological Processes | GO:0006820 | anion transport | -9.004095077 | -5.497 | 18/509 | CASR, EDN1, GJA1, ENPP1, SLC4A1, SLC5A5, SLC6A12, SLC6A13, SLC12A1, SLC22A3, SLC22A6, PLA2R1, SLC7A11, SLC13A4, SLC6A20, SLC17A6, SLC25A21, ANO5, CP, PPARG, ACTA2, BMP6, FOXC2 |
| 4_Member | GO Biological Processes | GO:0006820 | anion transport | -9.004095077 | -5.497 | 18/509 | CASR, EDN1, GJA1, ENPP1, SLC4A1, SLC5A5, SLC6A12, SLC6A13, SLC12A1, SLC22A3, SLC22A6, PLA2R1, SLC7A11, SLC13A4, SLC6A20, SLC17A6, SLC25A21, ANO5 |
| 4_Member | GO Biological Processes | GO:0098656 | anion transmembrane transport | -8.664912689 | -5.324 | 13/247 | CASR, GJA1, SLC4A1, SLC5A5, SLC6A13, SLC12A1, SLC22A6, SLC7A11, SLC13A4, SLC6A20, SLC17A6, SLC25A21, ANO5 |
| 4_Member | GO Biological Processes | GO:0046942 | carboxylic acid transport | -7.156320881 | -4.078 | 12/274 | CASR, EDN1, GJA1, SLC6A12, SLC6A13, SLC22A3, SLC22A6, PLA2R1, SLC7A11, SLC6A20, SLC17A6, SLC25A21 |
| 4_Member | GO Biological Processes | GO:0015711 | organic anion transport | -6.805513144 | -3.750 | 13/355 | CASR, EDN1, GJA1, SLC4A1, SLC6A12, SLC6A13, SLC22A3, SLC22A6, PLA2R1, SLC7A11, SLC6A20, SLC17A6, SLC25A21 |
| 4_Member | Reactome Gene Sets | R-HSA-425366 | Transport of bile salts and organic acids, metal ions and amine compounds | -6.207490024 | -3.313 | 7/85 | CP, SLC6A12, SLC6A13, SLC22A3, SLC22A6, SLC13A4, SLC6A20 |
| 4_Member | GO Biological Processes | GO:0015849 | organic acid transport | -5.773252511 | -3.007 | 11/305 | EDN1, GJA1, PPARG, SLC6A12, SLC6A13, SLC22A6, PLA2R1, SLC7A11, SLC6A20, SLC17A6, SLC25A21 |
| 4_Member | GO Biological Processes | GO:0003018 | vascular process in circulatory system | -5.498873524 | -2.824 | 10/263 | ACTA2, BMP6, CASR, EDN1, FOXC2, GJA1, SLC5A5, SLC6A13, SLC22A3, SLC6A20 |
| 4_Member | GO Biological Processes | GO:0072337 | modified amino acid transport | -4.36877192 | -2.069 | 4/34 | GJA1, SLC6A13, SLC7A11, SLC6A20 |
| 4_Member | GO Biological Processes | GO:0015718 | monocarboxylic acid transport | -4.34250482 | -2.050 | 6/111 | CASR, EDN1, SLC6A12, SLC6A13, SLC22A3, PLA2R1 |
| 4_Member | GO Biological Processes | GO:0006835 | dicarboxylic acid transport | -3.872741828 | -1.710 | 5/85 | GJA1, SLC22A6, SLC7A11, SLC17A6, SLC25A21 |
| 4_Member | GO Biological Processes | GO:0010232 | vascular transport | -3.80163325 | -1.675 | 5/88 | GJA1, SLC5A5, SLC6A13, SLC22A3, SLC6A20 |
| 4_Member | Reactome Gene Sets | R-HSA-442660 | Na+/Cl- dependent neurotransmitter transporters | -3.771501704 | -1.650 | 3/19 | SLC6A12, SLC6A13, SLC6A20 |
| 4_Member | GO Biological Processes | GO:0006865 | amino acid transport | -3.701714339 | -1.610 | 6/145 | GJA1, SLC6A12, SLC6A13, SLC7A11, SLC6A20, SLC17A6 |
| 4_Member | GO Biological Processes | GO:1905039 | carboxylic acid transmembrane transport | -3.63774955 | -1.564 | 6/149 | SLC6A12, SLC6A13, SLC7A11, SLC6A20, SLC17A6, SLC25A21 |
| 4_Member | GO Biological Processes | GO:1903825 | organic acid transmembrane transport | -3.622065812 | -1.553 | 6/150 | SLC6A12, SLC6A13, SLC7A11, SLC6A20, SLC17A6, SLC25A21 |
| 4_Member | GO Biological Processes | GO:0043090 | amino acid import | -3.605348445 | -1.548 | 4/53 | SLC6A12, SLC6A13, SLC7A11, SLC6A20 |
| 4_Member | GO Biological Processes | GO:0003333 | amino acid transmembrane transport | -3.582745553 | -1.535 | 5/98 | SLC6A12, SLC6A13, SLC7A11, SLC6A20, SLC17A6 |
| 4_Member | GO Biological Processes | GO:0072348 | sulfur compound transport | -3.573863067 | -1.533 | 4/54 | GJA1, SLC6A13, SLC7A11, SLC13A4 |
| 4_Member | GO Biological Processes | GO:0015800 | acidic amino acid transport | -3.512761633 | -1.484 | 4/56 | GJA1, SLC6A13, SLC7A11, SLC17A6 |
| 4_Member | Reactome Gene Sets | R-HSA-352230 | Amino acid transport across the plasma membrane | -3.046605772 | -1.145 | 3/33 | SLC6A12, SLC7A11, SLC6A20 |
| 4_Member | GO Biological Processes | GO:0098810 | neurotransmitter reuptake | -2.971227665 | -1.089 | 3/35 | SLC6A12, SLC6A13, SLC22A3 |
| 4_Member | GO Biological Processes | GO:0150104 | transport across blood-brain barrier | -2.790168858 | -0.971 | 4/87 | SLC5A5, SLC6A13, SLC22A3, SLC6A20 |
| 4_Member | GO Biological Processes | GO:0001504 | neurotransmitter uptake | -2.710396091 | -0.915 | 3/43 | SLC6A12, SLC6A13, SLC22A3 |
| 4_Member | GO Biological Processes | GO:0089718 | amino acid import across plasma membrane | -2.681551197 | -0.896 | 3/44 | SLC6A13, SLC7A11, SLC6A20 |
| 4_Member | GO Biological Processes | GO:0098657 | import into cell | -2.674794531 | -0.891 | 6/228 | SLC6A12, SLC6A13, SLC12A1, SLC22A3, SLC7A11, SLC6A20 |
| 4_Member | GO Biological Processes | GO:0042908 | xenobiotic transport | -2.473751672 | -0.748 | 3/52 | GJA1, SLC6A13, SLC22A3 |
| 4_Member | GO Biological Processes | GO:1902475 | L-alpha-amino acid transmembrane transport | -2.201475187 | -0.560 | 3/65 | SLC7A11, SLC6A20, SLC17A6 |
| 4_Member | GO Biological Processes | GO:0015807 | L-amino acid transport | -2.12972492 | -0.508 | 3/69 | SLC7A11, SLC6A20, SLC17A6 |
| 5_Summary | GO Biological Processes | GO:0001568 | blood vessel development | -8.402297645 | -5.125 | 21/771 | ACTA2, KLK3, BMP4, BMP7, C5, CMA1, COL1A1, COL1A2, CYP1B1, DLX3, EDN1, FOXC2, ITGA5, LOX, SMAD6, PF4, PPARG, ALDH1A2, FGFBP1, ESM1, CCR2 |
| 5_Member | GO Biological Processes | GO:0001568 | blood vessel development | -8.402297645 | -5.125 | 21/771 | ACTA2, KLK3, BMP4, BMP7, C5, CMA1, COL1A1, COL1A2, CYP1B1, DLX3, EDN1, FOXC2, ITGA5, LOX, SMAD6, PF4, PPARG, ALDH1A2, FGFBP1, ESM1, CCR2 |
| 5_Member | GO Biological Processes | GO:0001525 | angiogenesis | -4.32787467 | -2.043 | 13/600 | KLK3, BMP4, C5, CMA1, CYP1B1, EDN1, FOXC2, ITGA5, PF4, PPARG, FGFBP1, ESM1, CCR2 |
| 5_Member | GO Biological Processes | GO:0048514 | blood vessel morphogenesis | -4.293353526 | -2.016 | 14/693 | KLK3, BMP4, C5, CMA1, CYP1B1, EDN1, FOXC2, ITGA5, LOX, PF4, PPARG, FGFBP1, ESM1, CCR2 |
| 5_Member | GO Biological Processes | GO:0045765 | regulation of angiogenesis | -3.72731486 | -1.618 | 9/346 | KLK3, C5, CMA1, CYP1B1, FOXC2, ITGA5, PF4, PPARG, CCR2 |
| 5_Member | GO Biological Processes | GO:1901342 | regulation of vasculature development | -3.671999092 | -1.587 | 9/352 | KLK3, C5, CMA1, CYP1B1, FOXC2, ITGA5, PF4, PPARG, CCR2 |
| 5_Member | GO Biological Processes | GO:0045766 | positive regulation of angiogenesis | -2.395008619 | -0.691 | 5/181 | C5, CMA1, CYP1B1, FOXC2, ITGA5 |
| 5_Member | GO Biological Processes | GO:1904018 | positive regulation of vasculature development | -2.395008619 | -0.691 | 5/181 | C5, CMA1, CYP1B1, FOXC2, ITGA5 |
| 5_Member | GO Biological Processes | GO:0010574 | regulation of vascular endothelial growth factor production | -2.339713576 | -0.655 | 3/58 | C5, CYP1B1, CCR2 |
| 5_Member | GO Biological Processes | GO:0010573 | vascular endothelial growth factor production | -2.258594482 | -0.596 | 3/62 | C5, CYP1B1, CCR2 |
| 5_Member | GO Biological Processes | GO:0033627 | cell adhesion mediated by integrin | -2.0789 | -0.467 | 3/72 | CYP1B1, FOXC2, ITGA5 |
| 6_Summary | Reactome Gene Sets | R-HSA-1566948 | Elastic fibre formation | -8.160331221 | -4.917 | 7/45 | BMP4, BMP7, ELN, ITGA5, LOX, FBLN5, EMILIN3, SMAD6, EDN1, MDFI, WNT6 |
| 6_Member | Reactome Gene Sets | R-HSA-1566948 | Elastic fibre formation | -8.160331221 | -4.917 | 7/45 | BMP4, BMP7, ELN, ITGA5, LOX, FBLN5, EMILIN3 |
| 6_Member | Reactome Gene Sets | R-HSA-2129379 | Molecules associated with elastic fibres | -5.59348177 | -2.890 | 5/38 | BMP4, BMP7, ELN, FBLN5, EMILIN3 |
| 6_Member | GO Biological Processes | GO:0003180 | aortic valve morphogenesis | -3.086169134 | -1.165 | 3/32 | BMP4, ELN, SMAD6 |
| 6_Member | GO Biological Processes | GO:0003176 | aortic valve development | -2.900371524 | -1.046 | 3/37 | BMP4, ELN, SMAD6 |
| 6_Member | GO Biological Processes | GO:0009953 | dorsal/ventral pattern formation | -2.865706762 | -1.021 | 4/83 | BMP4, EDN1, SMAD6, MDFI |
| 6_Member | GO Biological Processes | GO:0009798 | axis specification | -2.790168858 | -0.971 | 4/87 | BMP4, SMAD6, MDFI, WNT6 |
| 6_Member | GO Biological Processes | GO:1905314 | semi-lunar valve development | -2.770339906 | -0.959 | 3/41 | BMP4, ELN, SMAD6 |
| 6_Member | GO Biological Processes | GO:0003179 | heart valve morphogenesis | -2.404719756 | -0.699 | 3/55 | BMP4, ELN, SMAD6 |
| 6_Member | GO Biological Processes | GO:0003170 | heart valve development | -2.201475187 | -0.560 | 3/65 | BMP4, ELN, SMAD6 |
| 7_Summary | Reactome Gene Sets | R-HSA-425407 | SLC-mediated transmembrane transport | -7.573853627 | -4.363 | 12/251 | CP, SLC4A1, SLC5A5, SLC6A12, SLC6A13, SLC12A1, SLC22A3, SLC22A6, SLC7A11, SLC13A4, SLC6A20, SLC17A6, ATP6V1B1, PLN, PSMA8, MLKL, ANO5, CASR, ENPP1 |
| 7_Member | Reactome Gene Sets | R-HSA-425407 | SLC-mediated transmembrane transport | -7.573853627 | -4.363 | 12/251 | CP, SLC4A1, SLC5A5, SLC6A12, SLC6A13, SLC12A1, SLC22A3, SLC22A6, SLC7A11, SLC13A4, SLC6A20, SLC17A6 |
| 7_Member | Reactome Gene Sets | R-HSA-382551 | Transport of small molecules | -5.941017477 | -3.109 | 17/729 | ATP6V1B1, CP, PLN, SLC4A1, SLC5A5, SLC6A12, SLC6A13, SLC12A1, SLC22A3, SLC22A6, SLC7A11, SLC13A4, SLC6A20, SLC17A6, PSMA8, MLKL, ANO5 |
| 7_Member | Reactome Gene Sets | R-HSA-425393 | Transport of inorganic cations/anions and amino acids/oligopeptides | -5.503535736 | -2.824 | 7/108 | SLC4A1, SLC5A5, SLC6A12, SLC12A1, SLC7A11, SLC6A20, SLC17A6 |
| 7_Member | GO Biological Processes | GO:0015698 | inorganic anion transport | -4.939454293 | -2.446 | 8/183 | CASR, ENPP1, SLC4A1, SLC5A5, SLC12A1, SLC22A6, SLC13A4, ANO5 |
| 7_Member | GO Biological Processes | GO:0098661 | inorganic anion transmembrane transport | -4.778378557 | -2.366 | 6/93 | CASR, SLC4A1, SLC5A5, SLC12A1, SLC13A4, ANO5 |
| 7_Member | GO Biological Processes | GO:0006814 | sodium ion transport | -4.038798853 | -1.815 | 8/245 | ATP6V1B1, SLC5A5, SLC6A12, SLC6A13, SLC12A1, SLC13A4, SLC6A20, SLC17A6 |
| 7_Member | Reactome Gene Sets | R-HSA-5619102 | SLC transporter disorders | -3.582745553 | -1.535 | 5/98 | CP, SLC4A1, SLC5A5, SLC12A1, SLC6A20 |
| 7_Member | WikiPathways | WP4917 | Proximal tubule transport | -3.454002851 | -1.439 | 4/58 | ATP6V1B1, SLC5A5, SLC22A6, SLC6A20 |
| 7_Member | Reactome Gene Sets | R-HSA-5619115 | Disorders of transmembrane transporters | -3.252081509 | -1.284 | 6/176 | CP, SLC4A1, SLC5A5, SLC12A1, SLC6A20, PSMA8 |
| 7_Member | GO Biological Processes | GO:1902476 | chloride transmembrane transport | -3.214562832 | -1.254 | 4/67 | CASR, SLC4A1, SLC12A1, ANO5 |
| 7_Member | GO Biological Processes | GO:0006821 | chloride transport | -2.420488846 | -0.708 | 4/110 | CASR, SLC4A1, SLC12A1, ANO5 |
| 8_Summary | GO Biological Processes | GO:0070848 | response to growth factor | -6.704071294 | -3.669 | 18/720 | BMP4, BMP6, BMP7, CASR, COL1A1, COL1A2, COL3A1, EDN1, FOXD1, FMOD, LRRC32, ITGA5, LOX, LUM, SMAD6, PPARG, FGFBP1, WFIKKN2 |
| 8_Member | GO Biological Processes | GO:0070848 | response to growth factor | -6.704071294 | -3.669 | 18/720 | BMP4, BMP6, BMP7, CASR, COL1A1, COL1A2, COL3A1, EDN1, FOXD1, FMOD, LRRC32, ITGA5, LOX, LUM, SMAD6, PPARG, FGFBP1, WFIKKN2 |
| 8_Member | GO Biological Processes | GO:0071363 | cellular response to growth factor stimulus | -6.276804689 | -3.351 | 17/689 | BMP4, BMP6, BMP7, CASR, COL1A1, COL1A2, COL3A1, EDN1, FOXD1, FMOD, LRRC32, ITGA5, LOX, SMAD6, PPARG, FGFBP1, WFIKKN2 |
| 8_Member | GO Biological Processes | GO:0007178 | transmembrane receptor protein serine/threonine kinase signaling pathway | -5.904832268 | -3.092 | 12/359 | BMP4, BMP6, BMP7, COL1A2, COL3A1, FOXD1, FMOD, LRRC32, LOX, SMAD6, PPARG, WFIKKN2 |
| 8_Member | GO Biological Processes | GO:0071560 | cellular response to transforming growth factor beta stimulus | -5.633621651 | -2.902 | 10/254 | COL1A1, COL1A2, COL3A1, EDN1, FMOD, LRRC32, LOX, SMAD6, PPARG, WFIKKN2 |
| 8_Member | GO Biological Processes | GO:0071559 | response to transforming growth factor beta | -5.54318612 | -2.849 | 10/260 | COL1A1, COL1A2, COL3A1, EDN1, FMOD, LRRC32, LOX, SMAD6, PPARG, WFIKKN2 |
| 8_Member | GO Biological Processes | GO:0007179 | transforming growth factor beta receptor signaling pathway | -4.629798732 | -2.264 | 8/202 | COL1A2, COL3A1, FMOD, LRRC32, LOX, SMAD6, PPARG, WFIKKN2 |
| 8_Member | GO Biological Processes | GO:0090287 | regulation of cellular response to growth factor stimulus | -3.402979452 | -1.402 | 8/304 | BMP4, FOXD1, ITGA5, LOX, SMAD6, PPARG, FGFBP1, WFIKKN2 |
| 8_Member | GO Biological Processes | GO:0017015 | regulation of transforming growth factor beta receptor signaling pathway | -2.17653889 | -0.543 | 4/129 | LOX, SMAD6, PPARG, WFIKKN2 |
| 8_Member | GO Biological Processes | GO:1903844 | regulation of cellular response to transforming growth factor beta stimulus | -2.141869589 | -0.517 | 4/132 | LOX, SMAD6, PPARG, WFIKKN2 |
| 9_Summary | GO Biological Processes | GO:0010817 | regulation of hormone levels | -6.585456093 | -3.571 | 15/506 | ALDH1A3, ASMT, BMP6, CASR, CMA1, CPA3, CRABP2, CYP1B1, EDN1, FOXD1, GJA1, NKX6-1, SLC5A5, AKR1C3, ALDH1A2, AOAH, ATP6V1B1, HAL, PPARG, SLC4A1, MTHFS, CYP39A1, BMP4, BMP7, SLC6A13, PLA2R1, ADCY10, NLRC4, GREB1, H3C13, H2AC19, CDH1, UPB1, GOT1L1, COL1A2, MLKL, LRRC32, SMAD6, NELL1, FGFBP1, CLDN19, LOX, SLC7A11 |
| 9_Member | GO Biological Processes | GO:0010817 | regulation of hormone levels | -6.585456093 | -3.571 | 15/506 | ALDH1A3, ASMT, BMP6, CASR, CMA1, CPA3, CRABP2, CYP1B1, EDN1, FOXD1, GJA1, NKX6-1, SLC5A5, AKR1C3, ALDH1A2 |
| 9_Member | GO Biological Processes | GO:0042445 | hormone metabolic process | -6.233737333 | -3.324 | 10/218 | ALDH1A3, ASMT, BMP6, CMA1, CPA3, CRABP2, CYP1B1, SLC5A5, AKR1C3, ALDH1A2 |
| 9_Member | GO Biological Processes | GO:0042574 | retinal metabolic process | -5.739936799 | -2.996 | 4/16 | ALDH1A3, CYP1B1, AKR1C3, ALDH1A2 |
| 9_Member | GO Biological Processes | GO:0002138 | retinoic acid biosynthetic process | -4.989414173 | -2.478 | 3/8 | ALDH1A3, AKR1C3, ALDH1A2 |
| 9_Member | GO Biological Processes | GO:0034754 | cellular hormone metabolic process | -4.822385366 | -2.385 | 7/137 | ALDH1A3, ASMT, BMP6, CRABP2, CYP1B1, AKR1C3, ALDH1A2 |
| 9_Member | GO Biological Processes | GO:0016102 | diterpenoid biosynthetic process | -4.815163773 | -2.383 | 3/9 | ALDH1A3, AKR1C3, ALDH1A2 |
| 9_Member | GO Biological Processes | GO:0006081 | cellular aldehyde metabolic process | -4.601118609 | -2.240 | 5/60 | ALDH1A3, BMP6, CYP1B1, AKR1C3, ALDH1A2 |
| 9_Member | GO Biological Processes | GO:0042573 | retinoic acid metabolic process | -4.36877192 | -2.069 | 4/34 | ALDH1A3, CRABP2, AKR1C3, ALDH1A2 |
| 9_Member | GO Biological Processes | GO:0032787 | monocarboxylic acid metabolic process | -4.364889946 | -2.069 | 13/595 | ALDH1A3, AOAH, ATP6V1B1, CRABP2, CYP1B1, EDN1, HAL, PPARG, SLC4A1, AKR1C3, ALDH1A2, MTHFS, CYP39A1 |
| 9_Member | GO Biological Processes | GO:0016114 | terpenoid biosynthetic process | -4.092466842 | -1.853 | 3/15 | ALDH1A3, AKR1C3, ALDH1A2 |
| 9_Member | GO Biological Processes | GO:0001523 | retinoid metabolic process | -3.972006543 | -1.764 | 5/81 | ALDH1A3, CRABP2, CYP1B1, AKR1C3, ALDH1A2 |
| 9_Member | Reactome Gene Sets | R-HSA-5362517 | Signaling by Retinoic Acid | -3.96120439 | -1.757 | 4/43 | ALDH1A3, CRABP2, AKR1C3, ALDH1A2 |
| 9_Member | GO Biological Processes | GO:0016101 | diterpenoid metabolic process | -3.825033118 | -1.683 | 5/87 | ALDH1A3, CRABP2, CYP1B1, AKR1C3, ALDH1A2 |
| 9_Member | GO Biological Processes | GO:0042572 | retinol metabolic process | -3.703845548 | -1.610 | 4/50 | ALDH1A3, CYP1B1, AKR1C3, ALDH1A2 |
| 9_Member | GO Biological Processes | GO:0006721 | terpenoid metabolic process | -3.603486318 | -1.548 | 5/97 | ALDH1A3, CRABP2, CYP1B1, AKR1C3, ALDH1A2 |
| 9_Member | GO Biological Processes | GO:0034308 | primary alcohol metabolic process | -3.582745553 | -1.535 | 5/98 | ALDH1A3, BMP6, CYP1B1, AKR1C3, ALDH1A2 |
| 9_Member | Reactome Gene Sets | R-HSA-5365859 | RA biosynthesis pathway | -3.575810156 | -1.533 | 3/22 | ALDH1A3, AKR1C3, ALDH1A2 |
| 9_Member | GO Biological Processes | GO:0008299 | isoprenoid biosynthetic process | -3.258984759 | -1.288 | 3/28 | ALDH1A3, AKR1C3, ALDH1A2 |
| 9_Member | GO Biological Processes | GO:0006720 | isoprenoid metabolic process | -3.24551062 | -1.280 | 5/116 | ALDH1A3, CRABP2, CYP1B1, AKR1C3, ALDH1A2 |
| 9_Member | GO Biological Processes | GO:0120254 | olefinic compound metabolic process | -3.162272931 | -1.220 | 5/121 | ALDH1A3, BMP6, CYP1B1, AKR1C3, ALDH1A2 |
| 9_Member | GO Biological Processes | GO:0010942 | positive regulation of cell death | -3.02666412 | -1.126 | 11/618 | ALDH1A3, BMP4, BMP7, CYP1B1, PPARG, SLC6A13, AKR1C3, ALDH1A2, PLA2R1, ADCY10, NLRC4 |
| 9_Member | GO Biological Processes | GO:0043065 | positive regulation of apoptotic process | -2.929852539 | -1.063 | 10/539 | ALDH1A3, BMP4, BMP7, CYP1B1, PPARG, AKR1C3, ALDH1A2, PLA2R1, ADCY10, NLRC4 |
| 9_Member | GO Biological Processes | GO:0043068 | positive regulation of programmed cell death | -2.835591204 | -1.000 | 10/555 | ALDH1A3, BMP4, BMP7, CYP1B1, PPARG, AKR1C3, ALDH1A2, PLA2R1, ADCY10, NLRC4 |
| 9_Member | Reactome Gene Sets | R-HSA-9006931 | Signaling by Nuclear Receptors | -2.748675252 | -0.941 | 7/299 | ALDH1A3, CRABP2, AKR1C3, ALDH1A2, GREB1, H3C13, H2AC19 |
| 9_Member | GO Biological Processes | GO:0021983 | pituitary gland development | -2.710396091 | -0.915 | 3/43 | BMP4, CDH1, ALDH1A2 |
| 9_Member | WikiPathways | WP716 | Vitamin A and carotenoid metabolism | -2.681551197 | -0.896 | 3/44 | ALDH1A3, CRABP2, ALDH1A2 |
| 9_Member | GO Biological Processes | GO:0046394 | carboxylic acid biosynthetic process | -2.621602975 | -0.856 | 7/315 | ALDH1A3, EDN1, AKR1C3, ALDH1A2, CYP39A1, UPB1, GOT1L1 |
| 9_Member | GO Biological Processes | GO:0016053 | organic acid biosynthetic process | -2.606295946 | -0.845 | 7/317 | ALDH1A3, EDN1, AKR1C3, ALDH1A2, CYP39A1, UPB1, GOT1L1 |
| 9_Member | GO Biological Processes | GO:0051259 | protein complex oligomerization | -2.582018307 | -0.828 | 6/238 | ALDH1A3, COL1A2, ALDH1A2, UPB1, NLRC4, MLKL |
| 9_Member | GO Biological Processes | GO:0051260 | protein homooligomerization | -2.384891841 | -0.685 | 5/182 | ALDH1A3, ALDH1A2, UPB1, NLRC4, MLKL |
| 9_Member | GO Biological Processes | GO:0051289 | protein homotetramerization | -2.360961922 | -0.671 | 3/57 | ALDH1A3, ALDH1A2, UPB1 |
| 9_Member | GO Biological Processes | GO:0008285 | negative regulation of cell population proliferation | -2.254068983 | -0.593 | 11/779 | BMP4, BMP7, CYP1B1, LRRC32, GJA1, SMAD6, NELL1, PPARG, ALDH1A2, FGFBP1, CLDN19 |
| 9_Member | GO Biological Processes | GO:0060541 | respiratory system development | -2.178005798 | -0.543 | 5/204 | ALDH1A3, BMP4, LOX, ALDH1A2, SLC7A11 |
| 9_Member | GO Biological Processes | GO:0072330 | monocarboxylic acid biosynthetic process | -2.09281793 | -0.478 | 5/214 | ALDH1A3, EDN1, AKR1C3, ALDH1A2, CYP39A1 |
| 10_Summary | GO Biological Processes | GO:0007507 | heart development | -6.425108531 | -3.466 | 16/595 | BMP4, BMP7, COL3A1, EDN1, ELN, FOXC2, GJA1, LOX, SMAD6, MYH11, PLN, ALDH1A2, TBX18, HSPB7, ADGRG6, POPDC2, WFIKKN2, TAGLN, ALX4, IL7, PPARG, WNT6 |
| 10_Member | GO Biological Processes | GO:0007507 | heart development | -6.425108531 | -3.466 | 16/595 | BMP4, BMP7, COL3A1, EDN1, ELN, FOXC2, GJA1, LOX, SMAD6, MYH11, PLN, ALDH1A2, TBX18, HSPB7, ADGRG6, POPDC2 |
| 10_Member | GO Biological Processes | GO:0014706 | striated muscle tissue development | -5.602209365 | -2.890 | 12/384 | BMP4, BMP7, EDN1, ELN, FOXC2, GJA1, LOX, MYH11, PLN, ALDH1A2, TBX18, POPDC2 |
| 10_Member | GO Biological Processes | GO:0060537 | muscle tissue development | -5.387609052 | -2.747 | 12/403 | BMP4, BMP7, EDN1, ELN, FOXC2, GJA1, LOX, MYH11, PLN, ALDH1A2, TBX18, POPDC2 |
| 10_Member | GO Biological Processes | GO:0048738 | cardiac muscle tissue development | -4.996936011 | -2.478 | 9/237 | BMP4, BMP7, EDN1, FOXC2, GJA1, MYH11, PLN, ALDH1A2, TBX18 |
| 10_Member | GO Biological Processes | GO:0072189 | ureter development | -3.84429923 | -1.694 | 3/18 | BMP4, ALDH1A2, TBX18 |
| 10_Member | GO Biological Processes | GO:0055001 | muscle cell development | -3.175722079 | -1.225 | 6/182 | BMP4, EDN1, LOX, MYH11, TBX18, WFIKKN2 |
| 10_Member | GO Biological Processes | GO:0061061 | muscle structure development | -2.926657641 | -1.061 | 11/636 | BMP4, EDN1, ELN, FOXC2, LOX, MYH11, TAGLN, TBX18, ALX4, POPDC2, WFIKKN2 |
| 10_Member | GO Biological Processes | GO:0003161 | cardiac conduction system development | -2.900371524 | -1.046 | 3/37 | BMP4, GJA1, TBX18 |
| 10_Member | GO Biological Processes | GO:0055013 | cardiac muscle cell development | -2.827439774 | -0.996 | 4/85 | BMP4, EDN1, MYH11, TBX18 |
| 10_Member | GO Biological Processes | GO:0055006 | cardiac cell development | -2.718433743 | -0.918 | 4/91 | BMP4, EDN1, MYH11, TBX18 |
| 10_Member | GO Biological Processes | GO:0035051 | cardiocyte differentiation | -2.66000724 | -0.884 | 5/157 | BMP4, BMP7, EDN1, MYH11, TBX18 |
| 10_Member | GO Biological Processes | GO:0035282 | segmentation | -2.617243826 | -0.854 | 4/97 | BMP4, FOXC2, ALDH1A2, TBX18 |
| 10_Member | GO Biological Processes | GO:0007517 | muscle organ development | -2.531571209 | -0.791 | 7/327 | BMP4, ELN, FOXC2, LOX, TAGLN, ALX4, POPDC2 |
| 10_Member | GO Biological Processes | GO:0045165 | cell fate commitment | -2.393871196 | -0.691 | 6/260 | BMP4, FOXC2, IL7, PPARG, WNT6, TBX18 |
| 10_Member | GO Biological Processes | GO:0055007 | cardiac muscle cell differentiation | -2.224264395 | -0.573 | 4/125 | BMP4, EDN1, MYH11, TBX18 |
| 10_Member | GO Biological Processes | GO:0001756 | somitogenesis | -2.220183422 | -0.573 | 3/64 | FOXC2, ALDH1A2, TBX18 |
| 10_Member | GO Biological Processes | GO:0009952 | anterior/posterior pattern specification | -2.195662565 | -0.555 | 5/202 | BMP4, FOXC2, ALDH1A2, TBX18, ALX4 |
| 10_Member | GO Biological Processes | GO:0042692 | muscle cell differentiation | -2.155937628 | -0.527 | 7/384 | BMP4, EDN1, LOX, MYH11, TBX18, POPDC2, WFIKKN2 |
| 11_Summary | GO Biological Processes | GO:0048598 | embryonic morphogenesis | -6.425108531 | -3.466 | 16/595 | ALDH1A3, ALX3, ATP6V1B1, BMP4, BMP7, CRABP2, EDN1, FOXC2, GJA1, ITGA5, MDFI, MYO7A, WNT6, ALDH1A2, TBX18, ALX4, BMP6, CYP1B1, GJB2, SLC7A11, SLC17A6, ACTA2, FOXD1, SMAD6, NPHS2, COL1A1, MGP, COL1A2, DLX3, NKX6-1, PPARG, TAGLN, AKR1C3, IL31RA, CASR, ELN, ADGRG6, C5, TRIM5, LOX, PSMA8 |
| 11_Member | GO Biological Processes | GO:0048598 | embryonic morphogenesis | -6.425108531 | -3.466 | 16/595 | ALDH1A3, ALX3, ATP6V1B1, BMP4, BMP7, CRABP2, EDN1, FOXC2, GJA1, ITGA5, MDFI, MYO7A, WNT6, ALDH1A2, TBX18, ALX4 |
| 11_Member | GO Biological Processes | GO:0007423 | sensory organ development | -6.080247693 | -3.215 | 15/555 | ALDH1A3, ATP6V1B1, BMP4, BMP6, BMP7, CYP1B1, EDN1, FOXC2, GJB2, MYO7A, WNT6, ALDH1A2, TBX18, SLC7A11, SLC17A6 |
| 11_Member | GO Biological Processes | GO:0048562 | embryonic organ morphogenesis | -5.986263659 | -3.134 | 11/290 | ALDH1A3, ALX3, ATP6V1B1, BMP4, BMP7, EDN1, FOXC2, MDFI, MYO7A, TBX18, ALX4 |
| 11_Member | GO Biological Processes | GO:0072001 | renal system development | -5.800936894 | -3.023 | 11/303 | ACTA2, BMP4, BMP6, BMP7, FOXD1, FOXC2, SMAD6, WNT6, NPHS2, ALDH1A2, TBX18 |
| 11_Member | GO Biological Processes | GO:0072109 | glomerular mesangium development | -5.625381539 | -2.902 | 4/17 | ACTA2, BMP4, BMP7, FOXC2 |
| 11_Member | GO Biological Processes | GO:0001655 | urogenital system development | -5.332968833 | -2.708 | 11/339 | ACTA2, BMP4, BMP6, BMP7, FOXD1, FOXC2, SMAD6, WNT6, NPHS2, ALDH1A2, TBX18 |
| 11_Member | GO Biological Processes | GO:0048705 | skeletal system morphogenesis | -5.241133676 | -2.648 | 9/221 | ALX3, BMP4, BMP6, BMP7, COL1A1, FOXC2, MDFI, MGP, ALX4 |
| 11_Member | GO Biological Processes | GO:0048706 | embryonic skeletal system development | -5.175691509 | -2.604 | 7/121 | ALX3, BMP4, BMP7, COL1A1, FOXC2, MDFI, ALX4 |
| 11_Member | GO Biological Processes | GO:0001822 | kidney development | -5.072949791 | -2.522 | 10/294 | ACTA2, BMP4, BMP6, BMP7, FOXD1, FOXC2, SMAD6, WNT6, NPHS2, ALDH1A2 |
| 11_Member | GO Biological Processes | GO:0048568 | embryonic organ development | -5.072607838 | -2.522 | 12/433 | ALDH1A3, ALX3, ATP6V1B1, BMP4, BMP7, EDN1, FOXC2, MDFI, MYO7A, ALDH1A2, TBX18, ALX4 |
| 11_Member | GO Biological Processes | GO:0007389 | pattern specification process | -5.012808376 | -2.482 | 12/439 | ALX3, BMP4, BMP7, EDN1, FOXD1, FOXC2, SMAD6, MDFI, WNT6, ALDH1A2, TBX18, ALX4 |
| 11_Member | GO Biological Processes | GO:0001654 | eye development | -4.953296523 | -2.450 | 11/372 | ALDH1A3, BMP4, BMP6, BMP7, CYP1B1, FOXC2, MYO7A, WNT6, ALDH1A2, SLC7A11, SLC17A6 |
| 11_Member | GO Biological Processes | GO:0042476 | odontogenesis | -4.949178328 | -2.450 | 7/131 | BMP4, BMP7, COL1A1, COL1A2, DLX3, EDN1, WNT6 |
| 11_Member | GO Biological Processes | GO:0150063 | visual system development | -4.910047424 | -2.428 | 11/376 | ALDH1A3, BMP4, BMP6, BMP7, CYP1B1, FOXC2, MYO7A, WNT6, ALDH1A2, SLC7A11, SLC17A6 |
| 11_Member | GO Biological Processes | GO:0048880 | sensory system development | -4.846210746 | -2.395 | 11/382 | ALDH1A3, BMP4, BMP6, BMP7, CYP1B1, FOXC2, MYO7A, WNT6, ALDH1A2, SLC7A11, SLC17A6 |
| 11_Member | GO Biological Processes | GO:0072012 | glomerulus vasculature development | -4.844855854 | -2.395 | 4/26 | ACTA2, BMP4, BMP7, FOXC2 |
| 11_Member | GO Biological Processes | GO:0072073 | kidney epithelium development | -4.843078982 | -2.395 | 7/136 | BMP4, BMP7, FOXD1, FOXC2, SMAD6, WNT6, NPHS2 |
| 11_Member | GO Biological Processes | GO:0001657 | ureteric bud development | -4.832522338 | -2.389 | 6/91 | BMP4, BMP7, FOXD1, FOXC2, SMAD6, WNT6 |
| 11_Member | GO Biological Processes | GO:0072163 | mesonephric epithelium development | -4.805287688 | -2.383 | 6/92 | BMP4, BMP7, FOXD1, FOXC2, SMAD6, WNT6 |
| 11_Member | GO Biological Processes | GO:0072164 | mesonephric tubule development | -4.805287688 | -2.383 | 6/92 | BMP4, BMP7, FOXD1, FOXC2, SMAD6, WNT6 |
| 11_Member | GO Biological Processes | GO:0048704 | embryonic skeletal system morphogenesis | -4.75178778 | -2.349 | 6/94 | ALX3, BMP4, BMP7, FOXC2, MDFI, ALX4 |
| 11_Member | GO Biological Processes | GO:0061437 | renal system vasculature development | -4.712167097 | -2.320 | 4/28 | ACTA2, BMP4, BMP7, FOXC2 |
| 11_Member | GO Biological Processes | GO:0061440 | kidney vasculature development | -4.712167097 | -2.320 | 4/28 | ACTA2, BMP4, BMP7, FOXC2 |
| 11_Member | GO Biological Processes | GO:0072006 | nephron development | -4.701693427 | -2.320 | 7/143 | ACTA2, BMP4, BMP7, FOXD1, FOXC2, WNT6, NPHS2 |
| 11_Member | GO Biological Processes | GO:0001823 | mesonephros development | -4.699533784 | -2.320 | 6/96 | BMP4, BMP7, FOXD1, FOXC2, SMAD6, WNT6 |
| 11_Member | GO Biological Processes | GO:0030855 | epithelial cell differentiation | -4.64592916 | -2.276 | 14/643 | ACTA2, BMP4, BMP6, BMP7, DLX3, FOXC2, MYO7A, NKX6-1, PPARG, TAGLN, NPHS2, AKR1C3, SLC7A11, IL31RA |
| 11_Member | GO Biological Processes | GO:0035115 | embryonic forelimb morphogenesis | -4.531401815 | -2.187 | 4/31 | ALX3, CRABP2, ALDH1A2, ALX4 |
| 11_Member | GO Biological Processes | GO:0048754 | branching morphogenesis of an epithelial tube | -4.530990168 | -2.187 | 7/152 | BMP4, BMP7, CASR, EDN1, FOXD1, FOXC2, WNT6 |
| 11_Member | GO Biological Processes | GO:0032835 | glomerulus development | -4.497579439 | -2.166 | 5/63 | ACTA2, BMP4, BMP7, FOXC2, NPHS2 |
| 11_Member | GO Biological Processes | GO:0002009 | morphogenesis of an epithelium | -4.382405879 | -2.071 | 12/509 | ALDH1A3, BMP4, BMP7, CASR, EDN1, FOXD1, FOXC2, GJA1, ITGA5, WNT6, ALDH1A2, TBX18 |
| 11_Member | GO Biological Processes | GO:0030326 | embryonic limb morphogenesis | -4.214523722 | -1.951 | 6/117 | ALX3, BMP4, BMP7, CRABP2, ALDH1A2, ALX4 |
| 11_Member | GO Biological Processes | GO:0035113 | embryonic appendage morphogenesis | -4.214523722 | -1.951 | 6/117 | ALX3, BMP4, BMP7, CRABP2, ALDH1A2, ALX4 |
| 11_Member | GO Biological Processes | GO:0048729 | tissue morphogenesis | -4.197882688 | -1.938 | 13/618 | ACTA2, ALDH1A3, BMP4, BMP7, CASR, EDN1, FOXD1, FOXC2, GJA1, ITGA5, WNT6, ALDH1A2, TBX18 |
| 11_Member | GO Biological Processes | GO:0035136 | forelimb morphogenesis | -4.174696949 | -1.924 | 4/38 | ALX3, CRABP2, ALDH1A2, ALX4 |
| 11_Member | GO Biological Processes | GO:0048736 | appendage development | -4.173778554 | -1.924 | 7/173 | ALX3, BMP4, BMP7, CRABP2, ALDH1A2, SLC7A11, ALX4 |
| 11_Member | GO Biological Processes | GO:0060173 | limb development | -4.173778554 | -1.924 | 7/173 | ALX3, BMP4, BMP7, CRABP2, ALDH1A2, SLC7A11, ALX4 |
| 11_Member | GO Biological Processes | GO:0003151 | outflow tract morphogenesis | -4.13154579 | -1.885 | 5/75 | BMP4, BMP7, ELN, FOXC2, SMAD6 |
| 11_Member | GO Biological Processes | GO:0061138 | morphogenesis of a branching epithelium | -4.020776685 | -1.804 | 7/183 | BMP4, BMP7, CASR, EDN1, FOXD1, FOXC2, WNT6 |
| 11_Member | GO Biological Processes | GO:0043010 | camera-type eye development | -3.950871897 | -1.757 | 9/323 | ALDH1A3, BMP4, BMP7, CYP1B1, FOXC2, WNT6, ALDH1A2, SLC7A11, SLC17A6 |
| 11_Member | GO Biological Processes | GO:0014032 | neural crest cell development | -3.946689522 | -1.757 | 5/82 | BMP4, BMP7, EDN1, FOXC2, ALDH1A2 |
| 11_Member | GO Biological Processes | GO:0001656 | metanephros development | -3.872741828 | -1.710 | 5/85 | BMP4, BMP7, FOXD1, FOXC2, NPHS2 |
| 11_Member | GO Biological Processes | GO:0003002 | regionalization | -3.870953246 | -1.710 | 9/331 | BMP4, EDN1, FOXD1, FOXC2, SMAD6, MDFI, ALDH1A2, TBX18, ALX4 |
| 11_Member | GO Biological Processes | GO:0014031 | mesenchymal cell development | -3.825033118 | -1.683 | 5/87 | BMP4, BMP7, EDN1, FOXC2, ALDH1A2 |
| 11_Member | GO Biological Processes | GO:0048864 | stem cell development | -3.825033118 | -1.683 | 5/87 | BMP4, BMP7, EDN1, FOXC2, ALDH1A2 |
| 11_Member | GO Biological Processes | GO:0001763 | morphogenesis of a branching structure | -3.822125229 | -1.683 | 7/197 | BMP4, BMP7, CASR, EDN1, FOXD1, FOXC2, WNT6 |
| 11_Member | GO Biological Processes | GO:0035850 | epithelial cell differentiation involved in kidney development | -3.809009779 | -1.675 | 4/47 | ACTA2, BMP4, FOXC2, NPHS2 |
| 11_Member | GO Biological Processes | GO:0035107 | appendage morphogenesis | -3.801546467 | -1.675 | 6/139 | ALX3, BMP4, BMP7, CRABP2, ALDH1A2, ALX4 |
| 11_Member | GO Biological Processes | GO:0035108 | limb morphogenesis | -3.801546467 | -1.675 | 6/139 | ALX3, BMP4, BMP7, CRABP2, ALDH1A2, ALX4 |
| 11_Member | GO Biological Processes | GO:0072074 | kidney mesenchyme development | -3.771501704 | -1.650 | 3/19 | BMP4, BMP7, FOXD1 |
| 11_Member | GO Biological Processes | GO:0014033 | neural crest cell differentiation | -3.710903937 | -1.610 | 5/92 | BMP4, BMP7, EDN1, FOXC2, ALDH1A2 |
| 11_Member | GO Biological Processes | GO:0061318 | renal filtration cell differentiation | -3.702756243 | -1.610 | 3/20 | BMP4, FOXC2, NPHS2 |
| 11_Member | GO Biological Processes | GO:0072112 | glomerular visceral epithelial cell differentiation | -3.702756243 | -1.610 | 3/20 | BMP4, FOXC2, NPHS2 |
| 11_Member | GO Biological Processes | GO:0016331 | morphogenesis of embryonic epithelium | -3.653554578 | -1.571 | 6/148 | ALDH1A3, BMP4, BMP7, WNT6, ALDH1A2, TBX18 |
| 11_Member | GO Biological Processes | GO:0072311 | glomerular epithelial cell differentiation | -3.637644585 | -1.564 | 3/21 | BMP4, FOXC2, NPHS2 |
| 11_Member | GO Biological Processes | GO:0072132 | mesenchyme morphogenesis | -3.5430086 | -1.508 | 4/55 | ACTA2, BMP4, BMP7, FOXC2 |
| 11_Member | GO Biological Processes | GO:0060485 | mesenchyme development | -3.519455103 | -1.487 | 8/292 | ACTA2, BMP4, BMP7, COL1A1, EDN1, FOXD1, FOXC2, ALDH1A2 |
| 11_Member | GO Biological Processes | GO:0061005 | cell differentiation involved in kidney development | -3.483100032 | -1.463 | 4/57 | ACTA2, BMP4, FOXC2, NPHS2 |
| 11_Member | GO Biological Processes | GO:0002064 | epithelial cell development | -3.470311018 | -1.452 | 7/225 | ACTA2, BMP4, BMP6, FOXC2, MYO7A, NKX6-1, NPHS2 |
| 11_Member | GO Biological Processes | GO:0072010 | glomerular epithelium development | -3.460788226 | -1.444 | 3/24 | BMP4, FOXC2, NPHS2 |
| 11_Member | GO Biological Processes | GO:0060561 | apoptotic process involved in morphogenesis | -3.407104103 | -1.402 | 3/25 | BMP4, BMP7, FOXC2 |
| 11_Member | GO Biological Processes | GO:0072009 | nephron epithelium development | -3.369147139 | -1.372 | 5/109 | BMP4, FOXD1, FOXC2, WNT6, NPHS2 |
| 11_Member | GO Biological Processes | GO:0035116 | embryonic hindlimb morphogenesis | -3.306371028 | -1.319 | 3/27 | ALX3, BMP4, ALX4 |
| 11_Member | GO Biological Processes | GO:0003007 | heart morphogenesis | -3.187435944 | -1.234 | 7/251 | BMP4, BMP7, ELN, FOXC2, SMAD6, ALDH1A2, ADGRG6 |
| 11_Member | GO Biological Processes | GO:0001704 | formation of primary germ layer | -3.162272931 | -1.220 | 5/121 | BMP4, BMP7, FOXC2, GJA1, ITGA5 |
| 11_Member | GO Biological Processes | GO:0003206 | cardiac chamber morphogenesis | -3.162272931 | -1.220 | 5/121 | BMP4, BMP7, FOXC2, SMAD6, ADGRG6 |
| 11_Member | GO Biological Processes | GO:0001707 | mesoderm formation | -3.096402124 | -1.171 | 4/72 | BMP4, BMP7, FOXC2, GJA1 |
| 11_Member | GO Biological Processes | GO:0043009 | chordate embryonic development | -3.055161746 | -1.145 | 11/613 | ALX3, BMP4, BMP7, C5, COL1A1, EDN1, FOXC2, MDFI, ALDH1A2, TBX18, ALX4 |
| 11_Member | GO Biological Processes | GO:0048332 | mesoderm morphogenesis | -3.051658391 | -1.145 | 4/74 | BMP4, BMP7, FOXC2, GJA1 |
| 11_Member | GO Biological Processes | GO:0048333 | mesodermal cell differentiation | -3.046605772 | -1.145 | 3/33 | BMP4, FOXC2, GJA1 |
| 11_Member | GO Biological Processes | GO:0035137 | hindlimb morphogenesis | -3.008317384 | -1.114 | 3/34 | ALX3, BMP4, ALX4 |
| 11_Member | GO Biological Processes | GO:0072028 | nephron morphogenesis | -3.008236741 | -1.114 | 4/76 | BMP4, BMP7, FOXD1, WNT6 |
| 11_Member | GO Biological Processes | GO:0009792 | embryo development ending in birth or egg hatching | -2.943050444 | -1.070 | 11/633 | ALX3, BMP4, BMP7, C5, COL1A1, EDN1, FOXC2, MDFI, ALDH1A2, TBX18, ALX4 |
| 11_Member | GO Biological Processes | GO:0072210 | metanephric nephron development | -2.801514761 | -0.977 | 3/40 | BMP4, FOXD1, NPHS2 |
| 11_Member | GO Biological Processes | GO:1902742 | apoptotic process involved in development | -2.801514761 | -0.977 | 3/40 | BMP4, BMP7, FOXC2 |
| 11_Member | GO Biological Processes | GO:0042475 | odontogenesis of dentin-containing tooth | -2.718433743 | -0.918 | 4/91 | BMP4, BMP7, DLX3, WNT6 |
| 11_Member | WikiPathways | WP4823 | Genes controlling nephrogenesis | -2.681551197 | -0.896 | 3/44 | FOXD1, FOXC2, NPHS2 |
| 11_Member | GO Biological Processes | GO:0060993 | kidney morphogenesis | -2.66692583 | -0.886 | 4/94 | BMP4, BMP7, FOXD1, WNT6 |
| 11_Member | WikiPathways | WP2857 | Mesodermal commitment pathway | -2.66000724 | -0.884 | 5/157 | BMP4, BMP7, FOXC2, SMAD6, TRIM5 |
| 11_Member | GO Biological Processes | GO:0048701 | embryonic cranial skeleton morphogenesis | -2.625944967 | -0.859 | 3/46 | ALX3, BMP4, FOXC2 |
| 11_Member | GO Biological Processes | GO:0003205 | cardiac chamber development | -2.612624423 | -0.850 | 5/161 | BMP4, BMP7, FOXC2, SMAD6, ADGRG6 |
| 11_Member | GO Biological Processes | GO:0048762 | mesenchymal cell differentiation | -2.582018307 | -0.828 | 6/238 | BMP4, BMP7, COL1A1, EDN1, FOXC2, ALDH1A2 |
| 11_Member | GO Biological Processes | GO:0060840 | artery development | -2.569274509 | -0.817 | 4/100 | BMP4, FOXC2, LOX, SMAD6 |
| 11_Member | GO Biological Processes | GO:0060562 | epithelial tube morphogenesis | -2.50972392 | -0.775 | 7/330 | BMP4, BMP7, CASR, EDN1, FOXD1, FOXC2, WNT6 |
| 11_Member | GO Biological Processes | GO:0001658 | branching involved in ureteric bud morphogenesis | -2.382625559 | -0.685 | 3/56 | BMP4, FOXD1, WNT6 |
| 11_Member | GO Biological Processes | GO:0007369 | gastrulation | -2.345085096 | -0.657 | 5/186 | BMP4, BMP7, FOXC2, GJA1, ITGA5 |
| 11_Member | GO Biological Processes | GO:0060675 | ureteric bud morphogenesis | -2.258594482 | -0.596 | 3/62 | BMP4, FOXD1, WNT6 |
| 11_Member | GO Biological Processes | GO:0072171 | mesonephric tubule morphogenesis | -2.239219625 | -0.584 | 3/63 | BMP4, FOXD1, WNT6 |
| 11_Member | GO Biological Processes | GO:2000242 | negative regulation of reproductive process | -2.239219625 | -0.584 | 3/63 | BMP4, BMP7, GJA1 |
| 11_Member | GO Biological Processes | GO:1905330 | regulation of morphogenesis of an epithelium | -2.201475187 | -0.560 | 3/65 | BMP4, BMP7, GJA1 |
| 11_Member | GO Biological Processes | GO:0007498 | mesoderm development | -2.17653889 | -0.543 | 4/129 | BMP4, BMP7, FOXC2, GJA1 |
| 11_Member | GO Biological Processes | GO:0048863 | stem cell differentiation | -2.151916511 | -0.524 | 5/207 | BMP4, BMP7, EDN1, FOXC2, ALDH1A2 |
| 11_Member | GO Biological Processes | GO:1904888 | cranial skeletal system development | -2.147218672 | -0.522 | 3/68 | ALX3, BMP4, FOXC2 |
| 11_Member | GO Biological Processes | GO:0072078 | nephron tubule morphogenesis | -2.095573529 | -0.480 | 3/71 | BMP4, FOXD1, WNT6 |
| 11_Member | GO Biological Processes | GO:0051783 | regulation of nuclear division | -2.064463516 | -0.456 | 4/139 | BMP4, BMP7, EDN1, PSMA8 |
| 11_Member | GO Biological Processes | GO:0072088 | nephron epithelium morphogenesis | -2.062484548 | -0.455 | 3/73 | BMP4, FOXD1, WNT6 |
| 11_Member | GO Biological Processes | GO:0003158 | endothelium development | -2.043192259 | -0.438 | 4/141 | BMP4, BMP6, FOXC2, GJA1 |
| 11_Member | GO Biological Processes | GO:0061333 | renal tubule morphogenesis | -2.030399611 | -0.429 | 3/75 | BMP4, FOXD1, WNT6 |
| 12_Summary | GO Biological Processes | GO:0009611 | response to wounding | -6.109579181 | -3.230 | 15/552 | SERPING1, COL1A1, COL3A1, EDN1, FOXC2, GJA1, ITGA5, LOX, PF4, SLC4A1, VWF, LYVE1, SLC7A11, CLDN19, CCR2, ATP6V1B1 |
| 12_Member | GO Biological Processes | GO:0009611 | response to wounding | -6.109579181 | -3.230 | 15/552 | SERPING1, COL1A1, COL3A1, EDN1, FOXC2, GJA1, ITGA5, LOX, PF4, SLC4A1, VWF, LYVE1, SLC7A11, CLDN19, CCR2 |
| 12_Member | GO Biological Processes | GO:0042060 | wound healing | -5.934751125 | -3.109 | 13/424 | SERPING1, COL1A1, COL3A1, EDN1, FOXC2, GJA1, ITGA5, LOX, PF4, SLC4A1, VWF, SLC7A11, CLDN19 |
| 12_Member | GO Biological Processes | GO:0007596 | blood coagulation | -3.553209749 | -1.514 | 7/218 | SERPING1, COL3A1, EDN1, PF4, SLC4A1, VWF, SLC7A11 |
| 12_Member | GO Biological Processes | GO:0007599 | hemostasis | -3.493679981 | -1.469 | 7/223 | SERPING1, COL3A1, EDN1, PF4, SLC4A1, VWF, SLC7A11 |
| 12_Member | GO Biological Processes | GO:0050817 | coagulation | -3.493679981 | -1.469 | 7/223 | SERPING1, COL3A1, EDN1, PF4, SLC4A1, VWF, SLC7A11 |
| 12_Member | GO Biological Processes | GO:0050878 | regulation of body fluid levels | -3.412060325 | -1.403 | 9/382 | ATP6V1B1, SERPING1, COL3A1, EDN1, GJA1, PF4, SLC4A1, VWF, SLC7A11 |
| 12_Member | GO Biological Processes | GO:0030168 | platelet activation | -2.236475184 | -0.582 | 4/124 | COL3A1, PF4, VWF, SLC7A11 |
| 13_Summary | KEGG Pathway | ko04145 | Phagosome | -5.490740628 | -2.824 | 8/154 | ATP6V1B1, HLA-DQB1, ITGA5, MRC1, MRC2, CLEC4M, PLA2R1, COLEC12, SIGLEC1, IFITM3, TRIM5, PF4, PSMA8 |
| 13_Member | KEGG Pathway | ko04145 | Phagosome | -5.490740628 | -2.824 | 8/154 | ATP6V1B1, HLA-DQB1, ITGA5, MRC1, MRC2, CLEC4M, PLA2R1, COLEC12 |
| 13_Member | KEGG Pathway | hsa04145 | Phagosome | -5.211124173 | -2.632 | 8/168 | ATP6V1B1, HLA-DQB1, ITGA5, MRC1, MRC2, CLEC4M, PLA2R1, COLEC12 |
| 13_Member | GO Biological Processes | GO:0046718 | viral entry into host cell | -3.701714339 | -1.610 | 6/145 | ITGA5, MRC1, SIGLEC1, CLEC4M, IFITM3, TRIM5 |
| 13_Member | GO Biological Processes | GO:0044409 | entry into host | -3.591055671 | -1.537 | 6/152 | ITGA5, MRC1, SIGLEC1, CLEC4M, IFITM3, TRIM5 |
| 13_Member | GO Biological Processes | GO:0052126 | movement in host environment | -3.252081509 | -1.284 | 6/176 | ITGA5, MRC1, SIGLEC1, CLEC4M, IFITM3, TRIM5 |
| 13_Member | GO Biological Processes | GO:0051701 | biological process involved in interaction with host | -2.919252904 | -1.056 | 6/204 | ITGA5, MRC1, SIGLEC1, CLEC4M, IFITM3, TRIM5 |
| 13_Member | GO Biological Processes | GO:0044403 | biological process involved in symbiotic interaction | -2.790156246 | -0.971 | 7/294 | ITGA5, MRC1, PF4, SIGLEC1, CLEC4M, IFITM3, TRIM5 |
| 13_Member | Reactome Gene Sets | R-HSA-1236978 | Cross-presentation of soluble exogenous antigens (endosomes) | -2.547298653 | -0.802 | 3/49 | MRC1, MRC2, PSMA8 |
| 13_Member | KEGG Pathway | ko05152 | Tuberculosis | -2.415445504 | -0.705 | 5/179 | HLA-DQB1, MRC1, MRC2, CLEC4M, PLA2R1 |
| 13_Member | KEGG Pathway | hsa05152 | Tuberculosis | -2.287278333 | -0.617 | 5/192 | HLA-DQB1, MRC1, MRC2, CLEC4M, PLA2R1 |
| 14_Summary | GO Biological Processes | GO:0003014 | renal system process | -5.450327437 | -2.792 | 7/110 | ATP6V1B1, BMP4, EDN1, GJA1, SLC4A1, AKR1C3, SLC22A6, SLC12A1, CYP1B1, NPHS2 |
| 14_Member | GO Biological Processes | GO:0003014 | renal system process | -5.450327437 | -2.792 | 7/110 | ATP6V1B1, BMP4, EDN1, GJA1, SLC4A1, AKR1C3, SLC22A6 |
| 14_Member | GO Biological Processes | GO:0006692 | prostanoid metabolic process | -2.547298653 | -0.802 | 3/49 | ATP6V1B1, EDN1, AKR1C3 |
| 14_Member | GO Biological Processes | GO:0006693 | prostaglandin metabolic process | -2.547298653 | -0.802 | 3/49 | ATP6V1B1, EDN1, AKR1C3 |
| 14_Member | GO Biological Processes | GO:0055078 | sodium ion homeostasis | -2.382625559 | -0.685 | 3/56 | ATP6V1B1, EDN1, SLC12A1 |
| 14_Member | GO Biological Processes | GO:0033559 | unsaturated fatty acid metabolic process | -2.33848428 | -0.655 | 4/116 | ATP6V1B1, CYP1B1, EDN1, AKR1C3 |
| 14_Member | GO Biological Processes | GO:0007588 | excretion | -2.258594482 | -0.596 | 3/62 | ATP6V1B1, EDN1, NPHS2 |
| 14_Member | GO Biological Processes | GO:0006690 | icosanoid metabolic process | -2.248801408 | -0.589 | 4/123 | ATP6V1B1, CYP1B1, EDN1, AKR1C3 |
| 15_Summary | GO Biological Processes | GO:0050886 | endocrine process | -5.063064423 | -2.519 | 6/83 | BMP6, CMA1, CPA3, EDN1, FOXD1, GJA1, ACTA2, KLK3, COL1A2, PPARG, BMP7, ELN, CCR2 |
| 15_Member | GO Biological Processes | GO:0050886 | endocrine process | -5.063064423 | -2.519 | 6/83 | BMP6, CMA1, CPA3, EDN1, FOXD1, GJA1 |
| 15_Member | GO Biological Processes | GO:0008217 | regulation of blood pressure | -4.888169972 | -2.412 | 8/186 | ACTA2, KLK3, CMA1, COL1A2, CPA3, EDN1, GJA1, PPARG |
| 15_Member | WikiPathways | WP3624 | Lung fibrosis | -4.497579439 | -2.166 | 5/63 | BMP7, CMA1, EDN1, ELN, CCR2 |
| 15_Member | GO Biological Processes | GO:0003073 | regulation of systemic arterial blood pressure | -3.624467627 | -1.553 | 5/96 | KLK3, CMA1, CPA3, EDN1, GJA1 |
| 15_Member | GO Biological Processes | GO:0001990 | regulation of systemic arterial blood pressure by hormone | -2.900371524 | -1.046 | 3/37 | CMA1, CPA3, EDN1 |
| 15_Member | GO Biological Processes | GO:0003044 | regulation of systemic arterial blood pressure mediated by a chemical signal | -2.599122228 | -0.840 | 3/47 | CMA1, CPA3, EDN1 |
| 16_Summary | GO Biological Processes | GO:0031347 | regulation of defense response | -4.781753893 | -2.366 | 14/625 | AOAH, KLK3, SERPING1, CASP4, CMA1, COCH, GJA1, MEFV, PPARG, CST7, AIM2, NLRC4, TRIM5, CCR2, CPA3 |
| 16_Member | GO Biological Processes | GO:0031347 | regulation of defense response | -4.781753893 | -2.366 | 14/625 | AOAH, KLK3, SERPING1, CASP4, CMA1, COCH, GJA1, MEFV, PPARG, CST7, AIM2, NLRC4, TRIM5, CCR2 |
| 16_Member | GO Biological Processes | GO:0016485 | protein processing | -2.684345715 | -0.896 | 6/227 | KLK3, CASP4, CMA1, CPA3, CST7, NLRC4 |
| 16_Member | GO Biological Processes | GO:0050727 | regulation of inflammatory response | -2.126541848 | -0.506 | 7/389 | AOAH, CASP4, CMA1, MEFV, PPARG, CST7, CCR2 |
| 16_Member | GO Biological Processes | GO:0051604 | protein maturation | -2.125612512 | -0.506 | 6/296 | KLK3, CASP4, CMA1, CPA3, CST7, NLRC4 |
| 17_Summary | GO Biological Processes | GO:0019221 | cytokine-mediated signaling pathway | -4.701049974 | -2.320 | 12/472 | CASP4, EDN1, IL7, PF4, PPARG, CCL15, AIM2, IFITM3, IL17RB, CARD14, IL31RA, CCR2, COL1A1 |
| 17_Member | GO Biological Processes | GO:0019221 | cytokine-mediated signaling pathway | -4.701049974 | -2.320 | 12/472 | CASP4, EDN1, IL7, PF4, PPARG, CCL15, AIM2, IFITM3, IL17RB, CARD14, IL31RA, CCR2 |
| 17_Member | GO Biological Processes | GO:0071356 | cellular response to tumor necrosis factor | -2.665294407 | -0.886 | 6/229 | CASP4, COL1A1, EDN1, CCL15, AIM2, CARD14 |
| 17_Member | GO Biological Processes | GO:0001961 | positive regulation of cytokine-mediated signaling pathway | -2.52224746 | -0.783 | 3/50 | CASP4, EDN1, IL7 |
| 17_Member | GO Biological Processes | GO:0034612 | response to tumor necrosis factor | -2.451516764 | -0.733 | 6/253 | CASP4, COL1A1, EDN1, CCL15, AIM2, CARD14 |
| 17_Member | GO Biological Processes | GO:0060760 | positive regulation of response to cytokine stimulus | -2.360961922 | -0.671 | 3/57 | CASP4, EDN1, IL7 |
| 18_Summary | GO Biological Processes | GO:0048251 | elastic fiber assembly | -4.662102198 | -2.287 | 3/10 | LOX, MYH11, FBLN5, COL1A2 |
| 18_Member | GO Biological Processes | GO:0048251 | elastic fiber assembly | -4.662102198 | -2.287 | 3/10 | LOX, MYH11, FBLN5 |
| 18_Member | GO Biological Processes | GO:0085029 | extracellular matrix assembly | -3.883266039 | -1.714 | 4/45 | COL1A2, LOX, MYH11, FBLN5 |
| 19_Summary | GO Biological Processes | GO:0071396 | cellular response to lipid | -4.517088331 | -2.177 | 13/575 | BMP4, BMP6, BMP7, CASR, COL1A1, EDN1, GJB2, MRC1, PF4, WNT6, AKR1C3, ALDH1A2, TRIM5, CYP1B1, FBLN5, PLA2R1, DCXR, CDH1, PLN, ADCY10, SLC5A5, COLEC12, LOX, SLC7A11, PPARG |
| 19_Member | GO Biological Processes | GO:0071396 | cellular response to lipid | -4.517088331 | -2.177 | 13/575 | BMP4, BMP6, BMP7, CASR, COL1A1, EDN1, GJB2, MRC1, PF4, WNT6, AKR1C3, ALDH1A2, TRIM5 |
| 19_Member | GO Biological Processes | GO:0072593 | reactive oxygen species metabolic process | -3.313386014 | -1.319 | 7/239 | BMP7, CYP1B1, EDN1, AKR1C3, FBLN5, PLA2R1, DCXR |
| 19_Member | GO Biological Processes | GO:0010035 | response to inorganic substance | -3.28892508 | -1.307 | 11/574 | BMP6, BMP7, CASR, CDH1, COL1A1, CYP1B1, EDN1, PLN, AKR1C3, FBLN5, ADCY10 |
| 19_Member | GO Biological Processes | GO:0071407 | cellular response to organic cyclic compound | -3.220852899 | -1.257 | 11/585 | BMP4, BMP7, CASR, CDH1, COL1A1, CYP1B1, EDN1, GJB2, SLC5A5, AKR1C3, COLEC12 |
| 19_Member | GO Biological Processes | GO:0048545 | response to steroid hormone | -3.093362666 | -1.169 | 8/339 | BMP4, BMP6, BMP7, COL1A1, EDN1, GJB2, LOX, AKR1C3 |
| 19_Member | GO Biological Processes | GO:0006979 | response to oxidative stress | -2.908638979 | -1.049 | 9/450 | BMP7, COL1A1, CYP1B1, EDN1, GJB2, AKR1C3, FBLN5, PLA2R1, SLC7A11 |
| 19_Member | GO Biological Processes | GO:0034599 | cellular response to oxidative stress | -2.823983282 | -0.993 | 7/290 | BMP7, CYP1B1, GJB2, AKR1C3, FBLN5, PLA2R1, SLC7A11 |
| 19_Member | GO Biological Processes | GO:0000302 | response to reactive oxygen species | -2.723070295 | -0.919 | 6/223 | BMP7, COL1A1, CYP1B1, EDN1, AKR1C3, FBLN5 |
| 19_Member | GO Biological Processes | GO:2000377 | regulation of reactive oxygen species metabolic process | -2.66000724 | -0.884 | 5/157 | BMP7, CYP1B1, AKR1C3, FBLN5, DCXR |
| 19_Member | GO Biological Processes | GO:0031960 | response to corticosteroid | -2.544099672 | -0.801 | 5/167 | BMP6, COL1A1, EDN1, GJB2, AKR1C3 |
| 19_Member | KEGG Pathway | ko04913 | Ovarian steroidogenesis | -2.52224746 | -0.783 | 3/50 | BMP6, CYP1B1, AKR1C3 |
| 19_Member | GO Biological Processes | GO:0062197 | cellular response to chemical stress | -2.445690748 | -0.728 | 7/339 | BMP7, CYP1B1, GJB2, AKR1C3, FBLN5, PLA2R1, SLC7A11 |
| 19_Member | KEGG Pathway | hsa04913 | Ovarian steroidogenesis | -2.404719756 | -0.699 | 3/55 | BMP6, CYP1B1, AKR1C3 |
| 19_Member | GO Biological Processes | GO:0019748 | secondary metabolic process | -2.278319214 | -0.610 | 3/61 | CYP1B1, AKR1C3, SLC7A11 |
| 19_Member | GO Biological Processes | GO:0071384 | cellular response to corticosteroid stimulus | -2.278319214 | -0.610 | 3/61 | EDN1, GJB2, AKR1C3 |
| 19_Member | GO Biological Processes | GO:0071383 | cellular response to steroid hormone stimulus | -2.178005798 | -0.543 | 5/204 | BMP4, BMP7, EDN1, GJB2, AKR1C3 |
| 19_Member | GO Biological Processes | GO:0010565 | regulation of cellular ketone metabolic process | -2.130518611 | -0.508 | 4/133 | BMP6, PPARG, AKR1C3, SLC7A11 |
| 19_Member | GO Biological Processes | GO:0006801 | superoxide metabolic process | -2.046320033 | -0.441 | 3/74 | BMP7, EDN1, FBLN5 |
| 20_Summary | GO Biological Processes | GO:0032526 | response to retinoic acid | -4.432203168 | -2.108 | 6/107 | BMP6, COL1A1, GJA1, GJB2, WNT6, ALDH1A2, BMP7, FOXD1, CASR, PPARG, AKR1C3, ALDH1A3, ADGRG6, BMP4 |
| 20_Member | GO Biological Processes | GO:0032526 | response to retinoic acid | -4.432203168 | -2.108 | 6/107 | BMP6, COL1A1, GJA1, GJB2, WNT6, ALDH1A2 |
| 20_Member | WikiPathways | WP5052 | Nephrogenesis | -3.84429923 | -1.694 | 3/18 | BMP7, FOXD1, ALDH1A2 |
| 20_Member | GO Biological Processes | GO:0007584 | response to nutrient | -3.304703439 | -1.319 | 6/172 | BMP7, CASR, COL1A1, PPARG, AKR1C3, ALDH1A2 |
| 20_Member | GO Biological Processes | GO:0031076 | embryonic camera-type eye development | -2.935266963 | -1.067 | 3/36 | ALDH1A3, BMP7, ALDH1A2 |
| 20_Member | GO Biological Processes | GO:0032355 | response to estradiol | -2.864777787 | -1.021 | 5/141 | BMP7, COL1A1, GJA1, GJB2, ALDH1A2 |
| 20_Member | GO Biological Processes | GO:0033273 | response to vitamin | -2.718433743 | -0.918 | 4/91 | BMP7, CASR, COL1A1, ALDH1A2 |
| 20_Member | GO Biological Processes | GO:0061383 | trabecula morphogenesis | -2.710396091 | -0.915 | 3/43 | BMP7, COL1A1, ADGRG6 |
| 20_Member | GO Biological Processes | GO:0060324 | face development | -2.653411406 | -0.879 | 3/45 | ALDH1A3, COL1A1, ALDH1A2 |
| 20_Member | GO Biological Processes | GO:0010718 | positive regulation of epithelial to mesenchymal transition | -2.473751672 | -0.748 | 3/52 | BMP4, BMP7, COL1A1 |
| 20_Member | GO Biological Processes | GO:0071300 | cellular response to retinoic acid | -2.183084726 | -0.545 | 3/66 | COL1A1, WNT6, ALDH1A2 |
