# Supplementary material for: Neural EGFL-like 1, a craniosynostosis-related osteochondrogenic molecule, strikingly associates with neurodevelopmental pathologies
Source: Cell Biosci. 2023 Dec 15;13:227. doi: 10.1186/s13578-023-01174-5 (PMC10725010; doi:10.1186/s13578-023-01174-5)
Supplement: Supplementary file 12 — Additional file 12: Table S5.The converting results of the input upregulated DEGs in the Metascape. [file 13578_2023_1174_MOESM12_ESM.docx]

Table S5. The converting results of the input upregulated DEGs in the Metascape. The DEGs are first converted into their corresponding H. sapiens Entrez gene IDs using the latest version of the database (last updated on 2021-08-01). If multiple identifiers correspond to the same Entrez gene ID, they will be considered as a single Entrez gene ID in downstream analyses.

| **MyList** | **Gene ID** | **Type** | **Tax ID** | **Homologene Gene ID** | **Homologene Gene Tax ID** | **Gene Symbol** | **Description** |
| --- | --- | --- | --- | --- | --- | --- | --- |
| *Ccdc83* | 75338 | symbol | *M. musculus* | 220047 | *H. sapiens* | *CCDC83* | *coiled-coil domain containing 83* |
| *Hc* | 15139 | symbol | *M. musculus* | 727 | *H. sapiens* | *C5* | *complement C5* |
| *Snord14c* | 108637 | symbol | *M. musculus* |  |  | None | None |
| *Gm15577* | 100502679 | symbol | *M. musculus* |  |  | None | None |
| *Spata31d1a* | 72219 | symbol | *M. musculus* | 389763 | *H. sapiens* | *SPATA31D1* | *SPATA31 subfamily D member 1* |
| *Slamf1* | 27218 | symbol | *M. musculus* | 6504 | *H. sapiens* | *SLAMF1* | *signaling lymphocytic activation molecule family member 1* |
| *Snrpn* | 20646 | symbol | *M. musculus* | 6638 | *H. sapiens* | *SNRPN* | *small nuclear ribonucleoprotein polypeptide N* |
| *Gm20255* | 100504496 | symbol | *M. musculus* |  |  | None | None |
| *Qrfprl* | 243407 | symbol | *M. musculus* | 7852 | *H. sapiens* | *CXCR4* | *C-X-C motif chemokine receptor 4* |
| *Dao* | 13142 | symbol | *M. musculus* | 1610 | *H. sapiens* | *DAO* | *D-amino acid oxidase* |
| *Aunip* | 69885 | symbol | *M. musculus* | 79000 | *H. sapiens* | *AUNIP* | *aurora kinase A and ninein interacting protein* |
| *Spem1* | 74288 | symbol | *M. musculus* | 374768 | *H. sapiens* | *SPEM1* | *spermatid maturation 1* |
| *Eef1akmt3* | 100504608 | symbol | *M. musculus* | 25895 | *H. sapiens* | *EEF1AKMT3* | *EEF1A lysine methyltransferase 3* |
| *Mir540* | 723880 | symbol | *M. musculus* |  |  | None | None |
| *Lhx5* | 16873 | symbol | *M. musculus* | 64211 | *H. sapiens* | *LHX5* | *LIM homeobox 5* |
| *Hoxa7* | 15404 | symbol | *M. musculus* | 3204 | *H. sapiens* | *HOXA7* | *homeobox A7* |
| *Il4* | 16189 | symbol | *M. musculus* | 3565 | *H. sapiens* | *IL4* | *interleukin 4* |
| *Best1* | 24115 | symbol | *M. musculus* | 7439 | *H. sapiens* | *BEST1* | *bestrophin 1* |
| *Gm19422* | 100502869 | symbol | *M. musculus* |  |  | None | None |
| *Gm7902* | 666038 | symbol | *M. musculus* |  |  | None | None |
| *Irx5* | 54352 | symbol | *M. musculus* | 10265 | *H. sapiens* | *IRX5* | *iroquois homeobox 5* |
| *Mir3070b* | 100526519 | symbol | *M. musculus* |  |  | None | None |
| *Gm5083* | 328235 | symbol | *M. musculus* |  |  | None | None |
| *Gm10604* | 100038424 | symbol | *M. musculus* |  |  | None | None |
| *Chat* | 12647 | symbol | *M. musculus* | 1103 | *H. sapiens* | *CHAT* | *choline O-acetyltransferase* |
| *Tcam1* | 75870 | symbol | *M. musculus* | 3384 | *H. sapiens* | *ICAM2* | *intercellular adhesion molecule 2* |
| *Gm8971* | 668090 | symbol | *M. musculus* |  |  | None | None |
| *Kbtbd6* | 432879 | symbol | *M. musculus* | 89890 | *H. sapiens* | *KBTBD6* | *kelch repeat and BTB domain containing 6* |
| *Tlr1* | 21897 | symbol | *M. musculus* | 7096 | *H. sapiens* | *TLR1* | *toll like receptor 1* |
| *Ppef2* | 19023 | symbol | *M. musculus* | 5470 | *H. sapiens* | *PPEF2* | *protein phosphatase with EF-hand domain 2* |
| *Bhlha15* | 17341 | symbol | *M. musculus* | 168620 | *H. sapiens* | *BHLHA15* | *basic helix-loop-helix family member a15* |
| *Rassf6* | 73246 | symbol | *M. musculus* | 166824 | *H. sapiens* | *RASSF6* | *Ras association domain family member 6* |
| *Ubash3a* | 328795 | symbol | *M. musculus* | 53347 | *H. sapiens* | *UBASH3A* | *ubiquitin associated and SH3 domain containing A* |
| *Slamf9* | 98365 | symbol | *M. musculus* | 89886 | *H. sapiens* | *SLAMF9* | *SLAM family member 9* |
| *Tmem190* | 78052 | symbol | *M. musculus* | 147744 | *H. sapiens* | *TMEM190* | *transmembrane protein 190* |
| *4833417C18Rik* | 73906 | symbol | *M. musculus* |  |  | None | None |
| *3110053B16Rik* | 382686 | symbol | *M. musculus* |  |  | None | None |
| *2610037D02Rik* | 70040 | symbol | *M. musculus* |  |  | None | None |
| *Gm5802* | 545062 | symbol | *M. musculus* |  |  | None | None |
| *Opn1sw* | 12057 | symbol | *M. musculus* | 611 | *H. sapiens* | *OPN1SW* | *opsin 1, short wave sensitive* |
| *9230116N13Rik* | 320054 | symbol | *M. musculus* |  |  | None | None |
| *Snord34* | 27210 | symbol | *M. musculus* |  |  | None | None |
| *Scnn1b* | 20277 | symbol | *M. musculus* | 6338 | *H. sapiens* | *SCNN1B* | *sodium channel epithelial 1 subunit beta* |
| *Gbp11* | 634650 | symbol | *M. musculus* | 163351 | *H. sapiens* | *GBP6* | *guanylate binding protein family member 6* |
| *Tm4sf5* | 75604 | symbol | *M. musculus* | 9032 | *H. sapiens* | *TM4SF5* | *transmembrane 4 L six family member 5* |
| *A930006K02Rik* | 100503120 | symbol | *M. musculus* |  |  | None | None |
| *Dnah7a* | 627872 | symbol | *M. musculus* | 56171 | *H. sapiens* | *DNAH7* | *dynein axonemal heavy chain 7* |
| *Gm4926* | 237749 | symbol | *M. musculus* |  |  | None | None |
| *Rpl29* | 19944 | symbol | *M. musculus* | 6159 | *H. sapiens* | *RPL29* | *ribosomal protein L29* |
| *9230009I02Rik* | 619293 | symbol | *M. musculus* |  |  | None | None |
| *Lvrn* | 74574 | symbol | *M. musculus* | 206338 | *H. sapiens* | *LVRN* | *laeverin* |
| *Ndn* | 17984 | symbol | *M. musculus* | 4692 | *H. sapiens* | *NDN* | *necdin, MAGE family member* |
| *5730408K05Rik* | 67531 | symbol | *M. musculus* |  |  | None | None |
| *1500035N22Rik* | 70258 | symbol | *M. musculus* |  |  | None | None |
| *Cdc6* | 23834 | symbol | *M. musculus* | 990 | *H. sapiens* | *CDC6* | *cell division cycle 6* |
| *Fcgbp* | 215384 | symbol | *M. musculus* | 8857 | *H. sapiens* | *FCGBP* | *Fc fragment of IgG binding protein* |
| *Irx1* | 16371 | symbol | *M. musculus* | 79192 | *H. sapiens* | *IRX1* | *iroquois homeobox 1* |
| *Acot10* | 64833 | symbol | *M. musculus* | 23597 | *H. sapiens* | *ACOT9* | *acyl-CoA thioesterase 9* |
| *Rec114* | 73673 | symbol | *M. musculus* | 283677 | *H. sapiens* | *REC114* | *REC114 meiotic recombination protein* |
| *Actrt3* | 76652 | symbol | *M. musculus* | 84517 | *H. sapiens* | *ACTRT3* | *actin related protein T3* |
| *Slc45a2* | 22293 | symbol | *M. musculus* | 51151 | *H. sapiens* | *SLC45A2* | *solute carrier family 45 member 2* |
| *Acsbg3* | 78625 | symbol | *M. musculus* | 51703 | *H. sapiens* | *ACSL5* | *acyl-CoA synthetase long chain family member 5* |
| *Igbp1b* | 50540 | symbol | *M. musculus* | 3476 | *H. sapiens* | *IGBP1* | *immunoglobulin binding protein 1* |
| *Gm12945* | 100048926 | symbol | *M. musculus* |  |  | None | None |
| *Rrm2* | 20135 | symbol | *M. musculus* | 6241 | *H. sapiens* | *RRM2* | *ribonucleotide reductase regulatory subunit M2* |
| *Gm10724* | 100038688 | symbol | *M. musculus* |  |  | None | None |
| *Dcst2* | 329702 | symbol | *M. musculus* | 127579 | *H. sapiens* | *DCST2* | *DC-STAMP domain containing 2* |
| *Nkain3* | 269513 | symbol | *M. musculus* | 286183 | *H. sapiens* | *NKAIN3* | *sodium/potassium transporting ATPase interacting 3* |
| *Mroh5* | 268816 | symbol | *M. musculus* | 83876 | *H. sapiens* | *MRO* | *maestro* |
