# Supplementary material for: Neural EGFL-like 1, a craniosynostosis-related osteochondrogenic molecule, strikingly associates with neurodevelopmental pathologies
Source: Cell Biosci. 2023 Dec 15;13:227. doi: 10.1186/s13578-023-01174-5 (PMC10725010; doi:10.1186/s13578-023-01174-5)
Supplement: Supplementary file 13 — Additional file 13: Table S6.The result of pathway and process enrichment analysis with the upregulated DEGs. [file 13578_2023_1174_MOESM13_ESM.docx]

Table S6. The result of pathway and process enrichment analysis with the upregulated DEGs.

| **GroupID** | **Category** | **Term** | **Description** | **Log_10_P** | **Log_10_(q-value)** | **InTerm_InList** | **Symbols** | |
| --- | --- | --- | --- | --- | --- | --- | --- | --- |
| 1_Summary | WikiPathways | WP3998 | Prader-Willi and Angelman syndrome | -5.379911507 | -1.018 | 4/66 | | CDC6, NDN, SNRPN, SLC45A2 |
| 1_Member | WikiPathways | WP3998 | Prader-Willi and Angelman syndrome | -5.379911507 | -1.018 | 4/66 | | CDC6, NDN, SNRPN, SLC45A2 |
| 2_Summary | GO Biological Processes | GO:0007601 | visual perception | -4.579882414 | -0.657 | 5/215 | | OPN1SW, PPEF2, BEST1, IRX5, SLC45A2, CXCR4, TLR1 |
| 2_Member | GO Biological Processes | GO:0007601 | visual perception | -4.579882414 | -0.657 | 5/215 | | OPN1SW, PPEF2, BEST1, IRX5, SLC45A2 |
| 2_Member | GO Biological Processes | GO:0050953 | sensory perception of light stimulus | -4.541586901 | -0.657 | 5/219 | | OPN1SW, PPEF2, BEST1, IRX5, SLC45A2 |
| 2_Member | GO Biological Processes | GO:0009581 | detection of external stimulus | -2.851775201 | 0.000 | 3/135 | | OPN1SW, BEST1, CXCR4 |
| 2_Member | GO Biological Processes | GO:0009582 | detection of abiotic stimulus | -2.824410052 | 0.000 | 3/138 | | OPN1SW, BEST1, CXCR4 |
| 2_Member | GO Biological Processes | GO:0051606 | detection of stimulus | -2.29136751 | 0.000 | 5/687 | | OPN1SW, PPEF2, TLR1, BEST1, CXCR4 |
| 3_Summary | GO Biological Processes | GO:0050900 | leukocyte migration | -3.463386826 | 0.000 | 5/372 | | C5, HOXA7, IL4, SLAMF1, CXCR4, SCNN1B, TLR1, UBASH3A |
| 3_Member | GO Biological Processes | GO:0050900 | leukocyte migration | -3.463386826 | 0.000 | 5/372 | | C5, HOXA7, IL4, SLAMF1, CXCR4 |
| 3_Member | GO Biological Processes | GO:0002685 | regulation of leukocyte migration | -3.400397587 | 0.000 | 4/212 | | C5, HOXA7, IL4, SLAMF1 |
| 3_Member | GO Biological Processes | GO:0002275 | myeloid cell activation involved in immune response | -3.320964151 | 0.000 | 3/93 | | IL4, SCNN1B, SLAMF1 |
| 3_Member | GO Biological Processes | GO:0050920 | regulation of chemotaxis | -3.310276735 | 0.000 | 4/224 | | C5, IL4, SLAMF1, CXCR4 |
| 3_Member | GO Biological Processes | GO:0002274 | myeloid leukocyte activation | -3.303002524 | 0.000 | 4/225 | | IL4, SCNN1B, SLAMF1, TLR1 |
| 3_Member | GO Biological Processes | GO:0030595 | leukocyte chemotaxis | -3.25303949 | 0.000 | 4/232 | | C5, IL4, SLAMF1, CXCR4 |
| 3_Member | GO Biological Processes | GO:0002683 | negative regulation of immune system process | -3.164086032 | 0.000 | 5/433 | | C5, HOXA7, IL4, SLAMF1, UBASH3A |
| 3_Member | GO Biological Processes | GO:0002688 | regulation of leukocyte chemotaxis | -2.968079957 | 0.000 | 3/123 | | C5, IL4, SLAMF1 |
| 3_Member | GO Biological Processes | GO:0050921 | positive regulation of chemotaxis | -2.797669638 | 0.000 | 3/141 | | IL4, SLAMF1, CXCR4 |
| 3_Member | GO Biological Processes | GO:0060326 | cell chemotaxis | -2.776700969 | 0.000 | 4/312 | | C5, IL4, SLAMF1, CXCR4 |
| 3_Member | GO Biological Processes | GO:0032640 | tumor necrosis factor production | -2.496743323 | 0.000 | 3/180 | | IL4, SLAMF1, TLR1 |
| 3_Member | GO Biological Processes | GO:0032680 | regulation of tumor necrosis factor production | -2.496743323 | 0.000 | 3/180 | | IL4, SLAMF1, TLR1 |
| 3_Member | GO Biological Processes | GO:0071706 | tumor necrosis factor superfamily cytokine production | -2.45674547 | 0.000 | 3/186 | | IL4, SLAMF1, TLR1 |
| 3_Member | GO Biological Processes | GO:1903555 | regulation of tumor necrosis factor superfamily cytokine production | -2.45674547 | 0.000 | 3/186 | | IL4, SLAMF1, TLR1 |
| 3_Member | GO Biological Processes | GO:0071674 | mononuclear cell migration | -2.393085267 | 0.000 | 3/196 | | IL4, SLAMF1, CXCR4 |
| 3_Member | GO Biological Processes | GO:0097529 | myeloid leukocyte migration | -2.237424407 | 0.000 | 3/223 | | C5, IL4, SLAMF1 |
| 3_Member | GO Biological Processes | GO:0002443 | leukocyte mediated immunity | -2.235974422 | 0.000 | 4/442 | | C5, IL4, SCNN1B, SLAMF1 |
| 3_Member | GO Biological Processes | GO:0001819 | positive regulation of cytokine production | -2.152899748 | 0.000 | 4/467 | | C5, IL4, SLAMF1, TLR1 |
| 3_Member | GO Biological Processes | GO:0001817 | regulation of cytokine production | -2.021189638 | 0.000 | 5/799 | | C5, IL4, SLAMF1, TLR1, UBASH3A |
| 4_Summary | GO Biological Processes | GO:0009165 | nucleotide biosynthetic process | -2.082362079 | 0.000 | 3/254 | | IL4, RRM2, ACSL5, ACOT9 |
| 4_Member | GO Biological Processes | GO:0009165 | nucleotide biosynthetic process | -2.082362079 | 0.000 | 3/254 | | IL4, RRM2, ACSL5 |
| 4_Member | GO Biological Processes | GO:0009117 | nucleotide metabolic process | -2.080906657 | 0.000 | 4/490 | | IL4, RRM2, ACOT9, ACSL5 |
| 4_Member | GO Biological Processes | GO:1901293 | nucleoside phosphate biosynthetic process | -2.073085467 | 0.000 | 3/256 | | IL4, RRM2, ACSL5 |
| 4_Member | GO Biological Processes | GO:0006753 | nucleoside phosphate metabolic process | -2.056785646 | 0.000 | 4/498 | | IL4, RRM2, ACOT9, ACSL5 |
| 5_Summary | GO Biological Processes | GO:0048871 | multicellular organismal homeostasis | -2.018542594 | 0.000 | 4/511 | | IL4, NDN, SCNN1B, CXCR4 |
| 5_Member | GO Biological Processes | GO:0048871 | multicellular organismal homeostasis | -2.018542594 | 0.000 | 4/511 | | IL4, NDN, SCNN1B, CXCR4 |
