# Supplementary material for: Neural EGFL-like 1, a craniosynostosis-related osteochondrogenic molecule, strikingly associates with neurodevelopmental pathologies
Source: Cell Biosci. 2023 Dec 15;13:227. doi: 10.1186/s13578-023-01174-5 (PMC10725010; doi:10.1186/s13578-023-01174-5)
Supplement: Supplementary file 14 — Additional file 14: Fig. S7. The demography of alternative splice events detected by CASH. [file 13578_2023_1174_MOESM14_ESM.docx]

**Additional Information**

for

Neural EGFL like 1, a craniosynostosis-related osteochondrogenic molecule, strikingly associates with neurodevelopmental pathologies

Chenshuang Li, Zhong Zheng, Pin Ha, Wenlu Jiang, Chia Soo, Kang Ting

Correspondence to: Chia Soo (bsoo@g.ucla.edu); Kang Ting (erickangting@gmail.com)

**This document includes:**

Materials and Methods

Figures S1 to S7

Tables S1 to S7

Captions for Videos S1 to S4

Additional References

**Other Additional information for this manuscript include the following:**

Videos S1 to S4

Materials and Methods

Animal maintenance

All the experiments on live mice were performed under an institutionally approved protocol provided by the Chancellor’s Animal Research Committee at UCLA (protocol numbers: 2014-041 and 2013-013). Due to N-ethyl-N-nitrosourea (ENU)-induced homozygous Nell-1-deficient (Nell-1^6R/6R^) mice having a severely reduced expression of Nell-1 that results in neonatal death (1-4), Nell-1-haploinsufficient (Nell-1^+/6R^) mice (a well-established loss-of-function model (2-4)) were examined in the current investigation. Mice were bred and maintained as previously described (4), and their genotypes were determined by polymerase chain reaction. All the behavioral tests were conducted in a blinded fashion, and decoding was performed after all the drug administration and behavioral tests were performed.

Microcomputed tomography (micro-CT)

Skeletons of 3-month-old mouse craniums were imaged *ex vivo* in a high-resolution micro-CT scanner (Skyscan 1172; Bruker-microCT, Konitch, Belgium) at an image resolution of 20 μm (44 kV and 139 mA radiation source, 0.5 mm aluminum filter). All resulting images were reconstructed by NRecon (Bruker-microCT) and visualized in DataViewer (Bruker-microCT) (5).

Grooming assessment

3-month-old mice were scored on a scale from 1–4 depending on the extent of their grooming based on the scoring system set up previously (6). In brief, the score 1 means that the whiskers are trimmed all the way to the skin, the score 2 indicates that the hairs around whiskers are also gone; the score 3 represents that a more extensive and spread facial hair loss is noticed, and the score 4 reflects the situation that additional body parts experience hair loss as well.

Marble-burying test

Mice were placed in the middle of a cage (27 × 16.5 × 12.5 cm) with 5 cm high corncob bedding that had 20 black marbles of ∼14 mm diameter gently placed in a 4 × 5 arrangement. The mice were video-monitored for 10 min (6). The extent of marble-burying was assessed by the number of marbles completely buried in the corncob bedding materials.

Three-chamber social interaction test

Mice were habituated to the test chamber and allowed to freely explore for a set time. After habituation, one testing mouse was placed in the central chamber. A wild-type (WT) unfamiliar mouse were randomly placed in one of the two wire cups in enclosures, and the percentage of time the tested mouse spends in the chamber with the WT mouse cup will be compared to the time spent in the chamber with the empty cup (7).

Open field arena (OFA) test

OFA test was conducted in an activity test chamber (27.3 cm × 27.3 cm) in a self-enclosed sound-attenuating cubicle (Med Associates, St. Albans, VT) with 16 infrared light beams per side. Mice were released into the center of the test chamber. The activity of the mouse over 20 minutes were determined by beam breaks and recorded by computer for subsequent analysis. The apparatus was thoroughly cleaned with 70% isopropanol before each mouse is tested. The periphery and center are arbitrarily defined, and the dependent variables measured are distance traveled, and time in the periphery *vs.* time in the center (either in the central 66% area of the whole test chamber, or in the central 50% area of the whole test chamber). Illumination levels during testing were maintained at a constant 60 lx (6).

Elevated plus maze (EPM) test

The EPM was consist of four white, equally spaced arms, 39 cm in height and 33.9 cm from the center of the apparatus. Two opposing arms were enclosed by white walls extending 15.3 cm above the surface, and two arms were open. Individual mice were placed in the center of the maze to start, and their activity recorded on a computer for 5 min by a video camera. Animals were released using a bottomless holding chamber at the start of the test. The animals' movements were captured and analyzed. The apparatus was thoroughly cleaned with 70% isopropanol before each mouse was tested. Dependent variables are distance covered, the duration of stretched attend posture (SAP), and time spent in the open *vs.* closed arms. Illumination levels during testing were maintained at a constant 195 lx (6).

Startle response and pre-pulse inhibition (PPI) evaluation

The PPI test was performed with the same setting described previously for *Cntnap4*-deficient mice evaluation (6) to allow the comparison between *Cntnap4*-deficient mice and *Nell-1^+/6R^* mice. In brief, each mouse was placed into a Plexiglas cylinder attached to a piezoelectric sensor. The startle response to an acoustic stimulus was measured in the presence of a 65-dB white noise background that began a 5-min acclimation period. Each session consisted of a randomized block design of 40 trials that present a 20 ms pre-pulse of 74, 82, or 90 dB followed 100 ms later by either a 40 ms 120 dB startle pulse or no pulse at all. The inter-trail interval was an average of 15 s but was pseudo-randomized during presentations. The apparatus was thoroughly cleaned with 70% isopropanol before each mouse is tested. For each trial, the percent PPI for each pre-pulse startle stimulus was determined by Equation 1. The mean response for each group was calculated for each pre-pulse stimulus level.

PPI (%) = 100 − (pre-pulse startle response/startle response) × 100 Equation 1

Rotarod performance assessment

Mice were placed on a cylinder that can rotate at accelerating speeds. The rotarod was set with a start speed of 4 rpm, acceleration rate of 20 rpm/min. 4 trials per test were performed with a 2 min interval between trails. The time the mice can stay on the cylinder and the revolutions per minute were counted (8).

Fear conditioning test

Mice were placed into a conditioning chamber and are given parings of a conditioned stimulus (an auditory cue) and an aversive unconditioned stimulus (an electric foot shock). For delay fear conditioning, the 30-sec conditioned stimulus co-terminated with a 2-sec 0.35-mA unconditioned stimulus. Under the trace fear conditioning paradigm, the 30-sec conditioned stimulus was followed by a 30-sec trace interval and then the 2-sec unconditioned stimulus. Freezing behavior during the test were measured as an index of fear memory (9). Freezing was defined as the absence of all movement except respiration.

Drug administration

Risperidone, a Food and Drug Administration (FDA) approved drug that can reverse all the ASD-like behaviors in mice (7). was chosen in the current study. Since oral gavage could potentially cause stress in the mice (10) and thus alter the mice behaviors, the intraperitoneal injection approach was selected to avoid the potential stress of oral gavage administration. Briefly, Risperidone (0.2 mg/kg, Sigma) was administered by a daily intraperitoneal injection in a volume of 10 mL/kg for 7 consecutive days (days 1-7). Behavioral tests were performed on days 8, 9, and 10. Mice also received drug treatment during these days approximately 1 hour prior to behavioral tests (7).

Statistical analysis of behavioral tests

Since some behavioral tests significantly interfere with each other, different mouse cohorts were used. All the data were presented as raw data overlapped with median ± 95% confidence interval, and the Mann-Whitney *U* test was used for intergroup comparisons. For all data presented in this manuscript, *P* < 0.05 (*) was considered a suggestive difference, while *P* < 0.005 (**) was recognized as a statistically significant difference based on a recent recommendation (11).

Dissection of mouse hippocampus for RNA isolation

3-month-old *Nell-1^+/6R^* mice (3 males and 3 females) and their WT littermates (3 males and 3 females) were anesthetized and decapitated to harvest the hippocampus. After disinfection with 70% ethanol, the skin of the head was dissected at room temperature in a laminar flow sterile hood to expose the skull. Next, scissors were inserted into the spinal canal, and the calvarium was carefully cut on one side close to the front to avoid damaging the brain. The same procedure was repeated on the contralateral side. The base of the skull was lifted by forceps to expose the brain, which was subsequently transferred to a dissection dish with ice-cold Hank's essential medium. The hippocampus was then quickly dissected following a commonly acceptable protocol (12). The RNA was isolated by using the RNeasy Lipid Tissue Mini Kit with DNase (QIAGEN Sciences, Maryland, USA).

Whole transcriptome sequencing and differentially expressed genes (DEGs) analysis

The whole transcriptome was sequenced on an Illumina HiSeq 3000 system (13). Data analyses were performed on the Galaxy platform(14) (UseGalaxy.org) with an established, broadly validated protocol (15-17). Briefly, the FASTQC RNA‐seq reads were aligned to the mouse genome (mm10) using HISAT2 aligner (Galaxy Version 2.1.0+galaxy 5) with default parameters (18). Raw counts of sequencing read for the feature of genes were extracted by *featureCounts* (Galaxy Version 1.6.4+galaxy1) (19). Then, the *limma* package (Galaxy version 3.38.3 + galaxy3) was used to identify DEGs with its *voom* method (20, 21). Expressed genes were selected as their counts per million (CPM), value not less than 0.2 in at least two samples across the entire experiment, while lowly expressed genes were removed for the flowing analyses. Benjamini–Hochberg correction was employed in the *limma‐voom* analysis for *P* value adjustment (22), which is highly recommended by the *limma* user guide (23). Quasi‐likelihood F‐tests (ANOVA‐like analysis) were achieved to identify DEGs (24). Genes with fold change (FC) more than 1.5 and *P* value less than 0.05 were assigned as DEGs. Heatmap diagrams were conducted in *R* (version 3.6.3) (25) with packages *pheatmap* (version 1.0.12).

Alternative splicing (AS) analysis

AS is a post-transcriptional process with critical, wide-ranging effects on a plethora of cellular activities and disease processes (26-31). The current study used comprehensive alternative splicing hunting (CASH) method for AS analysis with the default settings following the manual (32, 33). The types of alternative splice events detected by CASH are demonstrated in **Additional** **file 18, Fig. S7**. Events with *P* < 0.05 and |ΔPSI| > 0.2 were considered significant.

Enrichment

Pathway enrichment of identified DEGs were performed in the Metascape (34). In brief, pathway and process enrichment analysis had been carried out with the following ontology sources: KEGG Pathway, GO Biological Processes, Reactome Gene Sets, Canonical Pathways, CORUM, TRRUST, DisGeNET, PaGenBase, Transcription Factor Targets, WikiPathways, PANTHER Pathway and COVID. All genes in the genome had been used as the enrichment background. Terms with a Metascape systemic default parameter settings (*P*-value < 0.01, a minimum count of 3, and an enrichment factor > 1.5 [the enrichment factor is the ratio between the observed counts and the counts expected by chance]) were collected and grouped into clusters based on their membership similarities. More specifically *P*-values were calculated based on the accumulative hypergeometric distribution, and *q*-values were calculated using the Banjamini-Hochberg procedure to account for multiple testings (35). Kappa scores were used as the similarity metric when performing hierarchical clustering on the enriched terms, and sub-trees with a similarity of > 0.3 were considered a cluster. The most statistically significant term within a cluster was chosen to represent the cluster.
