# Supplementary material for: Neural EGFL-like 1, a craniosynostosis-related osteochondrogenic molecule, strikingly associates with neurodevelopmental pathologies
Source: Cell Biosci. 2023 Dec 15;13:227. doi: 10.1186/s13578-023-01174-5 (PMC10725010; doi:10.1186/s13578-023-01174-5)
Supplement: Supplementary file 15 — Additional file 15: Table S7.The alternative splicing gene list with a P-value less than 0.05. [file 13578_2023_1174_MOESM15_ESM.docx]

Table S7. The alternative splicing gene list with a P-value less than 0.05. “Het” represents the Nell-1^+/6R^ group and the “WT” represents the wildtype group.

| **AccID** | **Location** | **Exon** | **Het_Junc_ Inclusive::Exclusive** | **WT_Junc_ Inclusive::Exclusive** | **Het_Exp_ Inclusive::Exclusive** | **WT_Exp_ Inclusive::Exclusive** | **∆_PSI** | ***P*-Value** | **FDR** | **Splicing Type** |
| --- | --- | --- | --- | --- | --- | --- | --- | --- | --- | --- |
| *Pcp2* | chr8:3623371-3624632 | 5 | 8::24 | 11::0 | 16::39 | 16::15 | -0.75 | 0.00426945 | 0.978799131 | IR |
| *Gm1821* | chr14:46084558-46084812 | 2 | 0::305 | 44::44 | 8::129 | 50::12 | -0.498146775 | 2.52E-34 | 2.98E-30 | A3SS |
| *Snrpn* | chr7:60007499-60071965 | 2-3 | 0::36 | 22::23 | 6::46 | 6::16 | -0.488888889 | 0.022770692 | 0.978799131 | AltStart |
| *Spock1* | chr13:57696110-57907587 | 3 | 15::20 | 26::5 | 12::174 | 17::146 | -0.410138249 | 0.037205516 | 0.978799131 | Cassette |
| *Snrpn* | chr7:60047889-60132397 | 2-3 | 8::28 | 17::10 | 7::57 | 10::19 | -0.407407407 | 0.001350505 | 0.978799131 | AltStart |
| *Whrn* | chr4:63419422-63432059 | 13 | 24::25 | 28::9 | 16::41 | 20::35 | -0.266960838 | 0.042685084 | 0.978799131 | Cassette_multi |
| *Ilf3* | chr9:21387700-21388753 | 6 | 18::30 | 34::19 | 15::79 | 25::84 | -0.266509434 | 0.041545481 | 0.978799131 | Cassette |
| *Cadps* | chr14:12468346-12472406 | 25-26 | 6::12 | 17::12 | 8::34 | 19::25 | -0.252873563 | 0.044830937 | 0.978799131 | MXE |
| *Wtap* | chr17:12992077-12992546 | 2 | 23::49 | 39::40 | 15::17 | 23::13 | -0.174226442 | 0.044288154 | 0.978799131 | AltStart |
| *Dock9* | chr14:121542506-121546104 | 60 | 58::23 | 133::17 | 26::287 | 38::261 | -0.170617284 | 0.014373019 | 0.978799131 | Cassette |
| *Flad1* | chr3:89405855-89405953 | 6 | 38::12 | 73::6 | 65::84 | 87::77 | -0.164050633 | 0.044496182 | 0.978799131 | A3SS |
| *Fau* | chr19:6057888-6058325 | 1 | 108::96 | 183::85 | 63::131 | 104::133 | -0.153424056 | 0.01273498 | 0.978799131 | IR |
| *Rab26* | chr17:24529741-24529970 | 8 | 87::29 | 98::17 | 41::158 | 50::110 | -0.102173913 | 0.046618563 | 0.978799131 | IR |
| *Gm1821* | chr14:46084128-46084812 | 1 | 1887::742 | 1760::769 | 46::15 | 15::30 | 0.021351238 | 0.015897764 | 0.978799131 | IR |
| *Ptgds* | chr2:25466709-25467345 | 7 | 1131::82 | 1683::192 | 146::1730 | 184::2872 | 0.03474501 | 0.015273223 | 0.978799131 | IR |
| *Cltb* | chr13:54593538-54598833 | 5 | 416::69 | 300::78 | 235::516 | 188::484 | 0.064081165 | 0.046058842 | 0.978799131 | Cassette |
| *Gm47283* | chrY:90793418-90816464 | 6-7 | 415::538 | 323::627 | 52::9 | 38::12 | 0.094995688 | 0.02186138 | 0.978799131 | AltEnd |
| *Snf8* | chr11:96043404-96043502 | 6 | 14::89 | 2::102 | 41::145 | 29::176 | 0.116691561 | 0.028504669 | 0.978799131 | A5SS |
| *Dlg3* | chrX:100809862-100812776 | 18 | 147::75 | 111::94 | 78::156 | 58::159 | 0.120698748 | 0.033803424 | 0.978799131 | Cassette |
| *Nrxn1* | chr17:90454806-90560860 | 20 | 12::21 | 13::43 | 34::20 | 19::23 | 0.131493506 | 0.049950856 | 0.978799131 | AltStart |
| *Fam155a* | chr8:9207238-9233105 | 7 | 57::111 | 31::125 | 38::214 | 21::203 | 0.140567766 | 0.015434846 | 0.978799131 | Cassette |
| *Rars2* | chr4:34623411-34630570 | 5 | 47::0 | 29::5 | 23::24 | 36::24 | 0.147058824 | 0.040119859 | 0.978799131 | Cassette |
| *Cad* | chr5:31075089-31075853 | 37 | 8::26 | 2::34 | 11::31 | 6::36 | 0.179738562 | 0.049051807 | 0.978799131 | Cassette |
| *Tlk1* | chr2:70757474-70770075 | 4 | 18::31 | 6::45 | 13::41 | 9::50 | 0.24969988 | 0.030883486 | 0.978799131 | Cassette |
| *Tbc1d24* | chr17:24183694-24186201 | 7 | 22::0 | 9::4 | 19::40 | 12::41 | 0.307692308 | 0.03184081 | 0.978799131 | Cassette |
| *Nsmf* | chr2:25057965-25058321 | 6 | 30::18 | 5::15 | 171::867 | 111::762 | 0.375 | 0.011437075 | 0.978799131 | MXE |
| *Snrpn* | chr7:60071885-60140219 | 6-7 | 32::1 | 3::7 | 85::28 | 23::12 | 0.66969697 | 0.043685613 | 0.978799131 | AltStart |
